# Integrated Multi-Omics Characterization of *WASHC2C* Reveals Cancer-Specific Molecular, Clinical, Immune, and Cellular Associations

**DOI:** 10.3390/cimb48101046

**Published:** 2026-10-09

**Authors:** Hakeemah H. Al-Nakhle

**Affiliations:** 1 Department of Clinical Laboratory Sciences, College of Applied Medical Sciences, Taibah University, Madinah 42353, Saudi Arabia; hnakhly@taibahu.edu.sa; 2 Health and Life Research Center, Taibah University, Madinah 42353, Saudi Arabia

**Keywords:** *WASHC2C*, *FAM21C*, WASH complex, multi-omics, pan-cancer analysis, tumor microenvironment, immune-cell infiltration, copy-number variation, DNA methylation, single-cell analysis

## Abstract

*WASHC2C* encodes a WASH-complex scaffold implicated in actin remodeling, endosomal organization, and intracellular trafficking, yet its cancer-specific molecular and clinical relevance remains insufficiently defined, particularly in the context of the closely related paralogue *WASHC2A*. An integrated pan-cancer multi-omics analysis was performed incorporating transcriptomic and protein-level evidence, clinicopathological and survival associations, genomic and epigenetic alterations, pathway activity, gene-set enrichment, immune features, immunotherapy-related observations, and single-cell functional states. Primary RNA analyses extracted both paralogues from the same GDC STAR files across eight cancer types, while covariate-adjusted Cox models evaluated LUAD, BLCA, and LIHC. A locked 50-gene co-expression signature was additionally assessed in independent GEO cohort, GSE31210. The harmonized expression analysis comprised 4365 primary tumors and 429 normal specimens and revealed pronounced lineage-specific variation together with strong *WASHC2A–WASHC2C* co-expression. Although HNSC showed platform-dependent directional differences between STAR and GSCA, the integrated analyses identified LIHC as the clearest prognostic context: higher *WASHC2C* expression was associated with poorer overall survival in the primary adjusted model (340 patients; 115 deaths; HR = 1.61 per unit log2[TPM + 1], 95% CI 1.18–2.21; FDR = 0.035), although the magnitude of association varied with model specification. Favorable unadjusted observations in LUAD and BLCA were not retained after covariate adjustment, and primary BLCA OS/DSS models were non-estimable. Across cancers, copy-number variation showed a consistent positive relationship with transcript abundance, whereas pathway, immune, methylation, and single-cell associations demonstrated marked tumor-context specificity. In GSE31210, 48 measurable genes from the locked signature were associated with favorable survival in 204 patients, supporting the relevance of the broader co-expression program, although neither *WASHC2C* nor *WASHC2A* contributed directly to that score. Collectively, this study establishes a rigorous multi-omic framework for *WASHC2C*, highlights its lineage-dependent molecular and prognostic relevance, and prioritizes specific cancer contexts—particularly LIHC—for mechanistic and translational investigation. These findings provide a strong foundation for future paralogue-resolved functional studies and independent clinical validation.

## 1. Introduction

Cancer remains a major global health challenge, with approximately 20 million new cases and 9.7 million deaths estimated worldwide in 2022 [1,2]. Its clinical burden is compounded by extensive biological heterogeneity arising from tissue of origin, genomic and epigenetic alterations, cellular plasticity, microenvironmental selection, and treatment-driven evolution [3]. These interconnected factors generate substantial variation both among cancer types and between patients with the same malignancy. Consequently, molecular abnormalities identified using a single analytical layer may incompletely represent tumor biology and may not retain equivalent biological or clinical relevance across different tumor contexts.

Multi-omics analysis has therefore become increasingly important in precision oncology because it integrates complementary molecular dimensions, including genomics, transcriptomics, proteomics, epigenomics, and cellular phenotyping. Unlike single-modality studies, multi-omics approaches can determine whether an observed transcriptional alteration is supported by genomic regulation, protein-level changes, pathway activity, or clinically relevant phenotypes. Such integration can also identify molecular discordance, which may reflect post-transcriptional regulation, altered protein stability, epigenetic control, cellular heterogeneity, or microenvironmental influences. Pan-cancer multi-omics strategies are particularly valuable for distinguishing recurrent molecular mechanisms from tissue-specific alterations and for identifying candidate biomarkers whose relevance depends on tumor lineage or biological context [4,5,6,7,8,9,10,11].

*WASHC2C*, officially designated WASH complex subunit 2C and previously annotated as *FAM21C*, is a protein-coding gene located on chromosome 10q11.22. It has also been reported under alternative names, including *KIAA0592* and *VPEF* [12,13]. The encoded protein is broadly expressed and is predominantly associated with intracellular vesicular structures, although cytosolic and nucleolar localization has also been reported [14,15]. Importantly, earlier studies frequently used the general designation FAM21 without clearly distinguishing among closely related *WASHC2* paralogues. Evidence specifically involving *WASHC2C* must therefore be differentiated from findings concerning other FAM21-family proteins or the WASH regulatory complex as a whole.

Functionally, *WASHC2C* is a core component of the heteropentameric WASH regulatory complex, an actin-regulatory assembly primarily associated with endosomal membranes [16,17,18,19]. Whereas WASHC1 contains the principal VCA domain responsible for activating the Arp2/3 complex, *WASHC2C* primarily functions as a large scaffold and trafficking adaptor. Its extended C-terminal region interacts with retromer-associated proteins, phosphoinositide-enriched membranes, and actin-capping machinery, thereby contributing to WASH-complex recruitment, organization, and recycling [19,20,21,22,23,24]. Through these interactions, the WASH machinery coordinates endosomal actin polymerization, membrane tubule fission, cargo sorting, receptor recycling, retrograde transport, lysosomal homeostasis, and vesicular trafficking. Structural studies have also shown how FAM21-family sequences connect the WASH complex with the SNX27–retromer system, providing a molecular basis for selective recycling of membrane proteins and signaling receptors [24].

These cellular processes are highly relevant to malignant transformation and tumor progression. Actin-cytoskeleton remodeling contributes to changes in cell morphology, adhesion, polarity, migration, invasion, and metastatic dissemination. Similarly, endosomal trafficking and receptor recycling regulate the abundance, localization, and signaling duration of growth-factor receptors, integrins, transporters, and adhesion molecules. Dysregulation of these systems may sustain proliferative and survival signaling, modify nutrient acquisition, facilitate extracellular-matrix interactions, and contribute to resistance to targeted therapies. Disturbed endosome–lysosome dynamics may additionally influence autophagy, proteostasis, antigen processing, and membrane turnover. Nevertheless, functions established for the wider WASH, retromer, or sorting-nexin systems cannot automatically be attributed specifically to *WASHC2C* [23,25,26].

Direct experimental evidence linking *WASHC2C* to cancer remains limited but biologically informative. In hepatocellular carcinoma, *FAM21C/WASHC2C* was reported to promote invasion and metastasis by remodeling the actin cytoskeleton and inhibiting CAPZA1-mediated actin capping [27]. A nuclear pool of *FAM21* has also been implicated in NF-κB-dependent transcription and drug sensitivity in pancreatic cancer cells [28]. By contrast, studies associating *WASHC1* overexpression with cancer stem-cell properties and poor prognosis in esophageal carcinoma concern another WASH-complex component and provide only indirect support for *WASHC2C*-related cancer biology [29]. Similarly, findings indicating that FAM21-family proteins participate in dendritic-cell trafficking and receptor-dependent immune functions support biological plausibility but do not establish a specific role for *WASHC2C* in tumor immunity [30].

A multi-omics framework enables systematic integration of *WASHC2C*-associated transcriptomic patterns with clinical, genomic, proteomic, and cellular features across cancer types. Protein-level measurements provide complementary biological context; however, the high sequence similarity among closely related paralogues may limit the ability to attribute antibody-based or proteomic signals specifically to *WASHC2C*. Integration of gene expression with pathological stage, molecular subtype, and survival outcomes can identify clinically relevant associations [31]. Nevertheless, concordance across independent resources should not be interpreted as evidence of assay specificity, mechanistic causality, or established clinical utility.

Somatic mutations, copy-number alterations, and DNA methylation provide additional layers of molecular characterization [32,33,34]. Associations between copy-number variation and transcript abundance may indicate gene-dosage effects, whereas tumor–normal methylation comparisons can reveal cancer-associated epigenetic differences. However, the present study did not directly assess methylation–expression relationships or broader measures of genomic instability; accordingly, these findings are interpreted as complementary molecular associations rather than evidence of direct regulatory mechanisms.

The tumor microenvironment adds biological complexity, reflecting dynamic interactions among malignant, immune, and stromal cell populations [35,36,37,38]. Given the established roles of actin remodeling and vesicular trafficking in immune-cell migration, receptor recycling, and antigen processing, evaluation of *WASHC2C*-associated immune patterns is biologically justified [30,39]. Associations with inferred immune-cell infiltration, immune-checkpoint genes, chemokines, and antigen-presentation machinery may therefore provide insight into tumor-specific immune contexts [40]. These relationships remain observational, however, and cannot establish direct immunoregulatory activity. Similarly, exploratory treatment-response findings derived from mouse models provide useful preclinical context [41].

Pathway-activity profiling, gene-set enrichment analysis, and gene-set variation analysis can further characterize the biological programs associated with variation in *WASHC2C* expression [42,43]. Single-cell analyses provide complementary resolution by linking gene expression to functional cellular states, although they remain sensitive to transcript dropout, cell-type annotation, sample composition, and inter-dataset heterogeneity [44,45,46]. 

The present study integrates transcriptomic expression, clinical outcome, somatic mutation, copy-number alteration, DNA methylation, pathway activity, immune features, and single-cell functional states within a unified pan-cancer framework. Its principal contribution is interpreting these multidimensional associations alongside a harmonized *WASHC2A*–*WASHC2C* paralogue comparison, covariate-adjusted survival analyses, and independent evaluation of a predefined co-expression signature. 

## 2. Materials and Methods

### 2.1. Study Design and Gene Nomenclature

An integrated pan-cancer analysis was conducted to characterize the transcriptional, proteomic, clinical, genomic, epigenetic, immunological, and single-cell associations of WASH complex subunit 2C. The current HGNC-approved symbol, *WASHC2C*, was used throughout the manuscript. The former symbol, *FAM21C*, was retained only when reporting results from databases or antibodies annotated under the previous nomenclature. *WASHC2C* corresponds to NCBI Gene ID 253725 and Ensembl gene ID ENSG00000172661.

**Figure 1:**
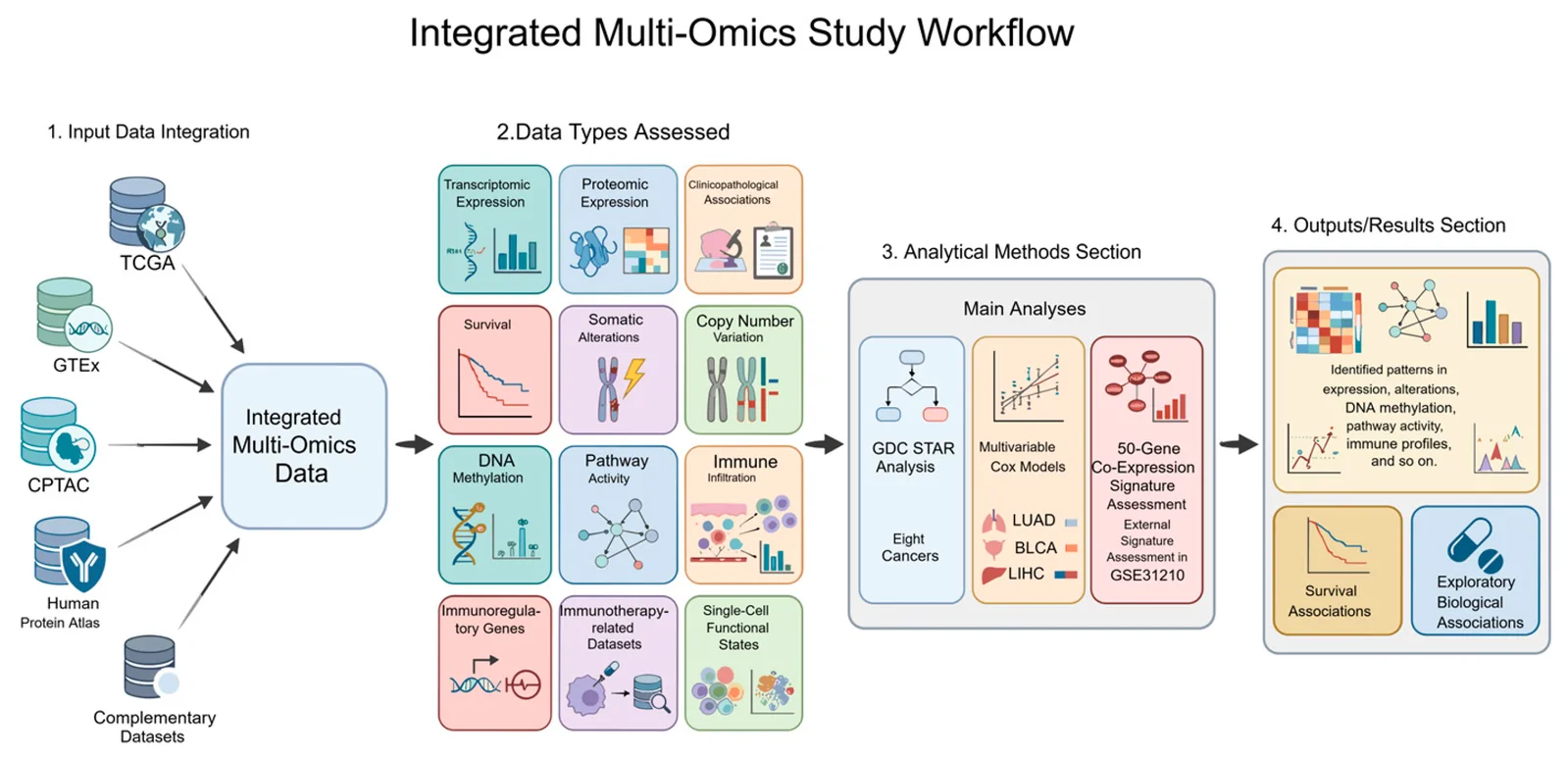
Overview of the integrated multi-omics workflow for pan-cancer characterization of *WASHC2C*. (1) Data were compiled from The Cancer Genome Atlas (TCGA), Genotype-Tissue Expression (GTEx), Clinical Proteomic Tumor Analysis Consortium (CPTAC), Human Protein Atlas, and complementary datasets. (2) Analyses covered transcriptomic and proteomic expression, clinicopathological and survival associations, somatic alterations, copy number variation, DNA methylation, pathway activity, immune infiltration, immunoregulatory genes, immunotherapy-related datasets, and single-cell functional states. (3) Selected analyses included assessment of Genomic Data Commons (GDC) STAR RNA-sequencing data across eight cancer types; multivariable Cox regression in lung adenocarcinoma (LUAD), bladder urothelial carcinoma (BLCA), and liver hepatocellular carcinoma (LIHC); and external assessment of a 50-gene co-expression signature in GSE31210. (4) Results were synthesized to describe molecular patterns, survival associations, and exploratory biological relationships involving *WASHC2C.* Created in BioRender. Al-nakhle, H. (2026) https://BioRender.com/niddraz.

Publicly available data from The Cancer Genome Atlas (TCGA), the Genotype-Tissue Expression (GTEx) project, the Clinical Proteomic Tumor Analysis Consortium (CPTAC), the Human Protein Atlas (HPA), Gene Set Cancer Analysis (GSCA), the Shiny Methylation Analysis Resource Tool (SMART) App, Tumor Immune Estimation Resource 2.0 (TIMER2.0), Tumor Immune Syngeneic MOuse (TISMO), and Cancer Single-Cell State Atlas (CancerSEA) were analyzed. The analyses used de-identified public data. Figure 1 shows the study workflow.

#### 2.1.1. Data Processing and Statistical Analysis

Molecular datasets were analyzed separately using resource-specific processed data; no single cross-platform expression matrix or global batch correction was applied. Uniform processing of TCGA–GTEx data reduces processing variation but does not eliminate differences in tissue collection, donor characteristics, or other batch effects. The same-file GDC STAR analysis is the primary RNA comparison for the eight prespecified cancers. Two-sided tests and Benjamini–Hochberg (BH) correction were applied to documented comparison families. Primary inferential conclusions require FDR < 0.05. 

#### 2.1.2. Assessment of Paralogue Specificity

Harmonized GDC STAR-Counts files using GENCODE v36 were analyzed for BLCA, BRCA, COAD, HNSC, LIHC, LUAD, LUSC, and PRAD. *WASHC2A* (ENSG00000099290.17) and *WASHC2C* (ENSG00000172661.19) were extracted from the same selected files, yielding 4365 primary tumors and 429 solid-tissue normal specimens. Primary-tumor Spearman correlations were BH-adjusted across eight cancers. Full-cohort Mann–Whitney tests were BH-adjusted across 16 gene–cancer comparisons; log2 ratios of mean TPM and descriptive bootstrap confidence intervals were reported separately. Matched-pair Wilcoxon signed-rank tests on log2(TPM + 1) differences were adjusted across 16 comparisons. Full-cohort tests did not model dependence between paired specimens. Selection rules, sample counts, effect-size definitions, and sensitivity analyses appear in Supplementary Materials Section S1.1 and Tables S1–S5.

Theoretical sequence distinguishability was evaluated with GENCODE v36 transcripts and the GRCh38 primary assembly at sequence lengths of 75, 100, and 150 bases. Exact-match sequence proportions are not observed in uniquely mapped patient-read fractions.

### 2.2. Data Acquisition and Gene Expression Analysis

Normal-tissue expression was examined through GTEx (https://gtexportal.org/home/; accessed 20 November 2025). Secondary pan-cancer expression views used TIMER2.0 [40], GDC, and uniformly processed TCGA–GTEx data accessed through UCSC Xena [47] (https://xenabrowser.net/datapages/; accessed 20 November 2025). These resource-specific views are distinct from the primary eight-cancer STAR analysis.

### 2.3. Evaluation of Differential WASHC2C Expression

Secondary expression comparisons used the GSCA single-gene Expression module [48] (https://guolab.wchscu.cn/GSCA/; accessed 25 March 2026), which provides RSEM-normalized TCGA expression data. The displayed cohorts were BLCA, BRCA, COAD, HNSC, KICH, LIHC, LUAD, LUSC, and PRAD. 

The GSCA output compares tumor and normal expression using Wilcoxon rank-sum tests and reports BH-adjusted FDR. The log2 fold-change display and RSEM distributions were retained as secondary observations. Differences from the same-file STAR comparison were reported explicitly rather than interpreted as technical replication.

### 2.4. CPTAC Protein-Abundance Observations

CPTAC protein-abundance data [8] were examined through UALCAN [49] (https://ualcan.path.uab.edu/; accessed 23 February 2026) for BRCA, COAD, HNSC, LIHC, LUAD and UCEC. *WASHC2C* was queried under the former symbol *FAM21C*. 

### 2.5. Illustrative HPA Family Immunoreactivity

The HPA Tissue and Pathology Atlases [14] (https://www.proteinatlas.org/; accessed 20 June 2026) were used for descriptive illustrations of *WASHC2A*/*WASHC2C*-family immunoreactivity. The lung panels include adenocarcinoma and squamous cell carcinoma. The kidney tumor is labeled kidney adenocarcinoma in the image and is not assigned to KICH.

Staining was reviewed descriptively using the image annotations; no inferential comparison or *WASHC2C*-specific protein validation was performed. HPA reports 100% *WASHC2A* antigen identity for HPA047844, HPA060975 and HPA061022. 

### 2.6. Survival Analysis of WASHC2C Expression

The prognostic relevance of *WASHC2C* expression was assessed across TCGA cohorts using GSCA clinical and expression data. The evaluated endpoints were overall survival (OS), progression-free survival (PFS), disease-specific survival (DSS), and disease-free interval (DFI). Patients without valid survival time, event status, or expression data were excluded from the corresponding endpoint analysis.

Univariate Cox proportional hazards regression was performed for each cancer type, with *WASHC2C* expression as the explanatory variable. Hazard ratios and corresponding *p* values were recorded. For Kaplan–Meier analysis, patients were divided into high- and low-expression groups using the cohort-specific median *WASHC2C* expression value unless otherwise specified. Survival distributions were compared using the two-sided log-rank test. Kaplan–Meier curves were generated with censoring indicated by vertical marks, and median survival was displayed when reached. The GSCA framework uses Cox regression, Kaplan–Meier estimation, and log-rank testing for survival analyses.

#### Multivariable Cox Regression

GDC-harmonized STAR-TPM expression from UCSC Xena [47] was linked to the TCGA Clinical Data Resource (TCGA-CDR) [31] for LUAD, BLCA, and LIHC. *WASHC2C* was identified as ENSG00000172661.19. Primary tumors were matched by patient barcode, retaining one sample per patient by lexicographic barcode order. No GTEx samples were included. The source log2(TPM + 1) values were analyzed without further transformation.

Primary complete-case Cox models included continuous *WASHC2C*, age per 10 years, sex, and categorical pathological stage, with no imputation. *WASHC2C* HRs represent a one-unit increase in log2(TPM + 1). OS, progression-free interval (PFI), DSS, and DFI followed TCGA-CDR definitions; PFI is distinguished from the PFS label retained in GSCA figures. Efron ties, Wald 95% confidence intervals, and two-sided Wald *p* values were used. BH correction covered 12 planned *WASHC2C* tests; non-estimable BLCA OS/DSS tests occupied *p* = 1 slots for correction only and have no estimated HR or inferential *p* value. Stage-stratified and 90-day DFI landmark results are reported separately. The STAR/CDR cohorts differ from the earlier analysis in processing and complete-case selection; changes in results cannot be attributed solely to covariate adjustment.

### 2.7. Association Between WASHC2C Expression and Tumor Stage

Pathological and clinical-stage information was obtained from TCGA through GSCA. Undefined, missing, stage 0, stage X, and nonstandard stage annotations were excluded. The remaining cases were grouped into stages I, II, III, and IV. Differences across stage groups were examined using the GSCA Kruskal–Wallis output and pairwise Wilcoxon comparisons. 

### 2.8. WASHC2C Expression and Oncogenic Pathway Activity

Associations between *WASHC2C* expression and major oncogenic pathway activities were evaluated using the GSCA Expression and Pathway Activity module. GSCA pathway scores are derived from reverse-phase protein-array data obtained from The Cancer Proteome Atlas and include the apoptosis, cell-cycle, DNA-damage-response, epithelial–mesenchymal-transition, hormone androgen-receptor, hormone estrogen-receptor, PI3K/AKT, RAS/MAPK, receptor tyrosine kinase, and TSC/mTOR pathways.

GSCA pathway scores combine positive and negative protein regulators, with the latter contributing inversely. Scores were compared between cohort-median *WASHC2C* expression groups using the Wilcoxon output and its FDR annotations. 

### 2.9. Predefined WASHC2C-Associated Coexpression Signature

A predefined 50-gene *WASHC2C*-associated co-expression signature was retained with its original Ensembl membership. The source table reports Pearson-labeled correlation coefficients and identifies *WASHC2A* as the highest-ranked gene (PCC 0.80). *WASHC2C* itself is not a signature member. GEPIA2 is the reported source resource [50], but the exact query settings and generation history are not established by the retained table. Accordingly, no genome-wide Spearman/FDR thresholding or cross-cancer frequency-selection procedure is asserted. The complete membership was locked before GSE31210 evaluation, without external reselection or substitution.

Sample-level enrichment scores were obtained using GSVA through GSCA [42,48]. GSVA summarizes relative enrichment of the predefined gene set within each sample; it is not a direct measurement of *WASHC2C* expression or proof of coordinated regulation.

TCGA signature-survival views used cohort-median score groups, univariate Cox models, and log-rank comparisons for OS, PFS, DSS, and DFI. These discovery-cohort analyses are exploratory and separate from the adjusted single-gene models.

#### External Signature Assessment

The archived external protocol designated GSE31210 as the secondary OS [51,52]. All 50 Ensembl identities were locked without reselection, substitution, or cutoff optimization. Coverage was 47/50, 48/50, and 49/50 genes, respectively. *WASHC2C* was not a signature member. *WASHC2A* and *PARGP1* were unavailable in GSE31210 under conservative annotation-based mapping. Ambiguous probes were excluded, eligible probes per gene were aggregated by their within-sample median, and invariant or incomplete rows were removed. GSE31210 MAS5 intensities were log2-transformed; source-normalized log2 values were retained for the other cohorts. Scoring used the full eligible gene background within each cohort, without TCGA pooling.

GSVA used a Gaussian kernel, tau = 1, maximum-difference scoring, and no absolute-rank transformation. All 226 GSE31210 tumors were scored against 19,759 background genes; 22 source-defined exclusions left 204 survival-eligible patients with 30 deaths and 54 relapse events. Continuous scores were standardized within eligible cohorts. Cox models used Efron ties and complete cases. GSE31210 OS was stage-adjusted because of the prespecified events-per-parameter rule, and relapse models included age, sex, stage, and smoking. Median groups were used only for Kaplan–Meier plots. Schoenfeld tests, stage stratification, and ssGSEA assessed sensitivity. 

### 2.10. Gene Set Enrichment Analysis

Gene set enrichment analysis was conducted to determine whether the predefined *WASHC2C*-associated signature was coordinately enriched or depleted in tumor relative to normal tissue across the selected cancer types. Genes were ranked by their tumor-versus-normal differential expression statistic, and enrichment was assessed using the GSCA GSEA module.

Normalized enrichment scores (NES) indicate the direction of enrichment: positive values favor tumor tissue and negative values favor normal tissue. The retained GSCA display reports adjusted *p* values. The original ranked lists, permutation settings, and complete correction family were not retained; these secondary findings are interpreted descriptively.

### 2.11. Correlation Between GSVA Scores and Pathway Activity

The signaling context of the *WASHC2C*-associated signature was evaluated by calculating Spearman’s rank correlations between GSVA scores and the 10 GSCA oncogenic pathway activity scores for each TCGA cancer type. The retained GSCA displays report Spearman coefficients and FDR values for signature–pathway associations. 

### 2.12. Somatic Single-Nucleotide-Variant Analysis

The pan-cancer *WASHC2C* somatic-variant landscape was analyzed through the GSCA Mutation module using processed TCGA somatic-mutation data. The analysis included nonsynonymous variants classified as missense mutations, nonsense mutations, frameshift insertions, frameshift deletions, splice-site variants, and in-frame insertions or deletions.

For each cancer type, the *WASHC2C* mutation frequency was calculated as the number of samples carrying at least one eligible somatic variant divided by the total number of samples with available mutation data. Variants were summarized according to functional classification, variant type, and single-nucleotide substitution class. The number and classes of variants carried by each mutation-positive sample were additionally recorded.

For survival analysis, patients were categorized as *WASHC2C*-mutant if at least one eligible nonsynonymous *WASHC2C* variant was detected, and as wild type if no such variant was detected. Univariate Cox regression and Kaplan–Meier analyses were performed for DFI, DSS, OS, and PFS. Mutant-versus-wild-type survival distributions were compared using the log-rank test. Analyses with inadequate numbers of mutant cases or outcome events were omitted or interpreted descriptively.

To reconcile the UCEC cohorts, mutation data were obtained from the GSCA-processed MC3 v0.2.8 PUBLIC dataset (Synapse syn7824274; GRCh37). For the corrected progression-free survival (PFS) analysis, one primary tumor per patient was retained, and records without a valid endpoint time or binary event status were excluded.

PFS was defined using the GSCA pfs_days and pfs_status fields, and survival time was converted to months by dividing days by 30. These fields were confirmed to match the PFS.time and PFS columns, respectively, in the TCGA Clinical Data Resource (TCGA-CDR) ExtraEndpoints worksheet for all 548 UCEC records. Under this definition, a qualifying new tumor event or death from any cause was counted as an event; otherwise, patients were censored. In the reconciled cohort, 24 additional deaths were classified as events compared with the TCGA-CDR progression-free interval (PFI) endpoint. PFS and PFI were therefore treated as distinct endpoints. Kaplan–Meier estimates were recalculated, a two-sided log-rank test was performed, and an unadjusted Cox proportional hazards model was fitted using the Efron method for ties. The corresponding 95% confidence intervals were calculated using the Wald method.

### 2.13. Copy-Number-Alteration Analysis

Processed TCGA copy-number data generated using GISTIC2.0 were analyzed through GSCA. Gene-level copy-number states were classified as homozygous deletion, heterozygous deletion, copy-number neutral, heterozygous amplification, or homozygous amplification according to the categories provided by the platform.

The frequencies of heterozygous and homozygous *WASHC2C* alterations were calculated separately for each cancer type. For the survival analysis, samples were grouped into amplification, deletion, or wild-type/copy-number-neutral according to the GSCA gene-level CNV classification. Patients lacking CNV or survival information were excluded.

Survival differences among amplification, deletion, and copy-number-neutral groups were examined using Kaplan–Meier curves and log-rank tests. Group sizes and nominal *p* values are reported; sparse groups were retained for descriptive presentation and interpreted cautiously.

### 2.14. Correlation Between WASHC2C Copy Number and mRNA Expression

Spearman rank correlations between gene-level *WASHC2C* copy-number values and log2-transformed RSEM expression were evaluated within TCGA cohorts using the GSCA output.

### 2.15. DNA Methylation Analysis

The DNA methylation landscape of *WASHC2C* was assessed using TCGA Illumina HumanMethylation450 BeadChip data accessed through the Shiny Methylation Analysis Resource Tool App (SMART) (http://www.bioinfo-zs.com/smartapp, accessed on 20 May 2026) [34]. 

CpG probes were mapped relative *to WASHC2C* and annotated CpG islands, shores and shelves. Beta values range from 0 to 1. The N-shore probe cg15344919 was used as a selected representative probe for site-specific illustration; a mechanistic selection rationale was not established. 

### 2.16. Correlation Between WASHC2C and Immune-Regulatory Genes

The association between *WASHC2C* expression and selected immune-regulatory genes was evaluated using TCGA expression data. The prespecified panel included chemokines and chemokine receptors—*CCL2*, *CCL5*, *CXCL9*, *CXCL10*, *CXCL12*, *CCR2*, *CCR5*, and *CXCR4*; immune-checkpoint genes—*CD274*, *CD276*, *PDCD1*, *CTLA4*, *LAG3*, *TIGIT*, *HAVCR2*, and *PDCD1LG2*; and antigen-presentation genes—*HLA-A*, *HLA-B*, *HLA-C*, and *B2M*.

Within each TCGA cohort, Spearman correlation coefficients were calculated between *WASHC2C* and each immune-regulatory gene using log2-transformed expression values. Cancer subgroups, including HPV-positive and HPV-negative HNSC and the evaluated BRCA subgroups, were analyzed separately when the required subtype annotations were available.

### 2.17. Immune and Stromal Infiltration Analysis

Relationships between *WASHC2C* expression and tumor-infiltrating immune or stromal populations were investigated using the TIMER2.0 Gene module (http://timer.cistrome.org/, accessed on 20 June 2026). Infiltration estimates were obtained from TIMER, EPIC, MCP-counter, quanTIseq, CIBERSORT, CIBERSORT-ABS, xCell, and TIDE where available. The evaluated populations included CD8^+^ T cells, CD4^+^ T-cell subsets, B cells, macrophage and monocyte subsets, natural killer cells, and cancer-associated fibroblasts.

TIMER2.0 purity-adjusted Spearman partial correlations were summarized using the heatmaps and representative scatterplots. Crosses and displayed *p* values refer to nominal tests, not BH-adjusted significance. Differences among algorithms were considered descriptively; no quantitative algorithm-agreement statistic or complete FDR-adjusted infiltration matrix is reported. Correlation patterns were interpreted by cancer, cell subset, and algorithm.

### 2.18. Exploratory Mouse Washc2 Treatment Response Analysis

Mouse Washc2 expression was examined in TISMO syngeneic tumor models [41]. These preclinical analyses concern mouse Washc2, not human *WASHC2C* expression or clinical response prediction.

### 2.19. Single-Cell Functional-State Analysis

Single-cell *WASHC2C* functional-state associations were examined using CancerSEA [44] (http://biocc.hrbmu.edu.cn/CancerSEA/; accessed 20 June 2026). The 14 states were angiogenesis, apoptosis, cell cycle, differentiation, DNA damage, DNA repair, epithelial–mesenchymal transition, hypoxia, inflammation, invasion, metastasis, proliferation, quiescence, and stemness.

The CancerSEA data summarizes gene–state correlations, and the resource describes Spearman tests with BH correction and thresholds of correlation magnitude > 0.30 and FDR < 0.05 [44]. 

### 2.20. Reproducibility and Analysis Materials

The retained TCGA Cox and external-signature analysis packages are provided in Supplementary Materials S1, organized by module. The TCGA Cox package contains executable scripts, source and processed data, patient selection/exclusion logs, model outputs, diagnostics, and checksums. The external-signature package additionally contains frozen GEO matrices and platform annotations, the locked 50-gene definition, full gene backgrounds, scores, and model inputs. Recorded software was Python 3.12.14, NumPy 2.3.5, pandas 2.2.3, SciPy 1.17.0, and matplotlib 3.10.8; the external workflow used GSEApy 1.3.1 and lifelines 0.30.3. The packages include retained requirements and software-version files and README instructions specifying analysis order, parameters, multiplicity families, and the limits of reproduction. Other modules are described at the reported method/result level, with extracted tables and retained figures where available; missing scripts, manifests, and matrices limit reconstruction. 

## 3. Results

### 3.1. Harmonized RNA Comparisons and Secondary Expression Observations

The primary eight-cancer RNA analysis used both paralogues from the same GDC STAR files, comprising 4365 primary tumors and 429 normal specimens. *WASHC2A* and *WASHC2C* were strongly correlated in primary tumors (Spearman rho 0.712–0.873; all BH-FDR ≤ 6.79 × 10^−78^; Table S1; Figure S1a,b). Both genes showed lower mean expression in BLCA, BRCA, COAD, LUAD, and LUSC, and higher mean expression in HNSC, LIHC, and PRAD (Table S2; Figure S2). Co-expression does not establish cross-mapping or paralogue specificity.

*WASHC2C* full-cohort distributional comparisons met FDR < 0.05 in seven cancers; PRAD showed a small positive mean ratio but no significant full-cohort difference (log2FC 0.111; FDR 0.403). In 52 matched PRAD pairs, the difference was significant (median paired log2(TPM + 1) difference 0.172; FDR 0.0177). In BLCA, the full-cohort *WASHC2C* FDR was 0.0475, but neither paralogue was significant in 19 matched pairs (*WASHC2A* FDR 0.180; *WASHC2C* FDR 0.441). Thus, inference depends on the sample design and the estimand (Tables S2 and S3).

**Figure 2:**
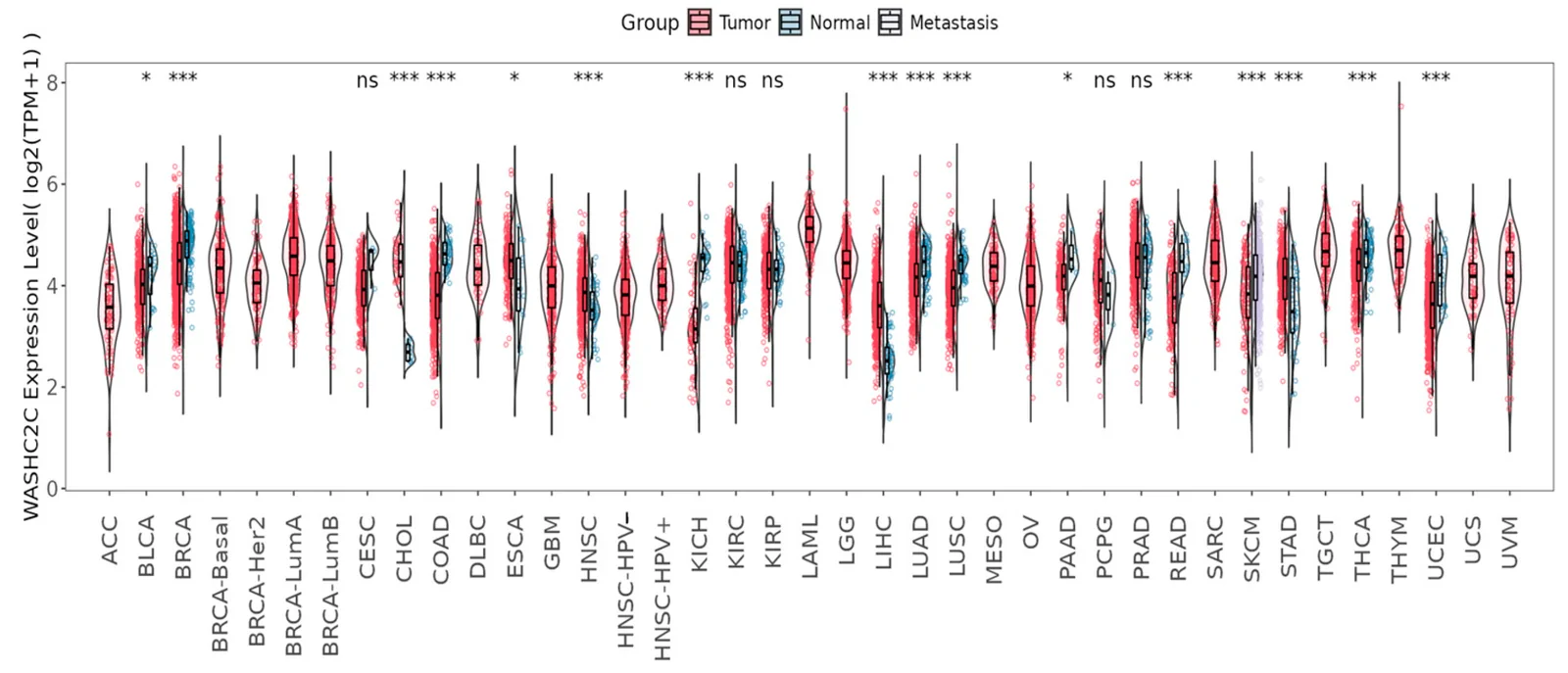
Secondary TIMER2.0 pan-cancer *WASHC2C* expression display. Violin plots show log2(TPM + 1) for the displayed TCGA tumor (red), normal (blue), and metastatic (gray) categories. Asterisks retain nominal Wilcoxon annotations (* p < 0.05, *** *p* < 0.001; ns, not significant).

**Figure 3:**
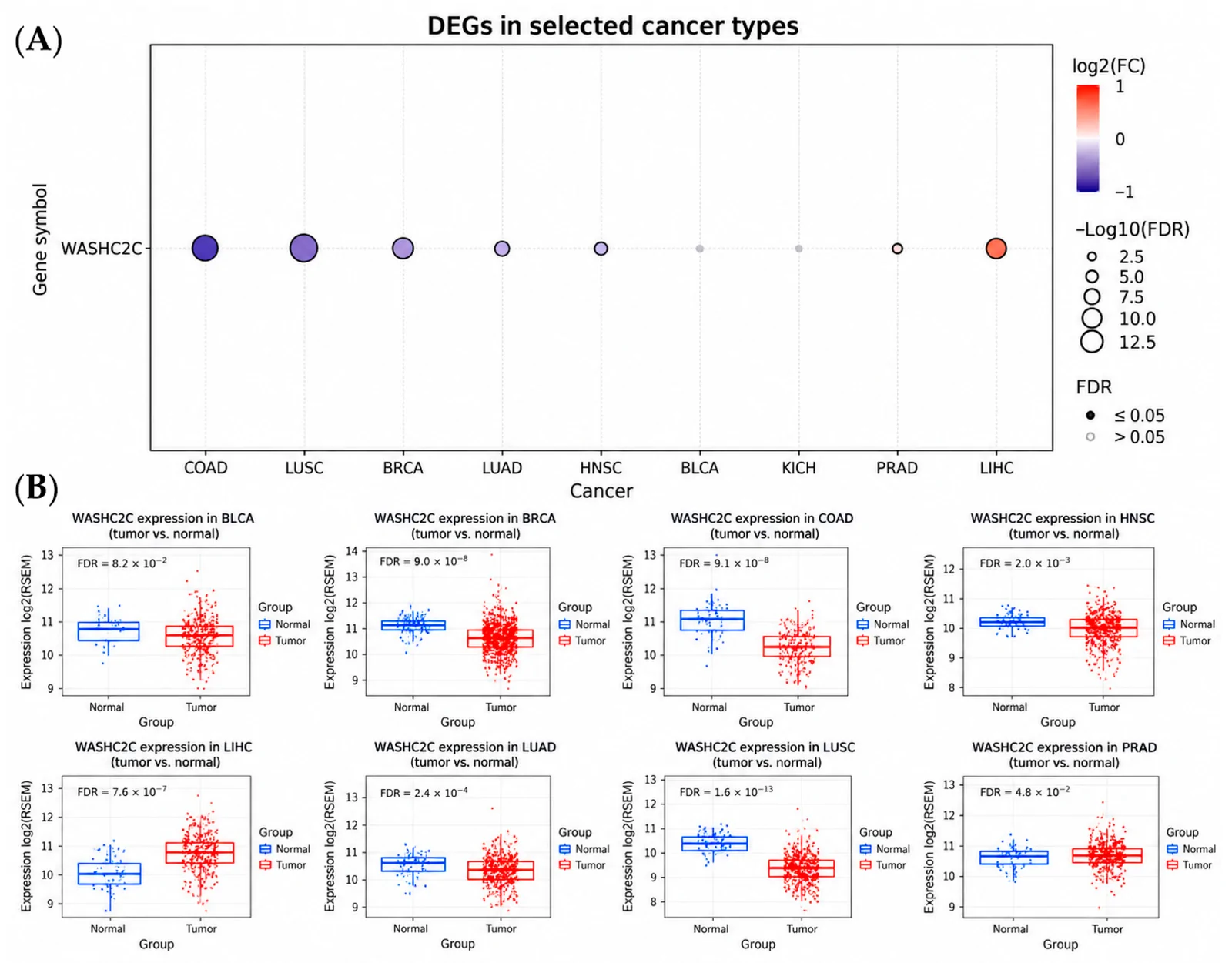
Secondary GSCA tumor–normal expression comparisons of *WASHC2C* across selected cancer types. (**A**) Bubble plot summarizing tumor-versus-normal differential expression of *WASHC2C*. Bubble color represents log2 fold change, with red and blue indicating increased and decreased tumor expression, respectively. Bubble size is proportional to −log10(FDR). Black outlines denote FDR ≤ 0.05, whereas gray outlines indicate FDR > 0.05. (**B**) Boxplots showing log2-transformed RSEM expression in normal tissues (blue) and primary tumors (red) for BLCA, BRCA, COAD, HNSC, LIHC, LUAD, LUSC, and PRAD. Individual points represent samples, center lines indicate medians, and boxes represent interquartile ranges. FDR-adjusted *p* values are displayed above each comparison.

Secondary analyses are shown in Figure 2 and Figure 3 do not supersede the harmonized STAR comparison. GSCA reported lower *WASHC2C* expression in LUSC (FDR 1.6 × 10^−13^), COAD (9.1 × 10^−8^), BRCA (9.0 × 10^−8^), LUAD (2.4 × 10^−4^), and HNSC (2.0 × 10^−3^). HNSC is explicitly discordant: GSCA indicates a decrease, whereas harmonized STAR TPM indicates an increase (log2FC 0.330; FDR 0.000335). Resource processing, sample selection, and expression estimands can differ; the available results do not establish the cause of this discordance.

GSCA also reported higher expression in LIHC (FDR 7.6 × 10^−7^) and PRAD (FDR 0.048), with nonsignificant BLCA (FDR 0.082) and KICH comparisons. KICH is therefore not described as significantly reduced. 

**Figure 4:**
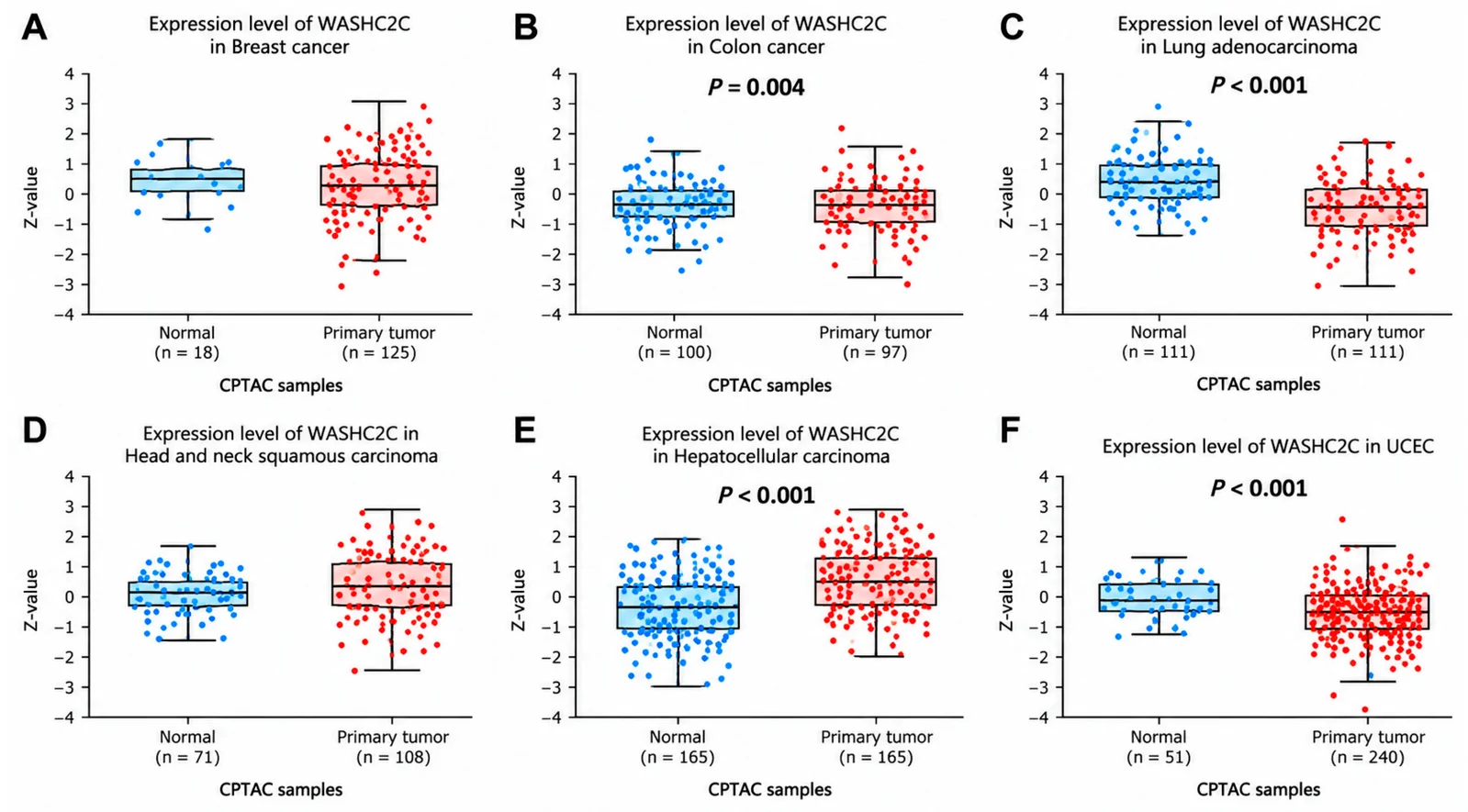
*WASHC2C* protein expression across normal and primary tumor tissues in CPTAC cohorts. *WASHC2C* expression (Z-values) was compared between normal and primary tumor samples in (**A**) breast cancer, (**B**) colon cancer, (**C**) lung adenocarcinoma, (**D**) head and neck squamous cell carcinoma, (**E**) hepatocellular carcinoma, and (**F**) uterine corpus endometrial carcinoma (UCEC). Blue and red points represent normal and primary tumor samples, respectively; sample sizes are indicated below each group. Significant differences were observed in colon cancer (*p* = 0.004), lung adenocarcinoma (*p* < 0.001), hepatocellular carcinoma (*p* < 0.001), and UCEC (*p* < 0.001); no significant difference was observed in breast cancer or head and neck squamous cell carcinoma.

**Figure 5:**
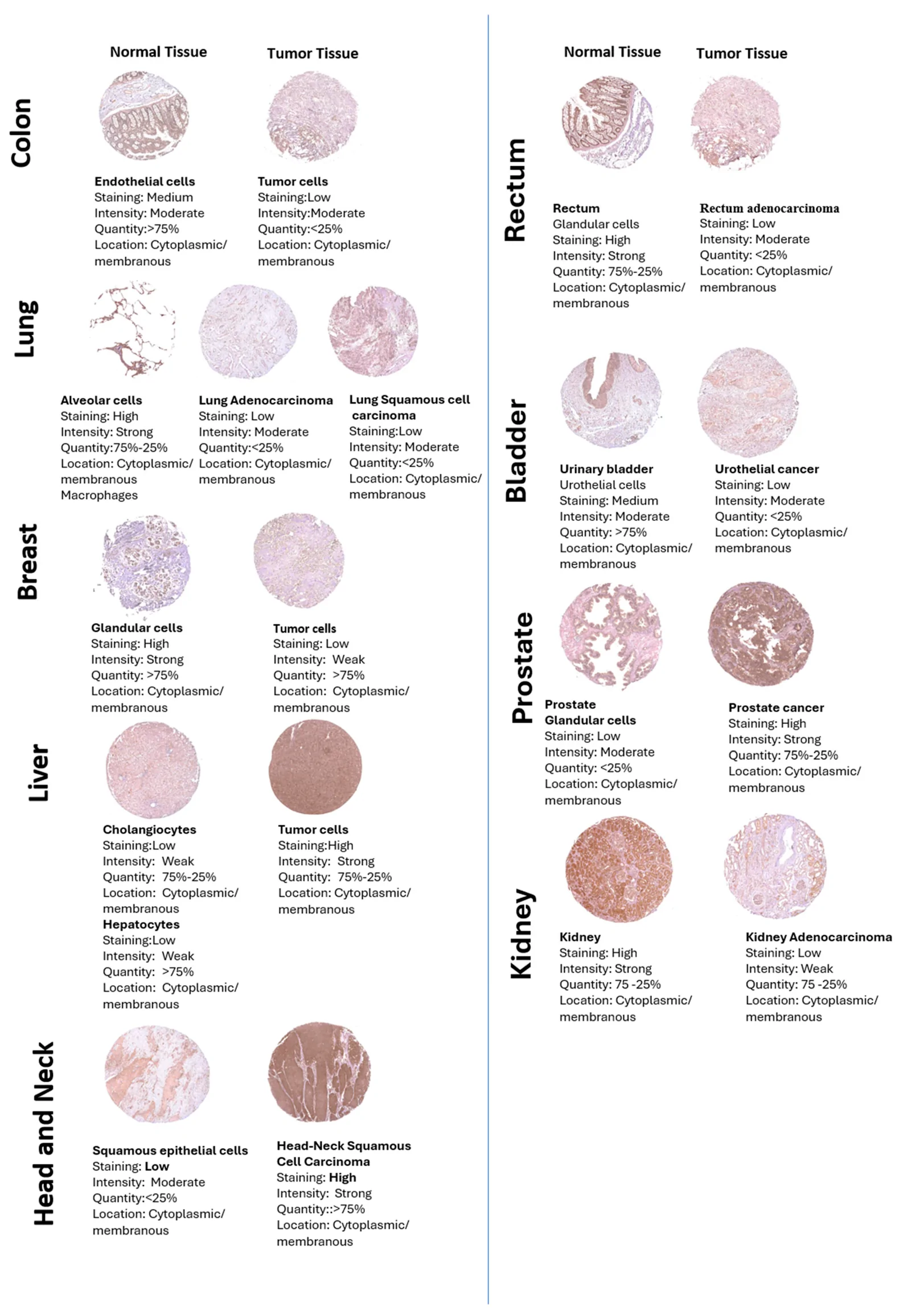
Illustrative HPA *WASHC2A*/*WASHC2C*-family immunoreactivity. Normal and tumor images represent colon, lung, breast, liver, head and neck, rectum, bladder, prostate, and kidney. Lung tumors include adenocarcinoma and squamous cell carcinoma; the kidney tumor is labeled kidney adenocarcinoma and is not assigned to KICH.

CPTAC observations showed lower abundance in COAD (nominal *p* = 0.004), LUAD and UCEC (both *p* < 0.001), and higher abundance in LIHC (*p* < 0.001; Figure 4). Figure 5 provides illustrative *WASHC2A*/*WASHC2C*-family immunoreactivity in the tissues actually displayed. The paralogue assessment quantified measurement uncertainty through sequence comparisons, antibody review, peptide evidence, and signature membership (Tables S5–S12).

The proportions of distinct *WASHC2C* transcript k-mers without an exact *WASHC2A* match were 51.43%, 59.49%, and 72.12% at 75, 100, and 150 bases. In the separate within-exon genomic analysis, 49.18%, 60.81%, and 83.17% had a single exact match in the GRCh38 primary assembly. These theoretical, read-length-dependent proportions are not observed in uniquely mapped patient-read fractions (Tables S6 and S7; Figure S3).

The reviewed HPA antibodies have 100% reported *WASHC2A* antigen identity, supporting only family-level immunoreactivity (Table S8). The locked signature comprises 50 genes, includes *WASHC2A*, and contains six genes overlapping 10q11 (Tables S10 and S11).

### 3.2. Unadjusted and Adjusted Survival Associations

**Figure 6:**
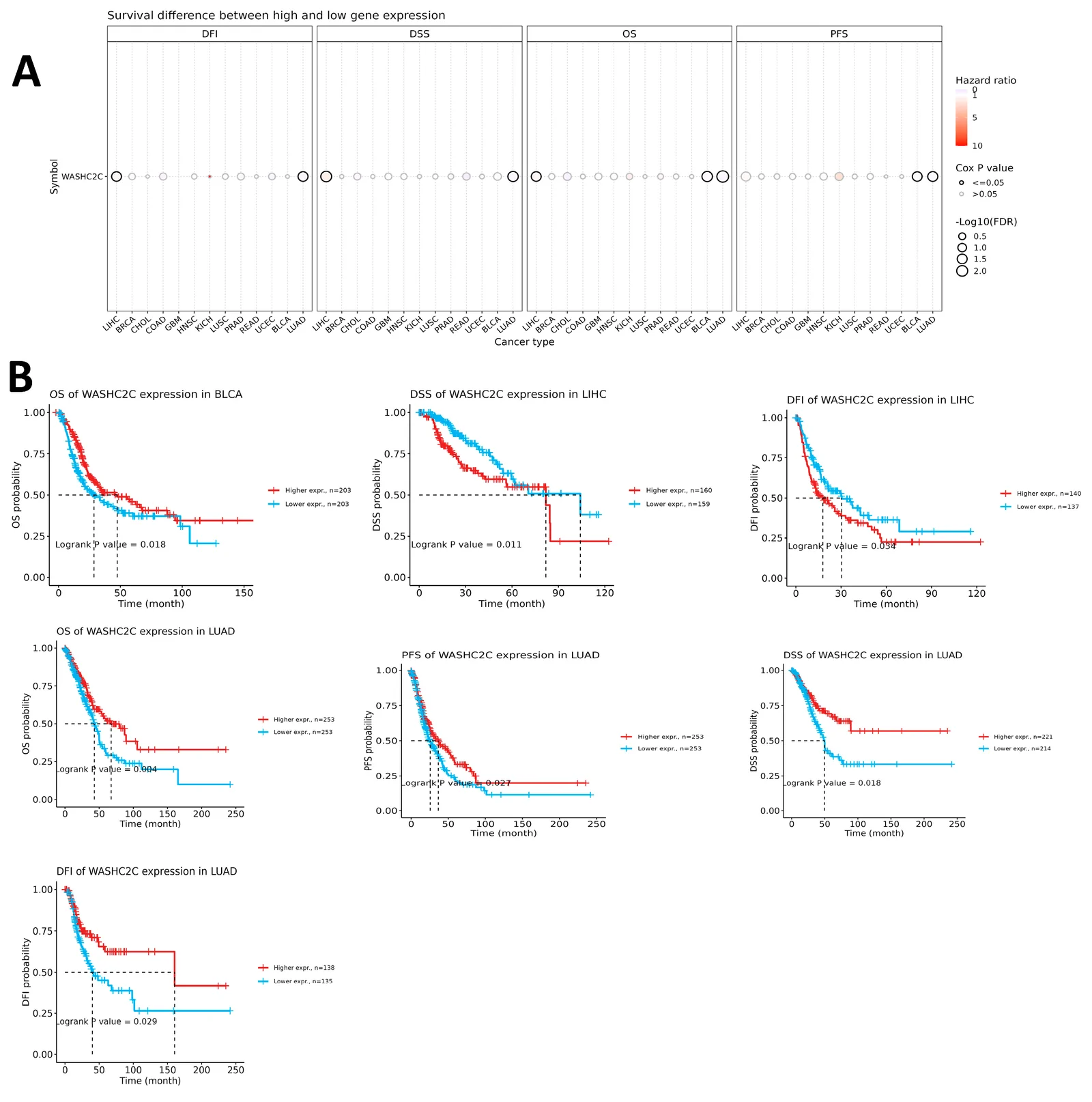
Exploratory unadjusted *WASHC2C* survival associations. (**A**) GSCA Cox bubble display for DFI, DSS, OS, and PFS. Color indicates HR, size indicates −log10(FDR), and outlines distinguish nominal Cox *p* ≤ 0.05 from *p* > 0.05. (**B**) Kaplan–Meier panels retain nominal log-rank comparisons in BLCA, LIHC, and LUAD. Red and blue indicate higher and lower expression; ticks indicate censoring.

The earlier GSCA univariate and Kaplan–Meier analyses suggested favorable *WASHC2C* associations in LUAD (OS nominal log-rank *p* = 0.004; PFS 0.027; DSS 0.018; DFI 0.029) and BLCA (OS *p* = 0.018). Figure 6 shows these unadjusted observations, which do not indicate that the associations persist after covariate adjustment or multiple-testing correction. Unadjusted adverse associations were observed for LIHC DSS (nominal *p* = 0.011) and DFI (*p* = 0.034). Their interpretation is superseded by the covariate-adjusted analysis below.

#### Multivariable Survival Analysis

In the primary model adjusted for age, sex, and pathological stage, LIHC OS (340 patients; 115 deaths) was the only *WASHC2C* association surviving BH correction (HR 1.612, 95% CI 1.177–2.208; *p* = 0.002914; FDR 0.034968). LIHC PFI (FDR 0.124134) and DSS (FDR 0.077913) were only nominally significant; the DSS gene-specific proportional-hazards test was significant (*p* = 0.007165). No LUAD endpoint or estimable BLCA endpoint met FDR < 0.05. Primary categorical-stage BLCA OS and DSS models were not estimable because stage I contained two patients with no OS/DSS events (Table S13).

These adjusted results modify the original favorable LUAD/BLCA interpretation. Stage-stratified BLCA sensitivity models were nonsignificant for OS (HR 1.055, 95% CI 0.793–1.403; *p* = 0.715317) and DSS (HR 1.191, 95% CI 0.838–1.692; *p* = 0.330232). Stage-stratified LIHC OS remained adverse (HR 1.573, 95% CI 1.145–2.162; *p* = 0.005225), but its separately adjusted FDR was 0.062698. No median-split association survived correction (Table S15). Thus, the primary LIHC OS finding shows model-specification dependence and does not establish an independent prognostic biomarker. Full coefficients, diagnostics, and sensitivity results are provided in Tables S13–S15 and Figure S4.

### 3.3. Association of WASHC2C Expression with Tumor Stage Across Cancer Types

**Figure 7:**
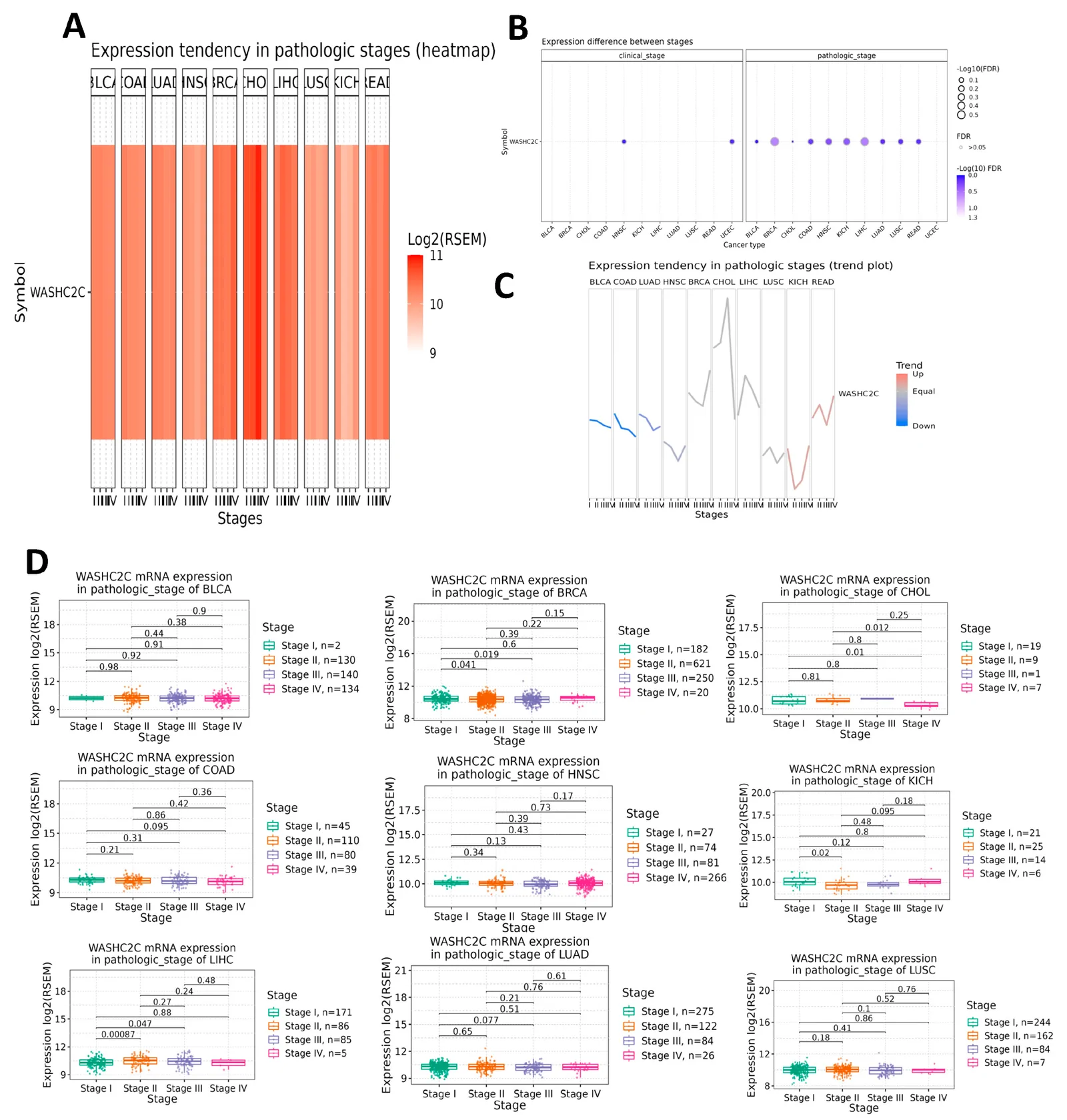
Association of *WASHC2C* expression with tumor stage across selected TCGA cancer types. (**A**) Heatmap showing the distribution of *WASHC2C* expression across pathological stages I–IV in BLCA, COAD, LUAD, HNSC, BRCA, CHOL, LIHC, LUSC, KICH, and READ. Expression levels are presented as log2-transformed RSEM values. (**B**) Bubble plots summarizing differences in *WASHC2C* expression among clinical and pathological stages. Bubble size and color represent −log10(FDR), while outlined bubbles indicate statistically significant stage-related differences. (**C**) Trend plot illustrating increasing, decreasing, or relatively unchanged *WASHC2C* expression across pathological stages in each cancer type. (**D**) Boxplots comparing *WASHC2C* expression among pathological stages I–IV in BLCA, BRCA, CHOL, COAD, HNSC, KICH, LIHC, LUAD, and LUSC. Sample sizes are shown in the legends, and pairwise *p* values are displayed above the corresponding comparisons.

Stage comparisons showed heterogeneous exploratory differences (Figure 7). Nominal pairwise *p* values were 0.041 and 0.019 for BRCA stage I versus II and III; 0.010 and 0.012 for CHOL stage IV versus I and II; 0.020 for KICH stage I versus II; and 8.7 × 10^−4^ and 0.047 for LIHC stage I versus II and III. The displayed BLCA, COAD, HNSC, LUAD, and LUSC pairwise comparisons did not meet the nominal *p* < 0.05 threshold. These values are not presented as adjusted pairwise significance, and sparse stage groups limit inference.

### 3.4. Association of WASHC2C Expression with Oncogenic Pathway Activity Across Cancer Types

**Figure 8:**
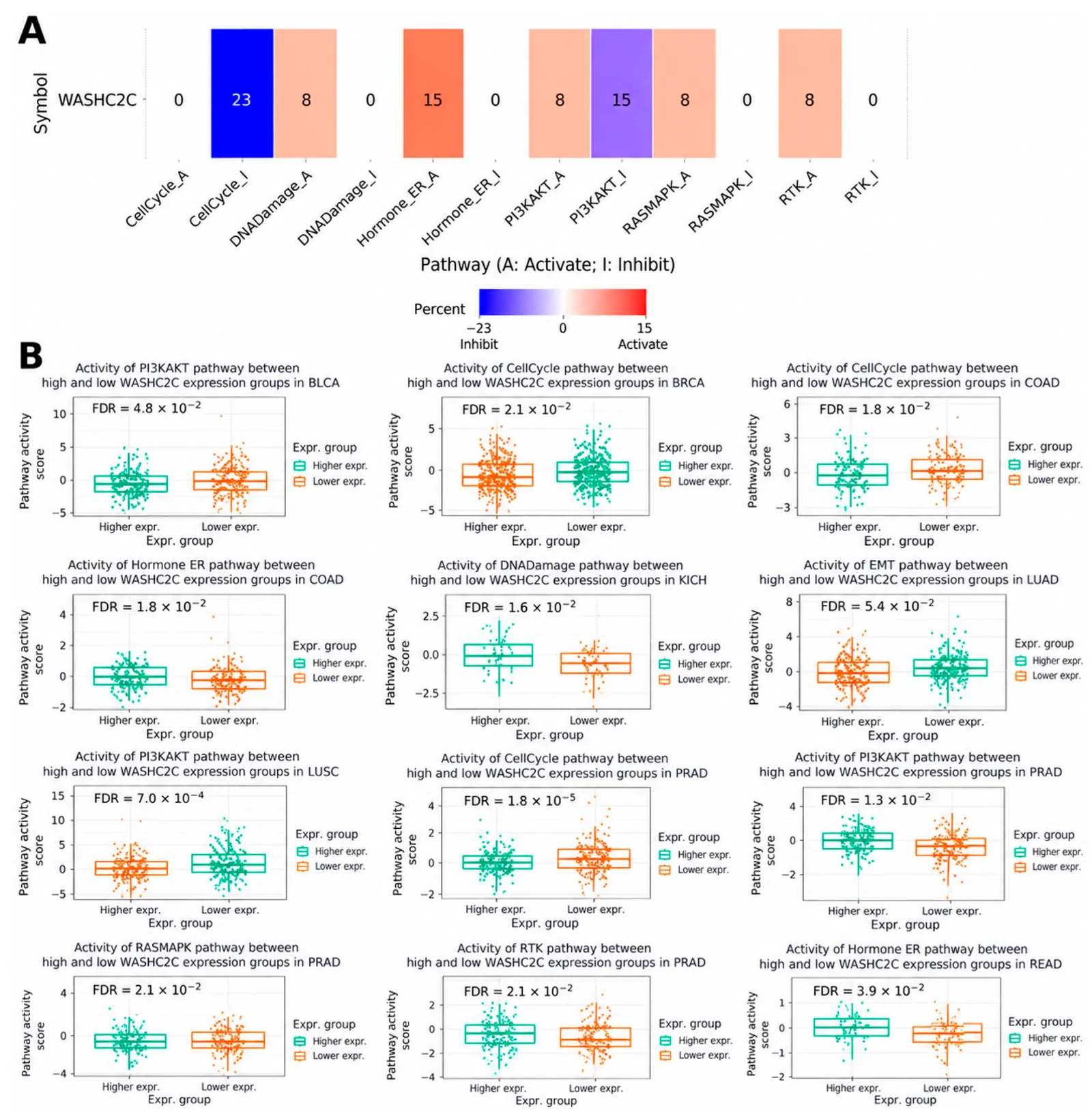
Association between *WASHC2C* expression and oncogenic pathway activity across selected cancer types. (**A**) Summary heatmap showing the percentage of cancer types in which *WASHC2C* expression was associated with higher (A) or lower (I) pathway scores of CellCycle, DNA Damage, Hormone ER, PI3K-AKT, RAS-MAPK, and RTK pathways. Red and blue indicate higher and lower scores, respectively; the source A/I labels do not establish causal activation or inhibition. (**B**) Boxplots comparing pathway activity scores between high- and low-*WASHC2C* expression groups in BLCA, BRCA, COAD, KICH, LUAD, LUSC, PRAD, and READ. Individual points represent tumor samples, center lines indicate medians, and boxes represent interquartile ranges. FDR-adjusted *p* values are displayed above each comparison.

To characterize pathway associations, pathway activity scores were compared between tumors with high and low *WASHC2C* expression across selected TCGA cohorts. Across the 13-cohort summary underlying Figure 8A, *WASHC2C* expression was associated with lower CellCycle scores in 3/13 cancers (BRCA, COAD and PRAD) and lower PI3K/AKT scores in 2/13 (BLCA and LUSC). Higher Hormone ER scores occurred in 2/13 (COAD and READ); higher DNA-damage scores in 1/13 (KICH); and higher PI3K/AKT, RAS/MAPK and RTK scores in 1/13 each (PRAD). The displayed 23%, 15%, and 8% represent rounded fractions of 13. Corresponding FDR values were 0.021, 0.018, and 0.000018 for CellCycle in BRCA, COAD, and PRAD; 0.048 and 0.00070 for PI3K/AKT in BLCA and LUSC; 0.018 and 0.039 for Hormone ER in COAD and READ; and 0.016 for DNA damage in KICH. PRAD PI3K/AKT, RAS/MAPK, and RTK FDR values were 0.013, 0.021, and 0.021. LUAD EMT did not meet FDR < 0.05 (FDR 0.054).

### 3.5. Cancer-Specific Enrichment and Prognostic Relevance of the WASHC2C-Associated GSVA Signature

To assess the functional and clinical relevance of *WASHC2C*, enrichment of the *WASHC2C*-associated gene set was evaluated across cancer types, and its associations with patient survival were assessed. The *WASHC2C*-associated gene set displayed heterogeneous enrichment patterns across cancers. Positive normalized enrichment scores were observed in LIHC and PRAD, whereas HNSC, KICH, BLCA, BRCA, LUAD, LUSC, and COAD showed negative enrichment. COAD and LUSC showed the strongest negative enrichment, while LIHC showed the most prominent positive enrichment.

Consistent with these patterns, tumor tissues generally exhibited lower GSVA scores than normal tissues in BLCA, BRCA, COAD, HNSC, KICH, LUAD, and LUSC. In contrast, LIHC tumors showed higher GSVA scores, while PRAD displayed comparatively modest tumor–normal differences.

**Figure 9:**
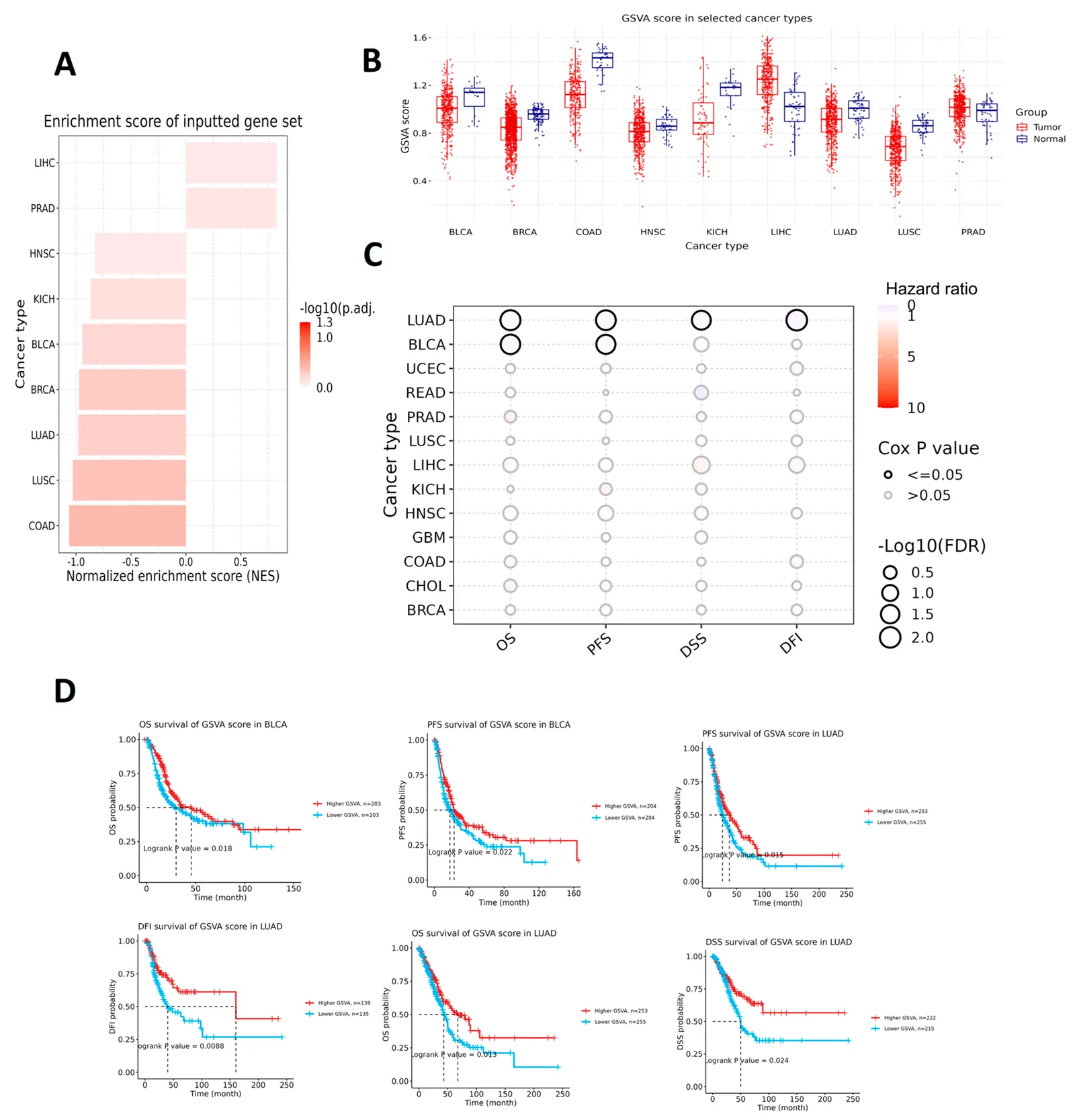
Cancer-specific enrichment, expression distribution, and prognostic relevance of the *WASHC2C*-associated GSVA signature. (**A**) Gene-set enrichment analysis showing normalized enrichment scores for the *WASHC2C*-associated gene set across selected cancer types. Positive and negative scores indicate relative enrichment and depletion, respectively, while color intensity represents −log10-adjusted *p* values. (**B**) Distribution of GSVA scores in tumor tissues (red) and normal tissues (blue). Boxes represent the interquartile range, center lines indicate medians, and individual points represent samples. (**C**) Bubble plot summarizing univariate Cox regression analyses of GSVA scores for OS, PFS, DSS, and DFI. Bubble color represents the hazard ratio, with values below 1 indicating a favorable association with a higher GSVA score and values above 1 indicating an adverse association. Bubble size corresponds to −log10(FDR); black outlines indicate Cox *p* ≤ 0.05, whereas gray outlines indicate *p* > 0.05. (**D**) Kaplan–Meier curves for the significant associations in BLCA and LUAD. Patients were stratified into high- and low-GSVA groups (red and blue, respectively). Survival differences were evaluated using the log-rank test; vertical marks indicate censored observations, and dashed lines indicate median survival. Panel A shows effect direction as the normalized enrichment score, and panel C shows it as the hazard ratio.

The TCGA signature analysis showed favorable nominal log-rank associations in LUAD for OS (*p* = 0.013), PFS (*p* = 0.015), DSS (*p* = 0.024), and DFI (*p* = 0.0088), and in BLCA for OS (*p* = 0.018) and PFS (*p* = 0.022). These exploratory multigene score associations are distinct from adjusted single-gene *WASHC2C* models. The selected curves do not establish clinical prognostic performance (Figure 9).

#### External Signature Assessment

In secondary GSE31210 analyses, 204 patients contributed 30 deaths and 54 relapse events. Higher scores were associated with favorable stage-adjusted OS (HR per SD 0.461, 95% CI 0.303–0.703; nominal *p* = 0.000315188) and relapse after adjustment for age, sex, stage, and smoking (HR 0.516, 95% CI 0.380–0.701; nominal *p* = 0.0000231297; Table S17). Median groups contained 102 patients each (Figure S5). Only 48/50 genes were measurable; neither *WASHC2C* nor *WASHC2A* contributed to this score. Stage II violated PH in the adjusted OS model; stage stratification retained the score association (HR 0.465, 95% CI 0.305–0.709; nominal *p* = 0.000369876). ssGSEA gave concordant GSE31210 findings. 

### 3.6. Cancer-Specific Associations Between the WASHC2C-Related GSVA Signature and Oncogenic Pathway Activity

To characterize the signaling context associated with *WASHC2C*, Spearman correlations were calculated between the *WASHC2C*-related GSVA score and the activities of major cancer-related pathways across selected TCGA cohorts. The correlation analysis revealed predominantly weak-to-moderate, cancer-specific associations between the *WASHC2C*-related GSVA score and pathway activity. The strongest positive association was observed between the GSVA score and DNADamage activity in KICH (Spearman *r*_s_ = 0.38; the displayed FDR is below 0.05). Positive associations were also detected in READ with RASMAPK activity (*r*_s_ = 0.33, FDR = 0.045); the Hormone ER association was borderline (*r*_s_ = 0.32; displayed FDR = 0.050, unrounded value unavailable). In PRAD, the GSVA score correlated positively with PI3KAKT (*r*_s_ = 0.17, FDR = 0.016), RASMAPK (*r*_s_ = 0.16, FDR = 0.026), and RTK (*r*_s_ = 0.15, FDR = 0.034) activities. A positive association with Hormone ER activity was additionally observed in COAD (*r*_s_ = 0.19, FDR = 0.023).

**Figure 10:**
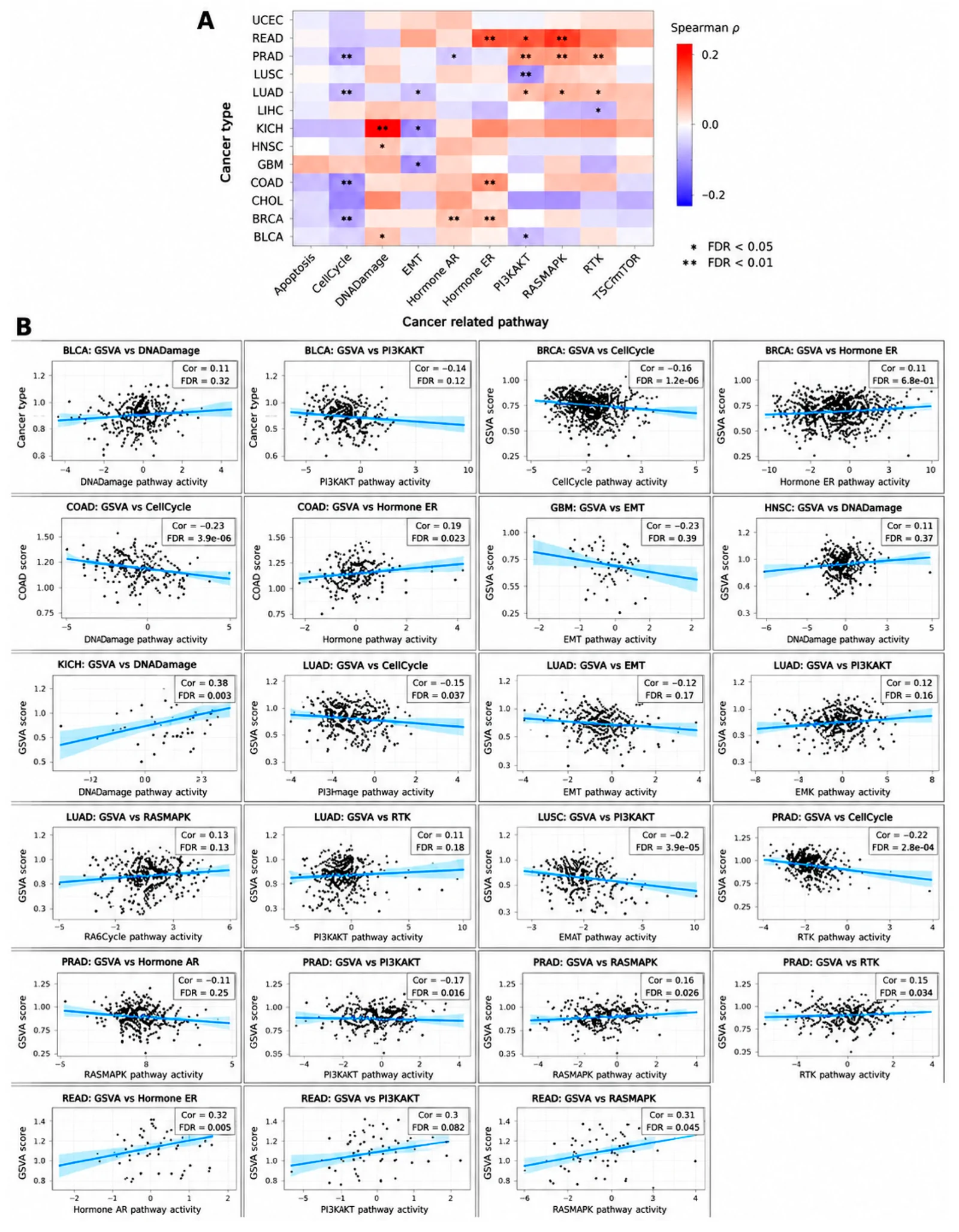
Pan-cancer associations between the *WASHC2C*-related GSVA score and cancer-related pathway activity. (**A**) Heatmap showing Spearman correlation coefficients between the *WASHC2C*-related GSVA score and the activities of Apoptosis, CellCycle, DNADamage, EMT, Hormone AR, Hormone ER, PI3KAKT, RASMAPK, RTK, and TSCmTOR pathways across selected cancer types. Red indicates positive correlations, blue indicates negative correlations, and lighter colors indicate coefficients closer to zero. Asterisks denote nominal statistical significance, whereas hash symbols indicate associations that remained significant after FDR correction. (**B**) Representative scatterplots illustrating associations between the GSVA score and selected pathway activity scores in BLCA, BRCA, COAD, GBM, HNSC, KICH, LUAD, LUSC, PRAD, and READ. Each point represents an individual tumor sample. Blue lines show fitted trends, and gray-shaded regions represent 95% confidence intervals. The insets report the Spearman correlation coefficient and the FDR-adjusted *p* value.* *p* < 0.05; ** *p* < 0.001.

The strongest negative associations involved CellCycle activity. The GSVA score was inversely correlated with CellCycle activity in COAD (*r*_s_ = −0.23, FDR = 3.9 × 10^−3^), PRAD (*r*_s_ = −0.22, FDR = 2.8 × 10^−4^), BRCA (*r*_s_ = −0.16; displayed FDR < 0.05), and LUAD (*r*_s_ = −0.15, FDR = 0.037). A significant inverse association with PI3KAKT activity was also detected in LUSC (*r*_s_ = −0.20, FDR = 3.9 × 10^−3^). Several additional correlations were nominally significant but did not remain significant after FDR correction. Collectively, these findings suggest that the pathway context associated with the *WASHC2C* signature differs substantially among cancer types (Figure 10A,B).

### 3.7. Pan-Cancer Genomic Alteration Landscape of WASHC2C

To characterize the genomic alteration profile of *WASHC2C*, its SNV frequency, variant spectrum, per-sample mutation burden, and copy-number alterations were examined across selected TCGA cohorts. *WASHC2C* exhibited a relatively low SNV frequency across most cancer types. The highest frequency was observed in UCEC, with 35 mutation-positive samples among 531 tumor samples (6.59%). These represented 35 altered patients among 530 unique patients; one wild-type patient, TCGA-BK-A139, contributed both a primary and a recurrent tumor sample. The UCEC sample-level denominator was retained for the mutation-frequency display. Frequencies in other cohorts included COAD (13 altered cases among 407 samples; approximately 3.2%) and BLCA (8 altered cases among 411 samples; approximately 2.0%). Mutation frequencies were lower in LUSC, LUAD, HNSC, PRAD, BRCA, READ, LIHC, and GBM, with the lowest frequency observed in GBM.

Missense mutations represented the predominant variant classification, followed by frameshift deletions, nonsense mutations, frameshift insertions, and splice-site alterations. SNPs were the most common variant type, whereas deletions and insertions were less frequent. Among the SNV classes, C>T substitutions were most abundant, followed by C>A substitutions; T>G substitutions were the least common. Most altered samples carried a single *WASHC2C* variant, with a median of one variant per sample and a maximum of five.

**Figure 11:**
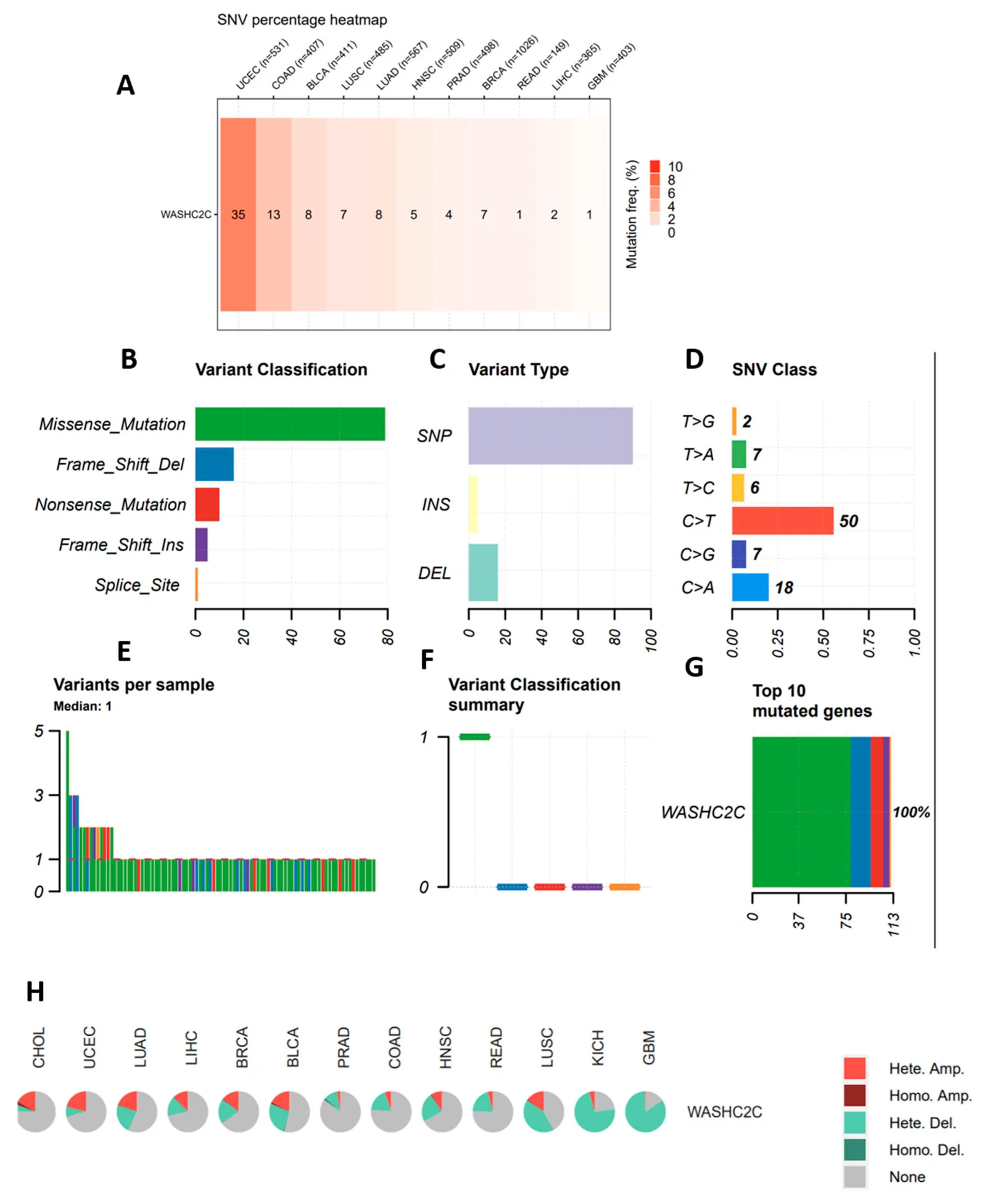
Pan-cancer genomic alteration landscape of *WASHC2C***.** The UCEC entry retains 35 mutation-positive samples among 531 tumor samples (6.59%), representing 35 altered patients among 530 unique patients. TCGA-BK-A139 contributes primary and recurrent wild-type samples. TCGA-AJ-A3BH remains in this mutation-frequency set but is excluded from PFS analysis because endpoint time is missing. (**A**) Heatmap showing the frequency of *WASHC2C* SNVs across selected cancer types. Columns represent cancer cohorts, with the total sample size indicated in parentheses. Numbers within cells indicate the number of samples carrying an SNV, whereas color intensity represents the corresponding mutation frequency. (**B**) Distribution of *WASHC2C* variants according to functional classification, including missense, frameshift deletion, nonsense, frameshift insertion, and splice-site variants. (**C**) Distribution of variant types, classified as SNPs, insertions, or deletions. (**D**) Spectrum of nucleotide substitutions, showing the numbers of C>A, C>G, C>T, T>A, T>C, and T>G events. (**E**) Number and classification of *WASHC2C* variants per altered sample; bar colors correspond to the variant classifications shown in panel B, and the median number of variants per sample is indicated. (**F**) Proportional summary of the detected variant classifications. (**G**) Gene-level mutation summary showing *WASHC2C* alterations among the 113 mutation-positive samples included in the gene-specific dataset; stacked colors represent the different variant classifications. (**H**) Distribution of *WASHC2C* copy-number states across selected cancer types. Red and dark red indicate heterozygous and homozygous amplification, respectively; light and dark green indicate heterozygous and homozygous deletion, respectively; gray indicates no copy-number alteration.

Copy-number analysis revealed heterogeneous patterns across cancers. Heterozygous deletions were particularly prominent in KICH and GBM and were also evident in LUSC, HNSC, COAD, and BLCA. Copy-number gains were comparatively infrequent but detectable in several cohorts, including CHOL, UCEC, LUAD, LIHC, BRCA, and LUSC. Nevertheless, most samples remained copy-number neutral across most cancer types (Figure 11A–H).

### 3.8. Prognostic Association of WASHC2C SNV Status Across Selected Cancers

To assess the clinical relevance of *WASHC2C* genomic variation, survival outcomes were compared between patients with *WASHC2C*-mutant and WT tumors across selected TCGA cohorts. The exploratory mutation-status analysis showed heterogeneous univariate hazard ratios across cancers and endpoints. Most displayed comparisons did not meet the nominal *p* threshold, and no protective effect of mutation was inferred.

**Figure 12:**
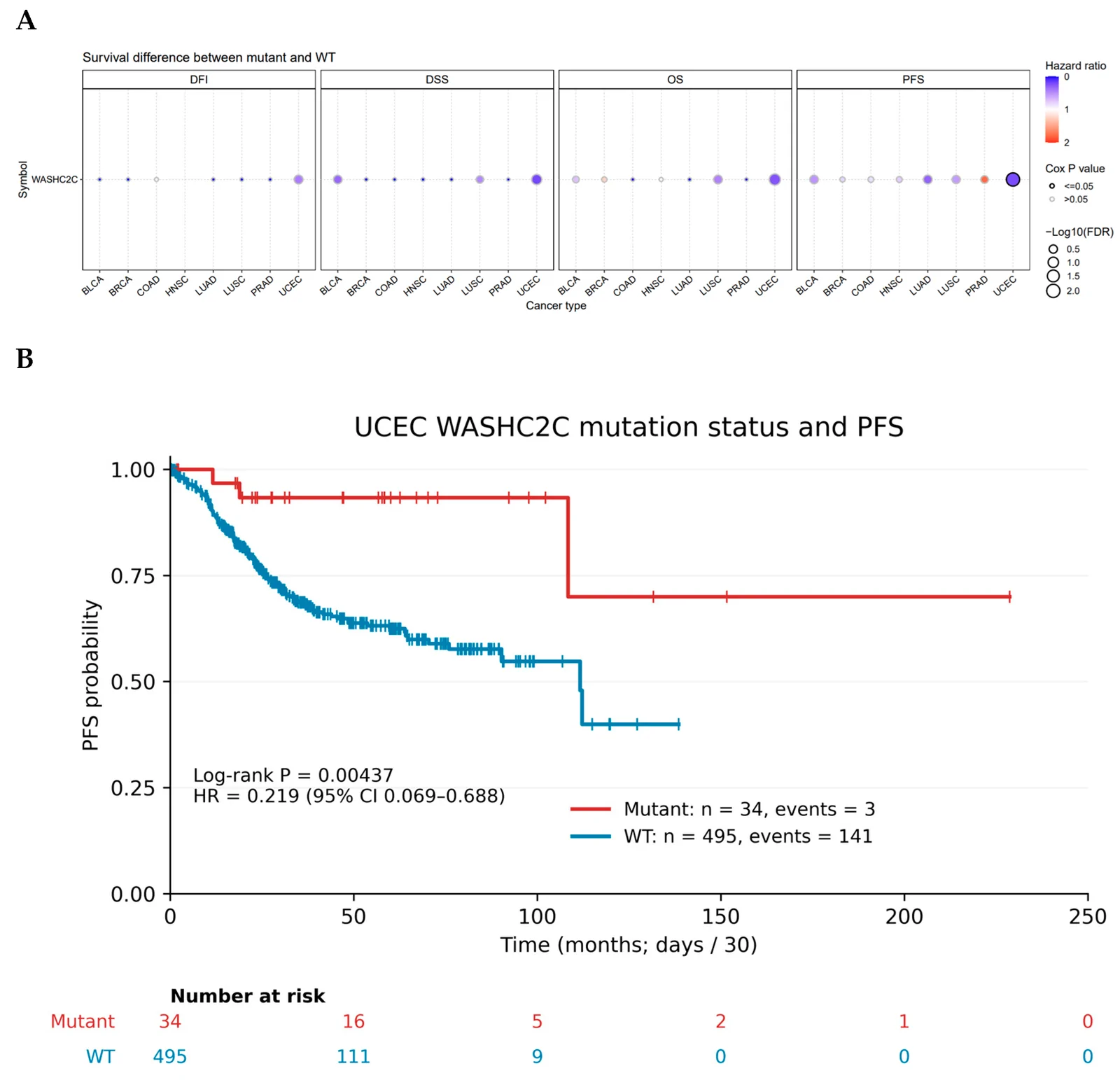
Exploratory *WASHC2C* mutation-status survival analyses. (**A**) Historical GSCA Cox summaries for DFI, DSS, OS, and PFS: color indicates HR, size indicates −log10(FDR), and outlines retain nominal Cox P thresholds. These are sample summaries; the UCEC PFS entry is superseded by the corrected patient-level analysis in panel B. (**B**) Reconciled UCEC PFS cohort comprising 529 unique patients: 34 mutant patients with 3 events and 495 wild-type patients with 141 events. TCGA-AJ-A3BH was excluded for missing PFS time, and the recurrent sample from TCGA-BK-A139 was removed to retain one primary tumor per patient. The nominal log-rank *p* value is 0.00437; the unadjusted Cox HR is 0.219 (95% CI 0.069–0.688; Efron ties). Tick marks indicate censoring, and the table shows numbers at risk. PFS uses the GSCA endpoint fields, which differ from TCGA-CDR PFI; time is in days/30. Only three mutant-group events support this exploratory association, which is not interpreted as protective or clinically informative.

The original UCEC PFS comparison was reproduced as 530 sample-level observations (34 mutant and 496 wild-type), comprising 529 unique patients. TCGA-AJ-A3BH, whose tumor carried an eligible *WASHC2C* frameshift deletion, was excluded because PFS time was missing. The remaining records counted the wild-type patient TCGA-BK-A139 twice through primary and recurrent tumor samples. After retaining one primary tumor per patient, the corrected cohort contained 529 patients (34 mutant and 495 wild-type), with 3 and 141 PFS events, respectively (144 total). The recalculated nominal log-rank *p* value was 0.00437; the unadjusted mutant-versus-wild-type HR was 0.219 (95% CI 0.069–0.688; Wald *p* = 0.00939). This exploratory association with PFS is based on only three mutant-group events and does not adjust for POLE, MSI/MMR, tumor mutational burden, or molecular subtype; it does not establish a protective, functional, or clinical effect (Figure 12B).

No consistent mutation-associated survival pattern was established across cohorts and endpoints. Figure 12A retains the historical summaries, whose UCEC entries remain sample-level exploratory results; Figure 12B presents the corrected patient-level UCEC PFS analysis. Nominal *p* values are distinguished from the FDR scale.

### 3.9. Pan-Cancer WASHC2C Copy-Number Alterations

**Figure 13:**
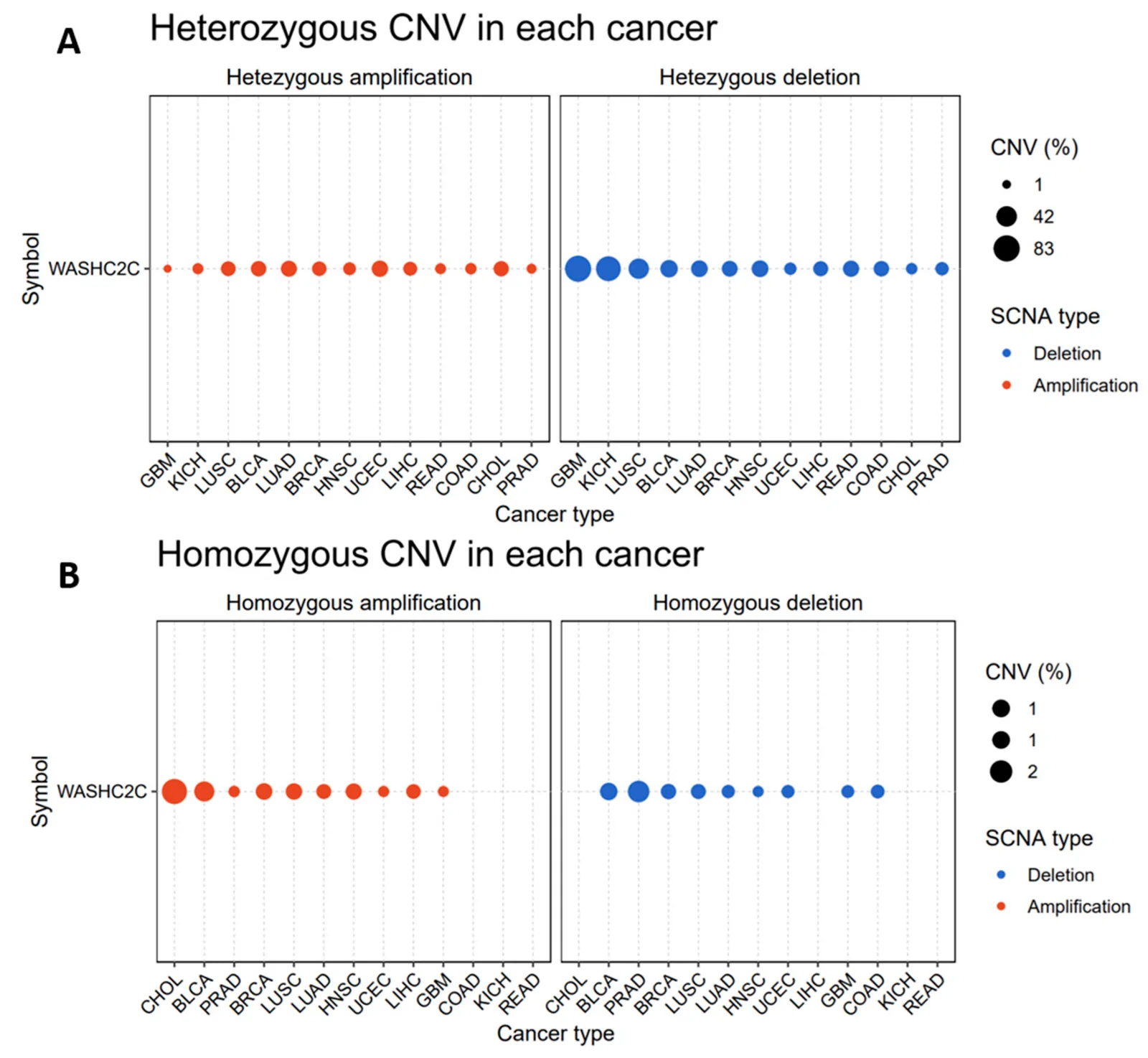
Pan-cancer distribution of *WASHC2C* copy-number alterations. (**A**) Frequencies of heterozygous amplification and deletion. (**B**) Frequencies of homozygous amplification and deletion. Bubble size represents CNV percentage; red and blue indicate amplification and deletion, respectively.

*WASHC2C* heterozygous and homozygous copy-number alterations were assessed across selected TCGA cancers. Heterozygous alterations were more frequent than homozygous events. Heterozygous deletions predominated, particularly in GBM and KICH, followed by LUSC and BLCA, whereas heterozygous amplifications occurred at lower frequencies across most cohorts. Homozygous alterations were rare, with amplification most evident in CHOL and BLCA and deletion most prominent in PRAD (Figure 13).

### 3.10. WASHC2C Copy-Number Variation Is Positively Associated with mRNA Expression Across Cancers

To assess whether genomic dosage contributes to *WASHC2C* transcriptional variation, Spearman correlations between *WASHC2C* CNV and mRNA expression were evaluated across selected TCGA cohorts. *WASHC2C* CNV showed positive correlations with mRNA expression in nearly all evaluated cancer types, suggesting that copy-number gain or loss may contribute to corresponding changes in transcript abundance. The strongest association was observed in KICH (*r*_s_ = 0.64, FDR = 2.3 × 10^−5^), followed by LUSC (*r*_s_ = 0.53, FDR = 1.2 × 10^−35^) and BLCA (*r*_s_ = 0.46, FDR = 2.6 × 10^−21^).

**Figure 14:**
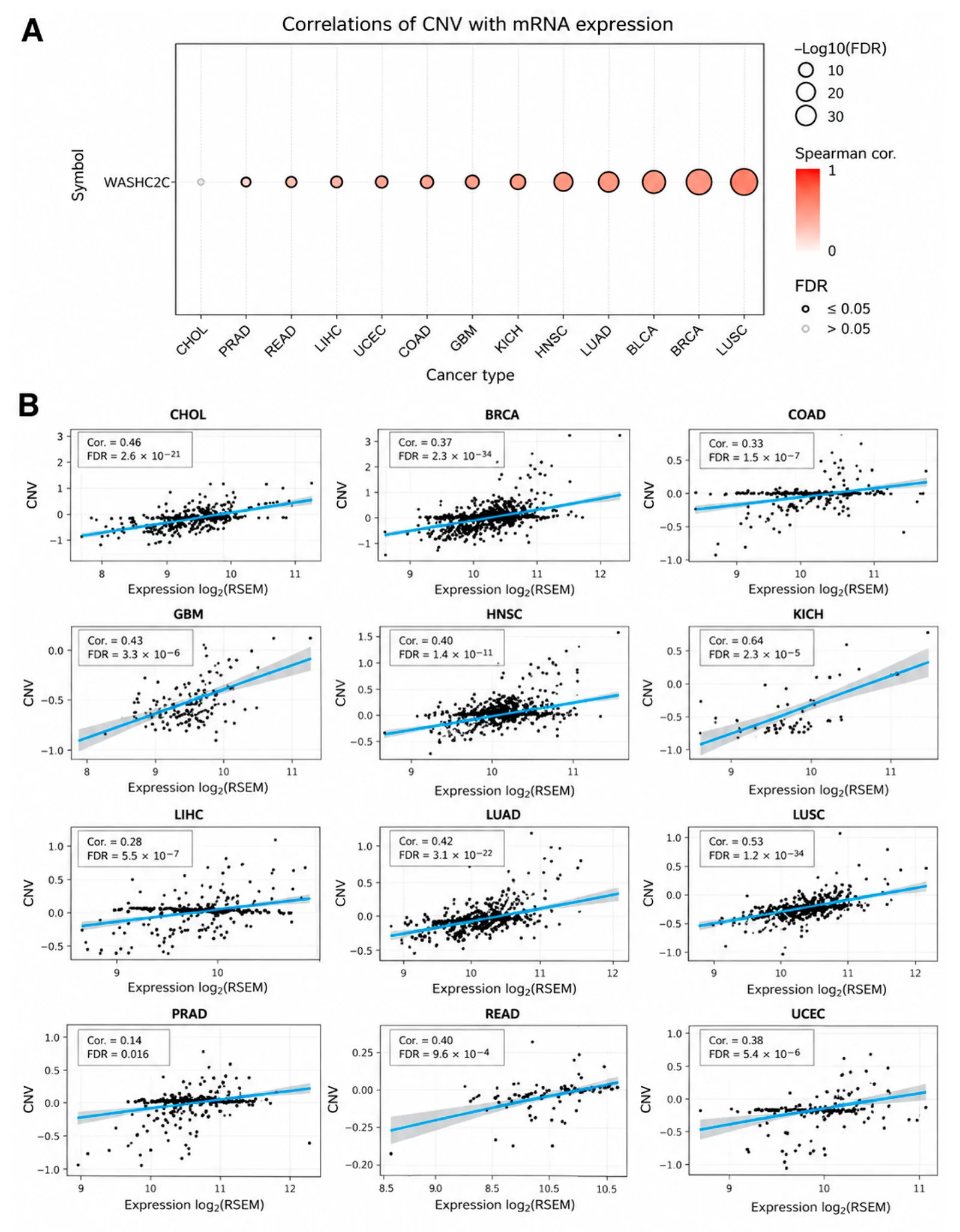
Correlation between *WASHC2C* copy-number variation and mRNA expression across selected cancer types. (**A**) Bubble plot summarizing Spearman correlations between *WASHC2C* CNV and mRNA expression. Bubble color intensity represents the Spearman correlation coefficient, with darker red indicating a stronger positive correlation. Bubble size is proportional to −log10(FDR). Black outlines indicate FDR ≤ 0.05, whereas gray outlines indicate FDR > 0.05. (**B**) Representative scatterplots showing the relationship between *WASHC2C* CNV and log2-transformed RSEM expression in BLCA, BRCA, COAD, GBM, HNSC, KICH, LIHC, LUAD, LUSC, PRAD, READ, and UCEC. Each point represents one tumor sample; blue lines show fitted trends, and gray shading indicates the 95% confidence interval. Insets report Spearman correlation coefficients and FDR-adjusted *p* values.

Moderate positive correlations were also detected in GBM (*r*_s_ = 0.43, FDR = 3.3 × 10^−6^), LUAD (*r*_s_ = 0.42, FDR = 3.1 × 10^−22^), HNSC (*r*_s_ = 0.40, FDR = 1.4 × 10^−19^), READ (*r*_s_ = 0.40, FDR = 9.6 × 10^−4^), UCEC (*r*_s_ = 0.38, FDR = 5.4 × 10^−6^), and BRCA (*r*_s_ = 0.37, FDR = 2.3 × 10^−34^). Weaker but significant associations were observed in COAD (*r*_s_ = 0.33, FDR = 1.5 × 10^−7^), LIHC (*r*_s_ = 0.28, FDR = 5.5 × 10^−7^), and PRAD (*r*_s_ = 0.14, FDR = 0.016). No significant inverse association was evident, while the association in CHOL appeared weak and nonsignificant. Collectively, these findings support a cancer-dependent positive relationship between *WASHC2C* copy number and transcript abundance without establishing a causal effect (Figure 14A,B).

### 3.11. Cancer-Specific Prognostic Associations of WASHC2C Copy-Number Alterations

**Figure 15:**
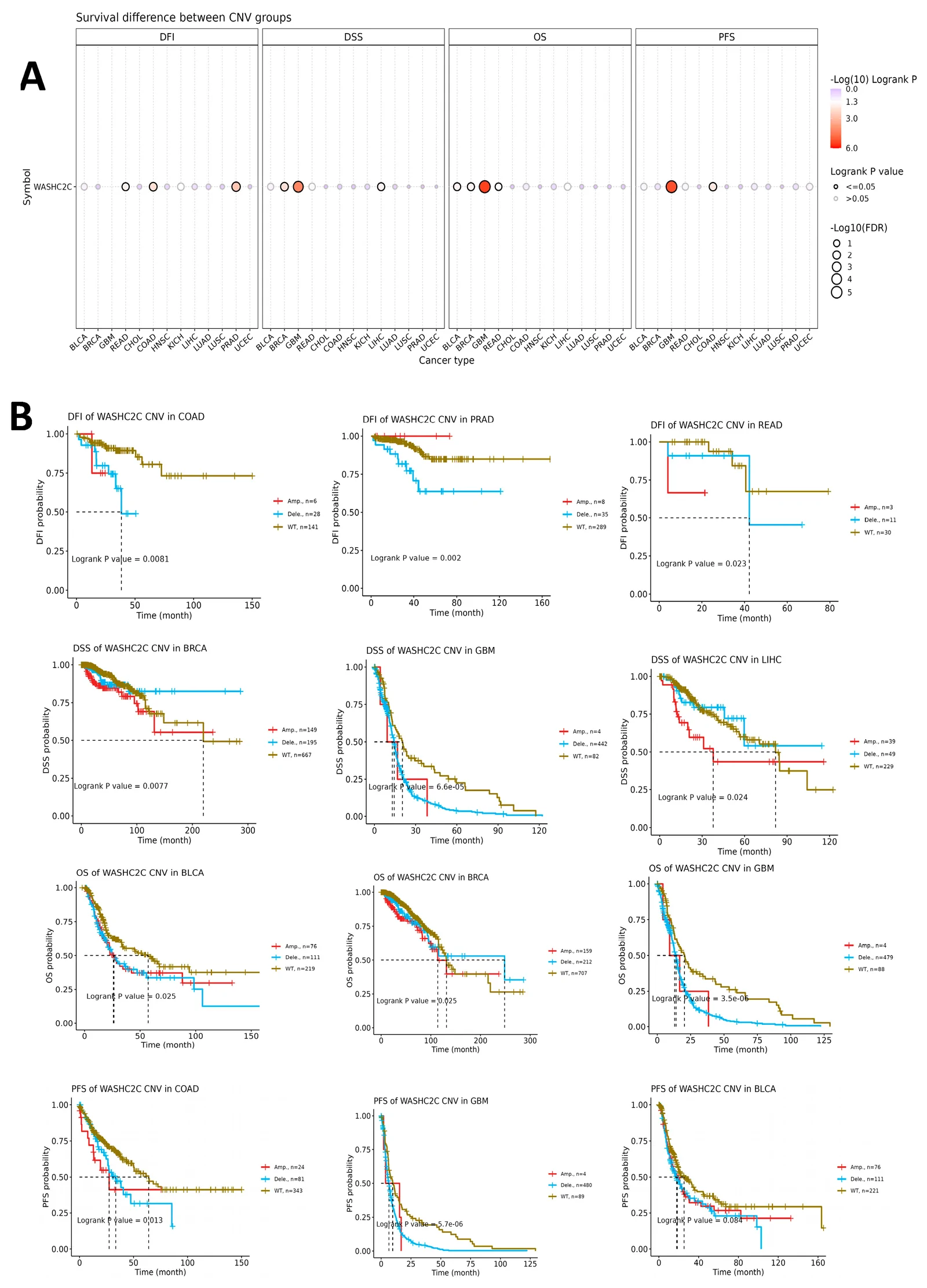
Exploratory survival comparisons by *WASHC2C* copy-number status. (**A**) Bubble summaries for amplification, deletion, and copy-number-neutral (WT) groups across DFI, DSS, OS, and PFS. Color represents −log10(nominal log-rank P), size represents −log10(FDR), and outlines retain nominal P thresholds. (**B**) Kaplan–Meier panels show amplification (red), deletion (blue), and WT (olive), with group sizes and nominal *p* values. Ticks indicate censoring; dashed lines indicate median survival where reached. Every comparison containing a CNV subgroup with n < 10 is considered unstable.

Copy-number survival associations varied by cancer type and endpoint. In GBM, nominal differences were observed for DSS (amplification n = 4, deletion n = 442, WT n = 82; nominal log-rank *p* = 6.6 × 10^−5^), OS (4/479/88; *p* = 3.5 × 10^−6^), and PFS (4/480/89; *p* = 5.7 × 10^−6^). Because the amplification group contained only four cases, these results were considered unstable and interpreted cautiously. In COAD, DFI differed among amplification, deletion, and WT groups (6/28/141; *p* = 0.0081), and PFS also differed nominally (24/81/343; *p* = 0.013). In PRAD, DFI differed among groups (8/35/289; *p* = 0.002), but the amplification subgroup was small. READ DFI also differed among groups (3/11/30; *p* = 0.023), with particularly limited numbers in the amplification subgroup. Nominal differences were additionally observed for BRCA DSS (149/195/667; *p* = 0.0077), BRCA OS (159/212/707; *p* = 0.025), LIHC DSS (39/49/229; *p* = 0.024), and BLCA OS (76/111/219; *p* = 0.025). BLCA PFS was not significant (76/111/221; *p* = 0.084). Overall, CNV-survival associations were heterogeneous, and results involving very small, altered groups are considered exploratory (Figure 15A,B).

### 3.12. Probe-Specific DNA Methylation Alterations in WASHC2C Across Cancer Types

**Figure 16:**
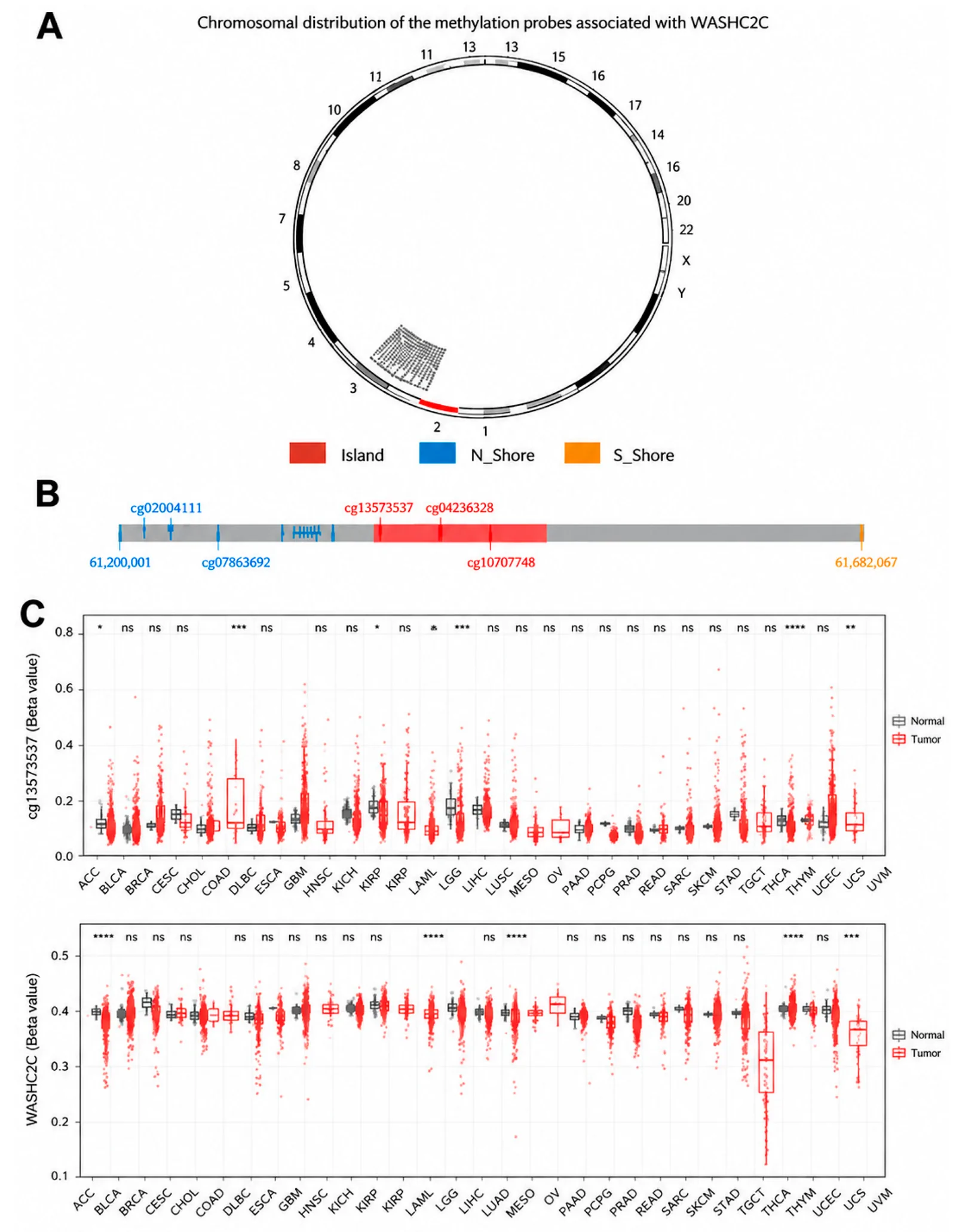
Pan-cancer DNA methylation landscape of *WASHC2C*. (**A**) Visualization of the chromosomal localization of methylation probes associated with *WASHC2C*. (**B**) Boxplots depicting differential *WASHC2C* methylation levels between tumor tissues (red) and corresponding normal tissues (gray) across a spectrum of cancer types, using the N-shore probe cg15344919. Beta values range from 0 (no methylation) to 1 (complete methylation). Nominal significance, assessed via the Wilcoxon test, is indicated by asterisks (* *p* < 0.05, ** *p* < 0.01, *** *p* < 0.001, **** *p* < 0.0001). (**C**) Gene-level CpG-aggregated methylation profiling of *WASHC2C* across human cancers generated using the SMART platform.

CpG locations and tumor–normal methylation distributions were examined through SMART. The annotated region included a CpG island and flanking shores. The selected probe- and gene-level aggregate measures different quantities; neither correlated directly with *WASHC2C* expression (Figure 16A).

At the N-shore CpG probe cg15344919 (Figure 16B), methylation levels were generally low, with β-values predominantly ranging from approximately 0.05 to 0.20. Tumor–normal differences were observed in BLCA, BRCA, HNSC, KIRC, KIRP, LIHC, PCPG, PRAD, THCA, and UCEC, with direction and magnitude varying by cancer type. UCEC showed increased tumor-associated methylation, whereas the renal cohorts KIRC and KIRP showed lower methylation in tumor tissues.

Figure 16C represents the CpG-aggregated *WASHC2C* methylation profile generated by the SMART platform rather than an individual CpG probe. In this aggregated analysis, TGCT showed the most pronounced tumor-associated hypomethylation, followed by UCEC, with smaller differences observed in additional cancer types. These findings indicate that *WASHC2C* methylation patterns are cancer-context-dependent and may differ between individual CpG sites and gene-level aggregated methylation measures (Figure 16A–C). No direct methylation–expression correlation or causal regulatory effect was tested.

### 3.13. Pan-Cancer Association of WASHC2C with Chemokine, Immune-Checkpoint, and Antigen-Presentation Genes

**Figure 17:**
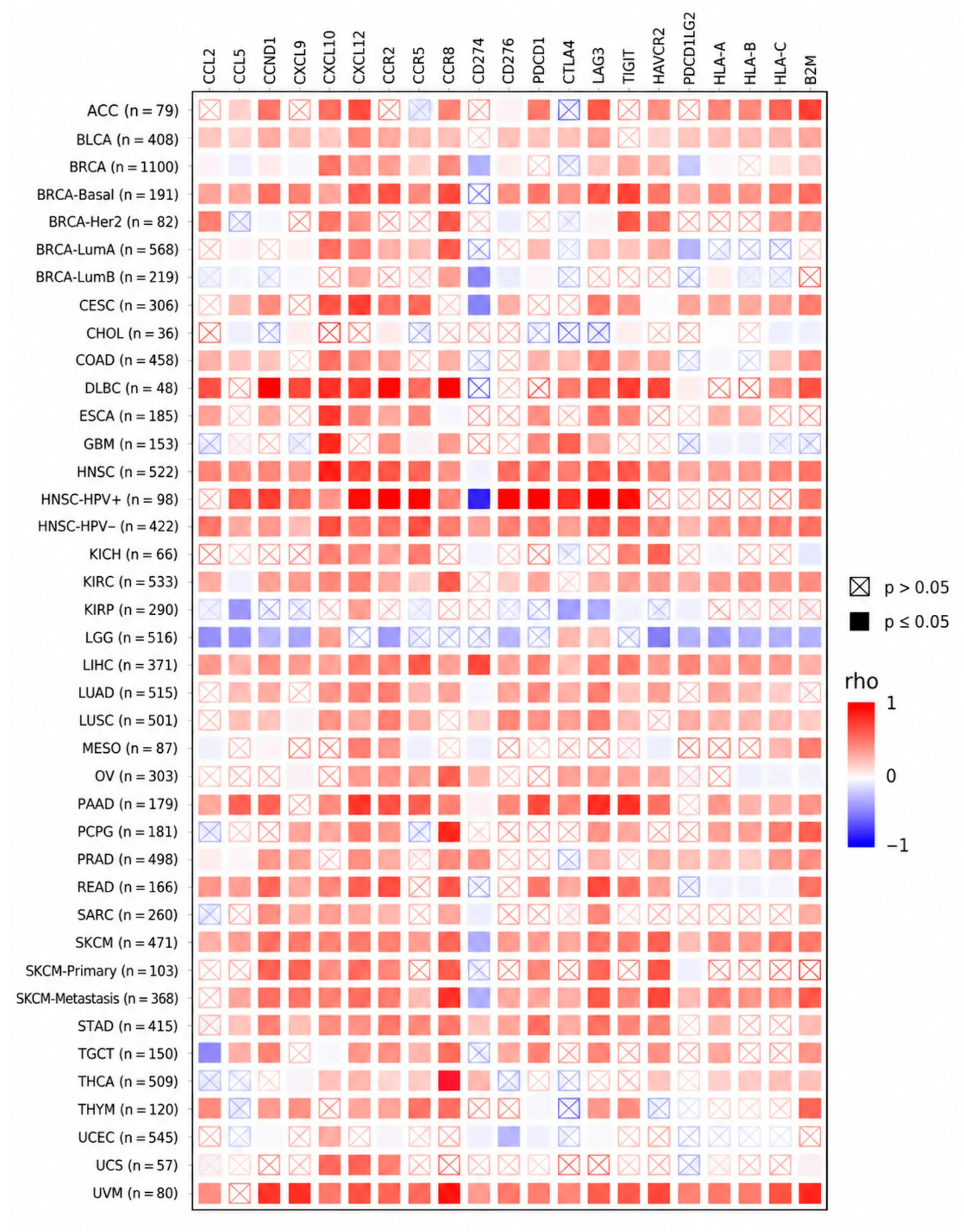
Pan-cancer correlations between *WASHC2C* expression and immune-regulatory genes. Heatmap showing Spearman correlation coefficients between *WASHC2C* expression and chemokines and chemokine receptors (CCL2, CCL5, CXCL9, CXCL10, CXCL12, CCR2, CCR5, and CXCR4), immune-checkpoint molecules (CD274, CD276, PDCD1, CTLA4, LAG3, TIGIT, HAVCR2, and PDCD1LG2), and antigen-presentation-related genes (HLA-A, HLA-B, HLA-C, and B2M) across TCGA cohorts. Red and blue indicate positive and negative Spearman correlation coefficients (ρ), respectively, with increasing color intensity representing stronger correlations; white indicates coefficients close to zero. Crossed cells denote nonsignificant correlations (*p* > 0.05), whereas uncrossed cells indicate *p* ≤ 0.05.

To characterize the immunoregulatory context of *WASHC2C*, Spearman correlation analysis was performed between *WASHC2C* expression and selected chemokines, chemokine receptors, immune-checkpoint genes, and antigen-presentation molecules across TCGA cohorts. *WASHC2C* expression showed predominantly positive associations with immune-related genes, although the magnitude and direction varied across cancer types. The most extensive positive correlation pattern was observed in UVM, where *WASHC2C* was positively associated with nearly all evaluated chemokine, checkpoint, and antigen-presentation genes. Broad positive associations were also evident in HNSC-HPV+, HNSC-HPV−, DLBC, LIHC, PAAD, and several BRCA subgroups, particularly for CCL2, CXCL9, CXCL10, CCR2, CCR5, CD274, CTLA4, LAG3, TIGIT, HAVCR2, and PDCD1LG2. Positive correlations with HLA-A, HLA-B, HLA-C, and B2M further suggested an association between *WASHC2C* expression and antigen-presentation activity in selected cohorts (Figure 17).

In contrast, LGG displayed the most prominent inverse correlation profile, including negative associations with several chemokines, immune-checkpoint genes, HLA molecules, and B2M. Additional cancer-specific negative associations were observed in KIRP and selected BRCA, CESC, SKCM, and UCEC cohorts, particularly for CD276 and some checkpoint-related genes. Crossed cells indicated that several visually apparent correlations did not reach the nominal P threshold. Overall, the findings suggest that *WASHC2C* is linked to distinct immune-regulatory transcriptional patterns that depend on the cancer context, without implying a direct regulatory relationship.

### 3.14. WASHC2C Expression Is Associated with Distinct Immune and Stromal Infiltration Patterns Across Cancers

To characterize the immune and stromal context of *WASHC2C*, purity-adjusted Spearman correlation analyses were performed between *WASHC2C* expression and infiltration estimates for T-cell subsets, B cells, macrophages, NK cells, and cancer-associated fibroblasts across TCGA cohorts. All *p* values in this subsection are nominal. Pan-cancer analysis revealed heterogeneous yet predominantly positive associations between *WASHC2C* expression and immune cell infiltration. Among the representative findings, the strongest positive CD8^+^ T-cell association was observed in PRAD using EPIC (ρ = 0.434, P = 1.57 × 10^−20^), followed by READ using CIBERSORT-ABS (ρ = 0.402, P = 8.59 × 10^−5^). CD4^+^ T-cell infiltration showed its strongest positive association in COAD using TIMER (ρ = 0.485, P = 1.21 × 10^−17^), whereas an inverse association with central-memory CD4^+^ T cells was detected in KICH using xCell (ρ = −0.392, P = 1.23 × 10^−3^).

Positive B-cell associations were particularly evident in HNSC (ρ = 0.432, P = 8.29 × 10^−24^), KICH (ρ = 0.411, P = 6.69 × 10^−4^), and LIHC (ρ = 0.405, P = 4.46 × 10^−15^). Macrophage-related infiltration was also positively associated with *WASHC2C* expression in several cohorts, including READ (ρ = 0.422, P = 3.47 × 10^−5^), HNSC (ρ = 0.405, P = 8.55 × 10^−21^), and LIHC (ρ = 0.400, P = 1.03 × 10^−14^). In contrast, a negative association with M1 macrophage infiltration was observed in UCEC (ρ = −0.259, P = 1.48 × 10^−2^).

**Figure 18:**
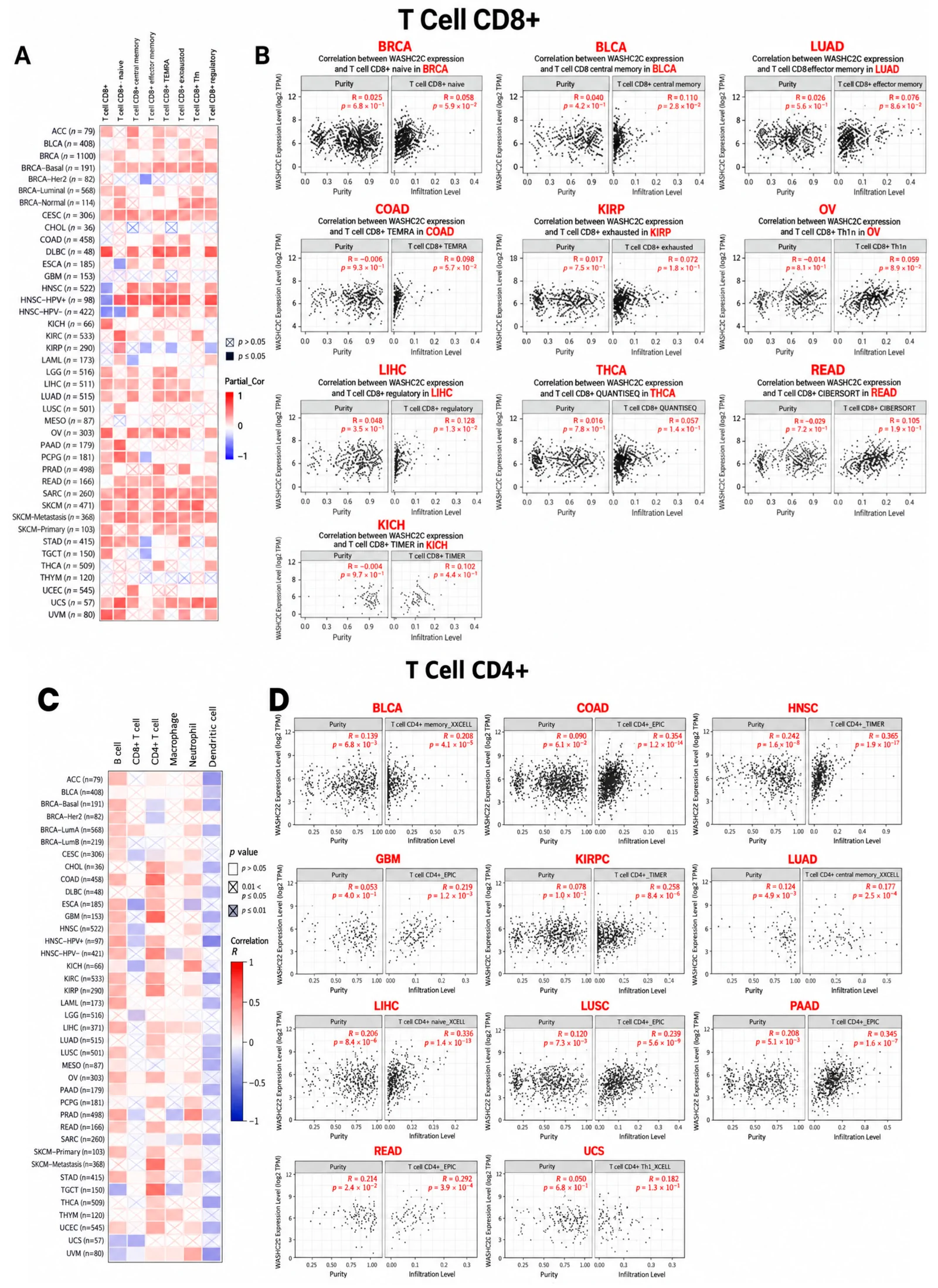

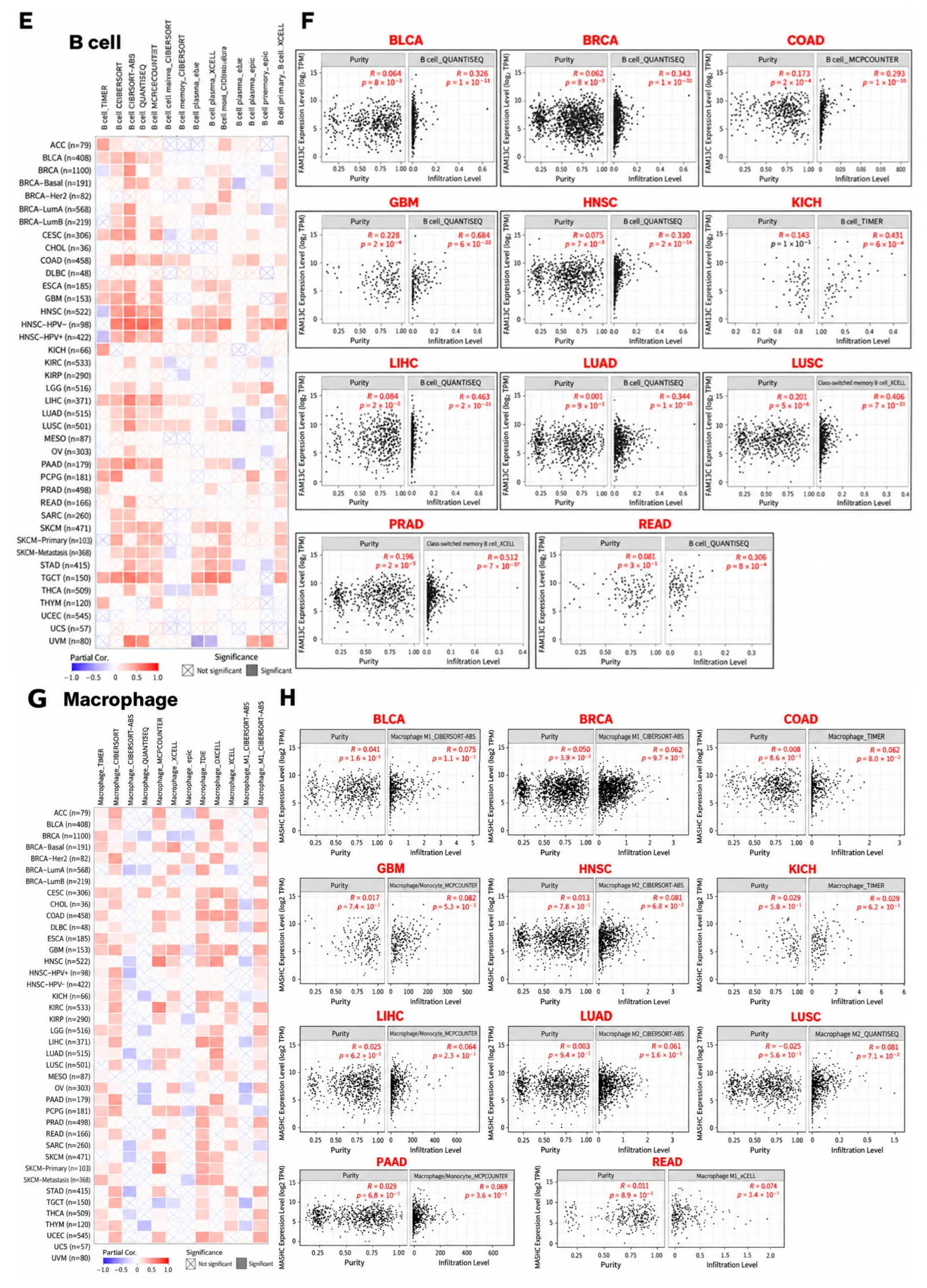

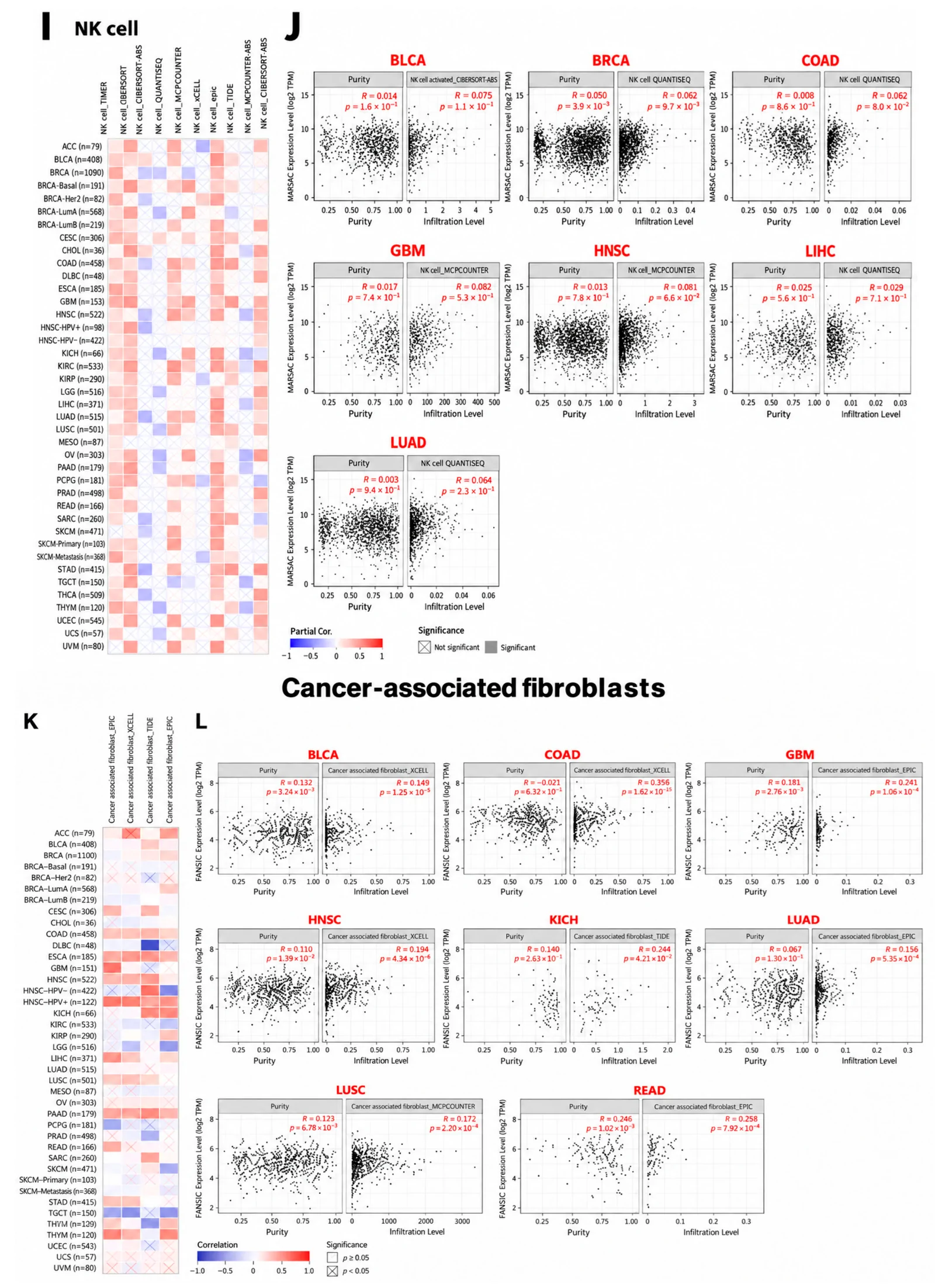
Immune and stromal cell associations of *WASHC2C* across human cancers. Panels (**A**–**L**) present six heatmap–scatterplot pairs examining associations between *WASHC2C* expression and infiltration by CD8+ T cells (**A**,**B**), CD4+ T cells (**C**,**D**), B cells (**E**,**F**), macrophages/monocytes (**G**,**H**), natural killer (NK) cells (**I**,**J**), and cancer-associated fibroblasts (**K**,**L**). The heatmaps (**A**,**C**,**E**,**G**,**I**,**K**) summarize tumor purity-adjusted Spearman correlations across cancer types, with red and blue indicating positive and negative correlations, respectively. The corresponding scatterplot panels (**B**,**D**,**F**,**H**,**J**,**L**) illustrate selected cancer-specific associations. For each cancer type, the left scatterplot shows the relationship between *WASHC2C* expression and tumor purity, whereas the right scatterplot shows the relationship between *WASHC2C* expression and estimated immune or stromal cell infiltration. Each point represents an individual tumor sample, and correlation coefficients (R) andp-values are indicated. Cell infiltration estimates were obtained using TIMER, EPIC, MCP-counter, quanTIseq, CIBERSORT, CIBERSORT-ABS, xCell, and TIDE, where available. Crosshatched heatmap cells indicate nonsignificant associations (*p* ≤ 0.05).

For NK cells, the strongest positive correlations were detected in HNSC using MCP-counter (ρ = 0.372, P = 1.34 × 10^−17^) and GBM (ρ = 0.338, P = 5.36 × 10^−5^). Cancer-associated fibroblast infiltration was positively related to *WASHC2C* expression in multiple cancers, with the most prominent associations observed in GBM using EPIC (ρ = 0.325, P = 1.07 × 10^−4^), HNSC using xCell (ρ = 0.274, P = 6.85 × 10^−10^), and LIHC using EPIC (ρ = 0.270, P = 3.50 × 10^−7^). Collectively, these findings suggest that *WASHC2C* is associated with cancer-specific immune and stromal infiltration patterns, although the direction and magnitude of these relationships vary by cell subset, cancer type, and deconvolution method (Figure 18).

### 3.15. Exploratory Mouse Washc2 Treatment Response Observations

In TISMO syngeneic mouse models, Washc2 expression differed across selected response groups, with heterogeneous model-, treatment-, and time-dependent patterns (Figure S6). These observations provide exploratory biological context and do not validate human WASHC2C cancer biology or predict clinical immunotherapy response.

### 3.16. Single-Cell Associations Between WASHC2C Expression and Cancer Functional States

**Figure 19:**
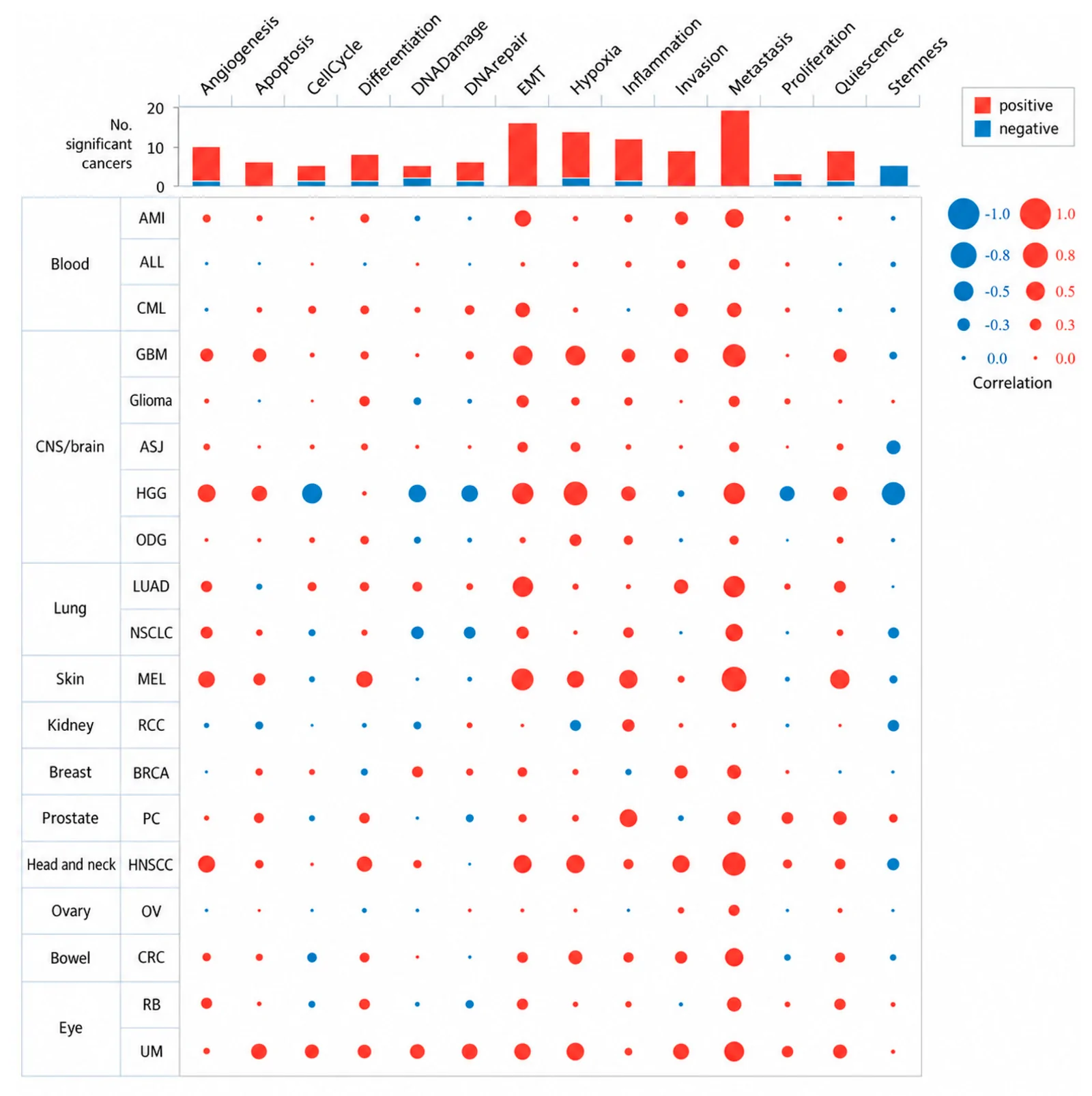
Single-cell association of *WASHC2C* expression with cancer functional states. The upper bar chart shows the number of datasets with significant positive (red) or negative (blue) correlations. The bubble plot summarizes correlations across cancer datasets, with bubble color indicating direction and size representing correlation strength. *WASHC2C* was most frequently positively associated with metastasis, EMT, hypoxia, inflammation, invasion, and quiescence, whereas negative associations were less common.

To investigate the potential functional relevance of *WASHC2C* at single-cell resolution, its expression was correlated with 14 cancer-related cellular states across multiple single-cell datasets. *WASHC2C* expression showed heterogeneous, cancer-dependent associations with cellular functional states. Positive correlations predominated, with metastasis showing the most significant associations across datasets, followed by EMT, hypoxia, inflammation, invasion, and quiescence. Strong positive relationships with metastasis were particularly evident in GBM, HGG, LUAD, MEL, HNSCC, and UM, while EMT and hypoxia also showed prominent positive correlations in GBM, HGG, LUAD, and MEL (Figure 19).

Negative associations were less frequent and were concentrated on selected datasets. HGG showed the most pronounced inverse pattern, including strong negative correlations with cell cycle, DNA damage, DNA repair, proliferation, and stemness. Negative associations with stemness were also observed in ASJ, NSCLC, RCC, and HNSCC. Overall, the findings suggest that elevated *WASHC2C* expression is preferentially associated with invasive, metastatic, and stress-adaptation states in several cancers, although the direction and magnitude of the relationships vary by dataset and do not establish causality (Figure 19).

## 4. Discussion

This study provides an integrated pan-cancer, multi-omics characterization of *WASHC2C*, combining transcriptomic and proteomic evidence with clinicopathological associations, survival analyses, pathway activity, gene-set enrichment, genomic and epigenetic alterations, immune profiles, immunotherapy-related findings, and single-cell functional states. The findings do not support a consistent oncogenic or tumor-suppressive role for *WASHC2C.* Instead, they suggest that its biological and clinical relevance depends heavily on tumor context, with variation in expression, prognostic value, molecular associations, signaling patterns, and immune interactions across cancer types. Collectively, these results extend the limited gene-specific knowledge of *WASHC2C* and underscore its potential relevance within the broader WASH-complex network that regulates actin dynamics, endosomal organization, and intracellular trafficking.

The harmonized primary analysis showed reduced mean *WASHC2C* expression in BLCA, BRCA, COAD, LUAD, and LUSC, whereas HNSC, LIHC, and PRAD showed higher expression. In PRAD, statistical significance varied depending on whether we analyzed the full cohort or matched tumor–normal pairs, while paired BLCA comparisons did not reach significance. Moreover, the expression direction observed for HNSC differed from that reported by the GSCA. These discrepancies underscore the need to distinguish clearly between analytical resources, cohort structures, and statistical estimands rather than interpreting the findings as evidence of uniform cross-platform consistency. Although the broader WASH regulatory machinery provides relevant biological context for these transcriptomic associations [16,17,18,19,20,21,22,23,24], the present data do not support a universal oncogenic or tumor-suppressive role for *WASHC2C* across cancer types.

The increased expression and adverse clinical associations observed in LIHC are consistent with direct experimental evidence showing that *FAM21C*/*WASHC2C* promotes hepatocellular carcinoma invasion and metastasis through actin remodeling and inhibition of CAPZA1-mediated capping [27]. In contrast, reduced expression in several other cancers may reflect distinct trafficking requirements or differences in the cellular source of the measured transcript. Findings involving *WASHC1*, retromer-associated proteins, or the wider WASH complex provide useful mechanistic context but should not be interpreted as direct evidence for *WASHC2C* [23,25,26,29].

CPTAC data and HPA images provide complementary protein-level context. However, the HPA antibodies reviewed show 100% sequence identity with *WASHC2A*, meaning that the observed staining should be interpreted as *WASHC2A*/*WASHC2C*-family immunoreactivity rather than as evidence of *WASHC2C*-specific protein expression. Accordingly, apparent agreement between RNA and protein patterns cannot be taken as direct validation of *WASHC2C* protein abundance. Cross-platform comparisons are further constrained by differences in assay specificity, tissue composition, and sample-processing procedures [8,9,10,11,14,15].

The paired-gene analysis demonstrates strong *WASHC2A*-*WASHC2C* co-expression and concordant tumor–normal directionality, while theoretical sequence analysis indicates that a substantial fraction of *WASHC2C* sequences can be distinguished from *WASHC2A* under exact-match conditions. Neither result establishes the observed fraction of uniquely mapped patient reads. Overall, the available data support *WASHC2C*-associated transcriptomic findings with explicit paralogue-related limitations.

Tumor–normal expression differences do not establish diagnostic utility. Stage-related observations were heterogeneous and retained nominal pairwise annotations. Independent assay validation, classification analyses, and prospective studies are needed to establish diagnostic or staging performance.

The covariate-adjusted STAR/CDR analysis did not support the favorable unadjusted observations in LUAD and BLCA. Because expression processing and complete-case cohorts also differed, we cannot attribute attenuation solely to adjustment. Primary BLCA OS/DSS models were non-estimable; stage-stratified sensitivity models were nonsignificant. LIHC OS showed an adverse association in the primary categorical-stage model (340 patients; 115 deaths; HR 1.612, 95% CI 1.177–2.208; FDR = 0.034968). Stage stratification retained a similar adverse estimate but gave FDR = 0.062698, and no median-split test survived correction. LIHC DSS also violated gene-specific PH. The direction is compatible with prior experimental findings [27], but statistical support depends on model specification and does not establish independently validated prognosis.

External evidence from the locked signature, including the secondary analysis of GSE31210, showed favorable survival associations for 48 genes that could be reliably measured. *WASHC2C* was not included in the original signature, whereas *WASHC2A* could not be evaluated in the survival cohorts because it was not reliably measurable under our conservative probe-mapping criteria. Variation in cohort characteristics, platform coverage, and score distributions may partly explain differences across datasets, although we did not examine these factors experimentally. Interpretation of the GSE31210 results is also limited by its restriction to stage I–II disease, the small number of deaths (n = 30), limited adjustment of the overall survival model, and evidence of proportional hazards violation. Accordingly, these findings should be viewed as supportive, cohort-specific evidence rather than validation of either WASHC2 paralogue, the complete 50-gene signature, or its clinical utility.

Pathway associations provide context for further investigation. Higher *WASHC2C* expression was associated with lower CellCycle scores in BRCA, COAD, and PRAD, and lower PI3K/AKT scores in BLCA and LUSC. PRAD also showed positive associations with PI3K/AKT, RAS/MAPK, and RTK scores, illustrating that these patterns are not mutually exclusive. The WASH scaffold and trafficking literature suggests possible biological connections [20,21,22,23,24], but the present comparisons do not establish direct pathway activation, inhibition, or mediation of survival outcomes.

The parallel expression and GSVA patterns suggest a broader co-expression program but do not establish *WASHC2C*-dependent regulation. The locked 50-gene signature contains *WASHC2A* and six genes overlapping 10q11; regional co-regulation and shared cellular context are possible contributors. Its source table reports Pearson coefficients, while the exact historical generation settings remain unverified. GSVA summarizes multiple genes and is distinct from *WASHC2C* expression [42,43]. Favorable unadjusted TCGA signature associations should therefore not be conflated with the nonsignificant adjusted single-gene findings in LUAD and BLCA.

Correlations between the GSVA signature and pathway activity supported the expression-stratified analyses. Recurrent inverse relationships with CellCycle activity in BRCA, COAD, LUAD, and PRAD suggest that reduced proliferative signaling is a feature of the *WASHC2C*-associated program in several cancers. Positive relationships with DNA-damage activity in KICH, Hormone ER signaling in COAD, and a borderline association in READ, and PI3KAKT, RASMAPK, and RTK signaling in PRAD further demonstrate lineage-specific network organization. The generally weak-to-moderate correlations are compatible with the multifactorial nature of a multigene signature but should not be interpreted as evidence of direct pathway regulation.

*WASHC2C* somatic variants were uncommon, and variant class alone does not establish functional consequences. Patient-level reconciliation identified a duplicate wild-type primary/recurrent sample pair and one mutant patient lacking PFS time. The corrected UCEC analysis included 529 unique patients, with only 3 PFS events among 34 mutant patients and 141 events among 495 wild-type patients. The exploratory association with PFS (unadjusted HR 0.219, 95% CI 0.069–0.688) remains imprecise and was not adjusted for POLE, MSI/MMR, tumor mutational burden, or molecular subtype. GSCA PFS includes additional death events relative to TCGA-CDR PFI and should retain its source-specific definition. These findings do not establish a protective or clinically useful effect.

Copy-number alterations appeared more closely related to *WASHC2C* transcription. Heterozygous deletions were more frequent than homozygous events, particularly in GBM and KICH, whereas high-level amplification was uncommon. Positive correlations between copy number and mRNA abundance across most cancers, especially KICH, LUSC, and BLCA, support a possible gene-dosage contribution. Nevertheless, copy-number segments contain multiple neighboring genes, and these associations do not establish *WASHC2C* as the selected target. Heterogeneous survival associations among amplification, deletion, and copy-number-neutral groups may reflect lineage-specific dosage effects, co-altered genes, treatment differences, and small altered subgroups. Locus-specific functional validation is therefore required.

Probe-specific and aggregated methylation patterns differed, including in UCEC. The selected representative N-shore probe cg15344919 lacks an established mechanistic selection rationale. No methylation–expression correlation was evaluated, so tumor–normal methylation differences cannot be assumed to regulate *WASHC2C* transcription [34].

The immune analyses demonstrated broad but tumor-specific relationships between *WASHC2C* and immune-checkpoint genes, chemokines, chemokine receptors, and antigen-presentation molecules. Positive co-expression with CD274, PDCD1, CTLA4, LAG3, TIGIT, HAVCR2, CCL5, CXCL9, CXCL10, and HLA-related genes in selected cancers may indicate an inflamed microenvironment accompanied by compensatory immunosuppression. Negative associations in other malignancies may instead reflect immune-excluded or lineage-specific states. These relationships are biologically plausible because actin remodeling and endosomal trafficking contribute to immune-cell migration, receptor recycling, antigen processing, and immune-synapse organization [30,35,36,37,38,39]. However, these functions have largely been established for immune-cell actin systems or the broader FAM21/WASH machinery and cannot be assigned directly to tumor-cell *WASHC2C*.

Infiltration patterns varied by cancer, cell subset, and algorithm. Apparent directional agreement is descriptive; a formal cross-algorithm consistency statistic was not reported. Tumor purity, reference signatures, and cellular composition remain possible confounders [40], and bulk *WASHC2C* expression may partly originate from infiltrating cells.

The TISMO observations concern mouse Washc2 in syngeneic tumor models [41], rather than human *WASHC2C i*n treated patients. Their model-, treatment-, and time-dependent heterogeneity supports only exploratory preclinical interpretation. No universal response pattern or clinically predictive biomarker has been established.

Single-cell analyses provided additional resolution by linking *WASHC2C* to metastasis, EMT, hypoxia, inflammation, invasion, and quiescence, while inverse associations with cell-cycle activity, DNA-damage or repair programs, proliferation, and stemness occurred in selected datasets. These patterns may help reconcile apparently opposing bulk-tissue findings. For example, one lineage may show an invasion-associated state while another shows reduced proliferative activity. Although single-cell analysis reduces the averaging inherent in bulk transcriptomics, it remains affected by dataset size, cellular annotation, transcript dropout, and inter-study heterogeneity [44,45,46]. 

The principal contribution is an integrated description of *WASHC2C*-associated patterns with explicit reconciliation of paralogue uncertainty, resource discordance, adjusted survival results, and external signature evidence. The analyses prioritize contexts for further study while retaining distinctions among single-transcript, family-level protein measurements, and a multigene co-expression score.

Several considerations should be acknowledged when interpreting these findings. Although the study integrates multiple complementary molecular and clinical datasets, heterogeneity in cohort composition, analytical platforms, preprocessing pipelines, event frequencies, and model specifications may have contributed to variability across analyses. The high sequence similarity between *WASHC2C* and *WASHC2A* also constrains complete paralogue-specific interpretation, particularly at the protein level, where available HPA and CPTAC evidence does not definitively resolve WASHC2C-specific abundance. Furthermore, the pathway, immune, copy-number, methylation, and single-cell associations are primarily observational and should be interpreted as biologically informative, hypothesis-generating signals rather than evidence of direct causality. These limitations do not diminish the study’s integrative value; instead, they define clear priorities for subsequent work, including paralogue-resolving assays, functional perturbation studies, validation in independent cohorts, and prospective clinical assessment.

Building on the integrative framework established here, future studies can further define the biological and clinical relevance of *WASHC2C* through increasingly precise, mechanism-focused approaches. Priority should be given to paralogue-resolving transcriptomic assays and protein measurements based on discriminating epitopes or directly observed peptides, enabling unequivocal separation of *WASHC2C* from *WASHC2A*. Functional perturbation studies in cancer- and immune-cell models will be particularly valuable for testing the pathway, trafficking, cytoskeletal, and immune associations identified in this analysis. Validation across independent cohorts, together with prospective outcome studies and treatment-response datasets, will also help determine which cancer-specific *WASHC2C* associations are reproducible, mechanistically relevant, and potentially translatable into prognostic or therapeutic applications.

## 5. Conclusions

This study provides a comprehensive, multi-layered characterization of *WASHC2C* across diverse human cancers, integrating transcriptomic, clinical, genomic, epigenetic, pathway, immune, protein-related, and single-cell evidence. Collectively, the findings position *WASHC2C* as a context-dependent component of cancer biology whose expression, prognostic relevance, molecular associations, and immune relationships vary across tumor lineages rather than conforming to a single oncogenic or tumor-suppressive model.

Importantly, the integrated analyses identify several biologically coherent signals, including cancer-specific expression patterns, copy-number–expression relationships, lineage-dependent pathway associations, immune–microenvironment correlations, and single-cell links to metastatic, inflammatory, hypoxic, invasive, and proliferative states. By explicitly distinguishing *WASHC2C*-specific transcriptomic evidence from paralogue-related protein signals and broader multigene co-expression patterns, this work establishes a rigorous framework for interpreting the gene within the wider WASH-complex landscape. 

Overall, these findings nominate *WASHC2C* as a biologically relevant and cancer-contextualized candidate for further mechanistic investigation. Future paralogue-resolving assays, functional perturbation studies, independent cohort validation, and prospective clinical evaluation will be important for determining which of these molecular associations translate into robust biological mechanisms and clinically meaningful applications.

## Data Availability

Source data were obtained from publicly accessible resources, including TCGA/GDC, GTEx, UCSC Xena, CPTAC/UALCAN, the Human Protein Atlas, GSCA, GEPIA2, SMART, TIMER2.0, TISMO, CancerSEA, and the Gene Expression Omnibus (GSE31210 for the retained external signature analysis). The Supplementary Materials provide numerical results, analytical specifications, and locked gene-signature membership. Separate reproducibility packages for the *WASHC2C* analyses and the UCEC cohort reconciliation are provided as Supplementary Materials. Source availability and remaining reproducibility limitations are described in Supplementary Section S5.

## Supplementary Materials

The following supporting information can be downloaded at: https://www.mdpi.com/article/10.3390/cimb48101046/s1.

## Abbreviations

BH, Benjamini–Hochberg; CI, confidence interval; CNV, copy-number variation; CPTAC, Clinical Proteomic Tumor Analysis Consortium; DFI, disease-free interval; DSS, disease-specific survival; EMT, epithelial–mesenchymal transition; FDR, false discovery rate; GDC, Genomic Data Commons; GEO, Gene Expression Omnibus; GSCA, Gene Set Cancer Analysis; GSEA, gene set enrichment analysis; GSVA, gene set variation analysis; GTEx, Genotype-Tissue Expression; HPA, Human Protein Atlas; HR, hazard ratio; IHC, immunohistochemistry; MSI/MMR, microsatellite instability/mismatch repair; NE, not estimable; NES, normalized enrichment score; OS, overall survival; PFI, progression-free interval; PFS, progression-free survival; PH, proportional hazards; RSEM, RNA-Seq by Expectation Maximization; SD, standard deviation; SNV, single-nucleotide variant; ssGSEA, single-sample GSEA; TCGA-CDR, TCGA Clinical Data Resource; TPM, transcripts per million; WT, wild type or copy-number-neutral, according to context. Cancer abbreviations follow TCGA cohort labels.
