# Supplementary material for: Integrated Multi-Omics Characterization of WASHC2C Reveals Cancer-Specific Molecular, Clinical, Immune, and Cellular Associations
Source: Curr Issues Mol Biol. 2026 Oct 9;48(10):1046. doi: 10.3390/cimb48101046 (PMC13703147; doi:10.3390/cimb48101046)
Supplement: Supplementary file 1 [file cimb-48-01046-s001.zip › cimb-4527153-supplementary.pdf]

WASHC2C Supplementary Materials

S1. Supplementary Methods

S1.1. Harmonized expression and cohort selection

Harmonized TCGA STAR-Counts files were obtained through the GDC API for BLCA, BRCA, COAD, HNSC, LIHC, LUAD, LUSC, and PRAD. The analysis records specify Data Release 46.0 (10 August 2026; API 8.5.0), access on 8 September 2026, and 4,893 downloads passing checksum checks. *WASHC2A* (ENSG00000099290.17) and *WASHC2C* (ENSG00000172661.19), annotated using GENCODE v36, were extracted from the same files. Primary tumors and solid-tissue normals were retained, selecting one file per participant and sample type by ascending sample barcode and file UUID. The final set comprised 4,365 tumors and 429 normal specimens (Table S1).

Primary co-expression analyses used Spearman correlation within each cancer's primary tumors. Normal-only and pooled correlations were secondary. The Benjamini–Hochberg correction was applied across eight cancers within each correlation type. Tumor–normal effect sizes were calculated as log2 ratios of mean unstranded TPM values, with no pseudocount. Two-sided Mann–Whitney tests compared distributions, with Benjamini–Hochberg correction for 16 gene–cancer tests. Percentile confidence intervals used 2,000 bootstrap replicates with seed 20260908. These intervals and tests analyze different estimands; a significant rank test does not imply the mean-ratio interval excludes zero. These are TPM-based comparisons, not differential expression from negative-binomial models.

Matched-pair Wilcoxon signed-rank tests compared within-participant tumor-minus-normal log2(TPM + 1) differences, with BH correction across 16 gene–cancer comparisons. Unlike the paired tests, the full-cohort Mann–Whitney tests and separate-group bootstrap intervals did not model within-participant dependence. A secondary analysis compared log2[(*WASHC2C* TPM + 1)/(*WASHC2A* TPM + 1)] distributions using BH correction across eight cancers. Concordant directions do not establish paralogue specificity or exclude mapping effects (Tables S2–S5).

S1.2. Theoretical sequence distinguishability

Sequence analysis used GENCODE v36 transcript FASTA and GTF files and the GRCh38 primary-assembly genomic FASTA. The primary assembly excludes alternate loci and patches and differs from the GDC GRCh38.d1.vd1 reference. Ten *WASHC2C* transcripts, including retained-intron transcripts, and nine *WASHC2A* transcripts were considered. Distinct 75-, 100-, and 150-base k-mers were compared in both orientations. A separate within-exon analysis used exact genomic matches, with one occurrence defining uniqueness; transcript-position-weighted results were retained separately (Tables S6–S7).

These analyses describe theoretical zero-mismatch sequence distinguishability. They do not model read pairs, splice-aware alignment, sequencing errors, patient variants, expression-weighted isoforms, or additional GDC reference sequences. No patient-read unique-mapping fraction was calculated. The theoretical proportions are not evidence of observed paralogue-specific read assignment.

S1.3. Protein specificity and signature identity

HPA antibody records and their reported other-gene antigen matches were reviewed. The predefined 50-gene signature was audited using Ensembl identifiers against GENCODE v36 and the UCSC hg38 cytoband table. Genes within the locked 50-gene set overlapping 10q11 were listed. CPTAC specificity was treated as unresolved unless observed cohort-specific peptides could be mapped uniquely using the corresponding search database. A supplementary in-silico tryptic digest (7–35 residues, zero to two missed cleavages, cleavage after K/R except before P, I/L treated as indistinguishable) was used only to enumerate candidate peptides differing from *WASHC2A* and was not treated as evidence of peptide detection in CPTAC.

The signature audit preserved the original 50 Ensembl gene identities and reported Pearson correlation coefficients. Historical labels and current symbols are reconciled in Table S11. *WASHC2C* is not a signature member. The exact historical GEPIA2 query settings and generation history are not established by the retained table.

S1.4. Covariate adjusted TCGA survival models

GDC-harmonized STAR-TPM expression from UCSC Xena was linked to the TCGA Clinical Data Resource (TCGA-CDR) for LUAD, BLCA, and LIHC (Liu et al., 2018; UCSC Xena GDC hub). *WASHC2C* (former symbol *FAM21C*; ENSG00000172661.19) was verified using GENCODE v36 and NCBI Gene 253725 (NCBI Gene 253725). Primary tumors were matched by 12-character patient barcode; one sample per patient was retained based on lexicographic barcode order, excluding 12 duplicate LUAD and three duplicate BLCA samples. No GTEx samples were included. The supplied log2(TPM + 1) expression was used without further transformation.

Models included continuous *WASHC2C*, age at initial pathological diagnosis, recorded sex, and categorical pathological stage. Substages were collapsed into stages I–IV; stage 0/X, missing/nonstandard stages, redacted records, missing covariates or endpoints, and nonpositive times were excluded. A complete-case analysis was used with no imputation. Age HRs represent 10-year increases; female sex was the reference. Stage I was the reference in LUAD/LIHC, and stage II in BLCA.

TCGA-CDR indicators were coded 1 = event and 0 = censored; times were measured in days from diagnosis. OS denotes all-cause death; PFI, progression, recurrence, a new tumor event, or death with tumor; DSS, an approximate cancer-specific death based on death with tumor; and DFI, a new tumor event among patients classified as disease-free. Other deaths were censored for DSS; event-free follow-up was censored for interval endpoints. DFI retains the source diagnosis-based surrogate time origin. These TCGA-CDR interval definitions are distinct from GSCA-labeled PFS.

Unpenalized Cox models used Efron ties, Wald 95% CIs, and two-sided Wald p values. Benjamini–Hochberg FDR adjustment covered all 12 planned *WASHC2C* tests; two non-estimable tests occupied $p = 1$ slots only for correction. Schoenfeld residual score tests assessed proportional hazards (PH), and variance inflation factors (VIFs) assessed collinearity. The maximum primary VIF was 1.386. Computation used Python (NumPy/SciPy), and the Efron implementation was checked against a published R survival benchmark. Recorded versions were Python 3.12.14, NumPy 2.3.5, pandas 2.2.3, SciPy 1.17.0, matplotlib 3.10.8, openpyxl 3.1.5, and python-docx 1.2.0. The optional R script was supplied but was not executed in the original analysis.

High expression in the median-split sensitivity analysis was defined as above the cohort median, with low expression at or below the median. Thresholds were fixed before covariate or endpoint exclusions: LUAD 4.1625, BLCA 4.0240, and LIHC 3.6015 on the log2(TPM + 1) scale. Models retained age, sex, and categorical stage; BH correction covered 12 median-split tests. Stage-stratified models and 90-day DFI landmark analyses were evaluated separately. The landmark analysis required OS.time > 90 and DFI.time > 90 and subtracted 90 days from DFI.time.

Sources were the TCGA Clinical Data Resource (Liu et al., Cell 2018;173:400–416.e11; doi:10.1016/j.cell.2018.02.052), UCSC Xena GDC hub STAR-TPM files (https://gdc.xenahubs.net; metadata versions 05-10-2024 for LUAD and 05-09-2024 for BLCA/LIHC), and NCBI Gene 253725. Access was recorded on 11 September 2026.

S1.5. GSE31210 signature assessment

GSE31210 was evaluated as a secondary external analysis. GSE31210 is a non-TCGA stage I–II LUAD cohort from the National Cancer Center, Japan, profiled on Affymetrix GPL570 arrays. Its source publication is Okayama et al. (Cancer Res. 2012;72:100–111; doi:10.1158/0008-5472.CAN-11-1403). Study provenance and metadata support an external cohort, but a patient/genotype crosswalk with TCGA was unavailable. Of 246 deposited profiles, 226 tumors were scored and 20 non-tumor profiles were omitted. Source-defined exclusions for incomplete resection or adjuvant therapy removed 22 tumors from survival analysis, leaving 204 patients (Table S16).

The 50-gene signature was locked before external analysis and excluded *WASHC2C*. No genes were reselected or substituted. MAS5 intensities were log2-transformed. Stable identifiers and unambiguous gene symbols were

mapped using a frozen HGNC annotation; ambiguous probes were excluded, and eligible probes per gene were aggregated using their within-sample median. The same 48 measurable genes were used in every tumor; *WASHC2A* and *PARGP1* were unavailable (Table S11). Thus, neither *WASHC2C* nor *WASHC2A* contributed to the 48-gene external score. GSVA used all 19,759 background genes with a Gaussian kernel, tau = 1, and maximum-difference scoring. ssGSEA used rank normalization and alpha = 0.25 as a scoring sensitivity analysis.

GSVA parameters were Gaussian kernel, tau = 1, maximum-difference scoring, abs_rnk = False, min_size = 15, max_size = 1000, seed = 123, and four threads. ssGSEA used rank sample normalization, rank correlation normalization, alpha = 0.25, no permutations, and the same gene-set size limits and seed. Cohort-standardized scores used the sample standard deviation (ddof = 1).

Unpenalized Cox models used continuous scores standardized across the 204 eligible patients and Efron handling of tied events. OS was adjusted for stage; the source-defined relapse endpoint was adjusted for age, sex, stage, and smoking. OS adjustment was limited by 30 deaths and the prespecified minimum of 10 events per fitted parameter. All covariates were complete; no values were imputed. Endpoints retained deposited event and censoring times in days; death was not added as a relapse event. Scaled Schoenfeld tests assessed proportional hazards. Median grouping was used only for Kaplan–Meier visualization, without cutoff optimization. Nominal p values are reported for the retained models; no revised multiplicity correction was calculated. Analyses used Python 3.12.14, GSEApy 1.3.1, and lifelines 0.30.3.

S1.6. UCEC mutation and PFS reconciliation

To reconcile the UCEC cohorts, the GSCA files UCEC_maf_data.IdTrans.tsv.gz and UCEC.survival.tsv.gz were downloaded on 2 October 2026. Mutation data were obtained from the GSCA-processed MC3 v0.2.8 PUBLIC dataset (Synapse syn7824274; GRCh37). All seven mutation classes used by the portal were retained, and no additional exclusions were applied based on the FILTER field. Sample barcodes were mapped to 12-character patient identifiers. All source tumor samples were included in the mutation-frequency analysis. For the corrected progression-free survival (PFS) analysis, one primary tumor per patient was retained, and records without a valid endpoint time or binary event status were excluded.

PFS was defined using the GSCA pfs_days and pfs_status fields, and survival time was converted to months by dividing days by 30. These fields were confirmed to match the PFS.time and PFS columns, respectively, in the TCGA Clinical Data Resource (TCGA-CDR) ExtraEndpoints worksheet for all 548 UCEC records. Under this definition, a qualifying new tumor event or death from any cause was counted as an event; otherwise, patients were censored. In the reconciled cohort, 24 additional deaths were classified as events compared with the TCGA-CDR progression-free interval (PFI) endpoint. PFS and PFI were therefore treated as distinct endpoints. Kaplan–Meier estimates were recalculated, a two-sided log-rank test was performed, and an unadjusted Cox proportional hazards model was fitted using the Efron method for ties. The corresponding 95% confidence intervals were calculated using the Wald method.

S1.7. Exploratory murine treatment response

TISMO was used for exploratory murine *Washc2* comparisons in syngeneic tumor models receiving immune-checkpoint blockade or related interventions. Baseline, responder, and non-responder groups were examined within each model, treatment, and sampling time. The source framework uses DESeq2 pairwise contrasts with BH correction. Figure S6 retains the source annotations. A complete contrast table, response-group counts, exact significance thresholds, and analysis scripts were not available in the supplied materials. These heterogeneous mouse observations provide biological context only and do not validate human *WASHC2C* or clinical response prediction.

S2. Supplementary Results

S2.1. Harmonized paralogue expression

Within primary tumors, *WASHC2A* and *WASHC2C* were strongly positively correlated across all eight cancers (Spearman ρ 0.712–0.873; all BH-FDR $\leq 6.79 \times 10^{-78}$). Both genes had lower mean expression in BLCA, BRCA,

COAD, LUAD, and LUSC, and higher mean expression in HNSC, LIHC, and PRAD. Thus, all eight comparisons were directionally concordant; none showed opposite tumor–normal directions. Both full-cohort distributional comparisons passed FDR<0.05 in seven cancers. In PRAD, *WASHC2A* was increased (log2FC 0.405; FDR 2.81×10^{-4}), whereas the smaller increase in *WASHC2C* was not significant in the full-cohort comparison (log2FC 0.111; FDR 0.403).

Paired-sensitivity analyses showed that BLCA was not robust to sample pairing (19 pairs; *WASHC2A* FDR 0.180; *WASHC2C* FDR 0.441). For PRAD, the paired *WASHC2C* comparison was significant (52 pairs; median within-pair log2(TPM+1) difference 0.172; FDR 0.0177), indicating that the inference depends on the sample set and estimand. Both paralogues remained significant in paired comparisons for the other six cancers. Relative C-to-A expression differed between tumor and normal distributions in BRCA, COAD, LIHC, LUSC and PRAD after BH correction, but not in BLCA, HNSC or LUAD. These magnitude differences do not establish opposite regulation or exclude mapping-related effects. Numerical results are provided in Tables S1–S5 and Figures S1–S2.

S2.2. Sequence and protein specificity

For 75-, 100-, and 150-base sequences, 3,187/6,197 (51.43%), 3,776/6,347 (59.49%), and 4,794/6,647 (72.12%) distinct *WASHC2C* transcript k-mers had no exact match to *WASHC2A* transcript sequences. In the separate within-exon whole-primary-genome analysis, 1,748/3,554 (49.18%), 1,809/2,975 (60.81%), and 1,848/2,222 (83.17%) distinct k-mers matched exactly one genomic location. Every genomically unique k-mer also lacked an exact match to a *WASHC2A* transcript. These results demonstrate partial, read-length-dependent theoretical distinguishability under the stated exact-match conditions. They do not establish the observed unique-read fraction in TCGA samples or validate a splice-aware, mismatch-tolerant alignment workflow.

HPA reports 100% antigen identity to *WASHC2A* for HPA047844, HPA060975, and HPA061022. The images are therefore interpreted as *WASHC2A*/*WASHC2C*-family immunoreactivity. This is a reported antigen-sequence assessment, not a quantitative comparison of antibody binding to both proteins. Image-level antibody/specimen identifiers remain unresolved. HPA antibody record: https://www.proteinatlas.org/ENSG00000172661-*WASHC2C*/summary/antibody.

Observed CPTAC unique/shared peptide counts could not be established for BRCA, COAD, HNSC, LIHC, LUAD, or UCEC. The theoretical digest yielded 302 distinct candidate peptides, of which 216 also occurred in *WASHC2A* translations after I/L equivalence, and 86 did not. These are candidate counts across GENCODE translations, not observed cohort counts, and absence from *WASHC2A* does not establish uniqueness across the entire human proteome. Therefore, the current protein-level findings do not establish *WASHC2C*-specific proteomic validation. The full theoretical candidate list is retained in Table S12.

S2.3. Adjusted TCGA survival

Higher *WASHC2C* expression was associated with poorer LIHC OS (HR 1.612, 95% CI 1.177–2.208; p = 0.002914; FDR = 0.034968). No other evaluable primary association survived FDR correction. LIHC DSS violated gene-specific PH (p = 0.007165); global PH tests were significant for LUAD PFI/DSS, BLCA DFI, and LIHC DSS (Table S13). These findings support an adjusted association but do not establish causality or validate prognostic performance.

Stage-stratified models gave BLCA OS HR 1.055 (95% CI 0.793–1.403; p = 0.715317) and DSS HR 1.191 (0.838–1.692; p = 0.330232). The LIHC OS estimate remained adverse (HR 1.573, 1.145–2.162; p = 0.005225), but its separately adjusted FDR was 0.062698. No 90-day DFI landmark association survived correction across three cohorts; this analysis required OS.time > 90 and DFI.time > 90, then subtracted 90 days from DFI.time.

No median-split association met FDR < 0.05 (Table S15). The primary LIHC OS result is an adjusted observational association with sensitivity to model specification. Cohort selection following univariate observations, complete-case exclusions, sparse stage groups, approximate DSS classification, diagnosis-based DFI time origin, and unmeasured clinical factors limit inference. Full coefficients, PH diagnostics, and median-split results are provided in Tables S13–S15; Figure S4 displays primary adjusted HRs.

S2.4. Signature membership and GSE31210 assessment

The predefined signature comprises 50 unique Ensembl gene identifiers. *WASHC2A* ranks first in the archived table (reported Pearson PCC = 0.80). Six genes in the locked 50-gene set overlap cytoband 10q11: *WASHC2A*, *MARCHF8*, *PARGP1*, *ZFAND4*, *BMS1P1*, and *PARG*. The date-corrupted label "8-Mar" maps to ENSG00000165406 (*MARCHF8*). Because the signature includes the close paralogue and several regional genes, it is described as *WASHC2C*-associated rather than *WASHC2C*-specific. This pattern does not by itself establish technical artifact; regional co-regulation remains an alternative explanation.

In GSE31210, 48 of the locked 50 genes were measurable; *WASHC2A* and *PARGP1* were unavailable, and *WASHC2C* was not a signature member (Table S11). The survival set comprised 204 patients, with 30 deaths and 54 relapse events (Table S16). Higher GSVA scores were associated with favorable stage-adjusted OS (HR per SD 0.461, 95% CI 0.303–0.703; nominal $p = 3.1518803 \times 10^{-4}$) and relapse after adjustment for age, sex, stage, and smoking (HR 0.516, 95% CI 0.380–0.701; nominal $p = 2.3129707 \times 10^{-5}$; Table S17). Median groups contained 102 patients each (Figure S5).

Score-specific Schoenfeld tests were nonsignificant in principal GSE31210 GSVA models (all $p \geq 0.167$), but stage II violated PH in the adjusted OS model ($p = 0.001392$). Diagnostic-triggered stage stratification retained the score association (HR 0.465, 95% CI 0.305–0.709; nominal $p = 3.69876 \times 10^{-4}$). ssGSEA was concordant in GSE31210; its correlation with GSVA was 0.957741 across 226 tumors. These findings provide cohort-specific support for the measurable co-expression program and do not validate either paralogue, the complete 50-gene signature, or clinical utility.

S2.5. Reconciled UCEC cohort

The mutation-frequency cohort comprised 531 tumor samples from 530 unique patients, including 35 mutation-positive samples from 35 distinct patients (6.59%). One wild-type patient, TCGA-BK-A139, contributed primary and recurrent samples. TCGA-AJ-A3BH carried an eligible frameshift deletion but lacked PFS time and was excluded from survival analysis. The historical portal comparison therefore contained 530 observations from 529 unique patients.

After retaining one primary tumor per patient, the corrected PFS cohort contained 529 patients: 34 mutant patients with 3 events and 495 wild-type patients with 141 events (144 total). The nominal log-rank *p* value was 0.00437; the unadjusted mutant-versus-wild-type HR was 0.219 (95% CI 0.069–0.688; Wald $p = 0.00939$). These results correspond to manuscript Sections 3.7–3.8 and Figure 12B. Only three mutant-group events support the exploratory association; no protective, mechanistic, or clinically useful effect is inferred.

S2.6. Exploratory murine observations

Murine *Washc2* expression varied across experimental conditions and showed no uniform association with immune-checkpoint blockade response. Lower responder expression was observed in selected T11 UV, KPB25L, radiation, CT26, EMT6, MC38, and B16 conditions, whereas higher responder expression occurred in selected 4T1, BNL-MEA, KPB25L end-treatment, T11 end-treatment, and YTN16 conditions. Many comparisons showed overlapping distributions. Overall, the result was model-, treatment-, and time-point-dependent and did not support a consistent standalone response marker. The complete supplied source graphic and enlarged consecutive views are provided in Figure S6.

S3. Supplementary Tables

Table S1. Primary tumor paralogue correlations and harmonized sample counts

Cancer	Tumor n	Normal n	ρ	Nominal *p*	BH FDR
BLCA	406	19	0.784	1.34×10^{-85}	1.53×10^{-85}
BRCA	1095	113	0.805	5.81×10^{-250}	4.64×10^{-249}
COAD	458	41	0.873	1.8×10^{-144}	4.79×10^{-144}
HNSC	520	44	0.820	1×10^{-127}	2.01×10^{-127}
LIHC	371	50	0.865	1.39×10^{-112}	1.85×10^{-112}
LUAD	517	59	0.851	1.91×10^{-146}	7.63×10^{-146}
LUSC	501	51	0.812	1.49×10^{-118}	2.39×10^{-118}
PRAD	497	52	0.712	6.78×10^{-78}	6.78×10^{-78}

Both genes use the same selected files. Spearman correlations use tumors only; BH correction covers eight cancers. Tumor–normal directions are concordant in every cohort. n, number of samples; ρ, Spearman correlation coefficient; BH, Benjamini–Hochberg; FDR, false discovery rate. BLCA, bladder urothelial carcinoma; BRCA, breast invasive carcinoma; COAD, colon adenocarcinoma; HNSC, head and neck squamous cell carcinoma; LIHC, liver hepatocellular carcinoma; LUAD, lung adenocarcinoma; LUSC, lung squamous cell carcinoma; PRAD, prostate adenocarcinoma.

Table S2. Tumor versus normal expression of *WASHC2A* and *WASHC2C*

Cancer / gene	T / N	log2FC	95% CI	Nominal *p*	BH FDR
BLCA / *WASHC2A*	406/19	−0.248	−0.453, −0.028	0.0267	0.0305
BLCA / *WASHC2C*	406/19	−0.203	−0.404, 0.028	0.0445	0.0475
BRCA / *WASHC2A*	1095/113	−0.199	−0.273, −0.123	1.96×10^{-8}	3.91×10^{-8}
BRCA / *WASHC2C*	1095/113	−0.297	−0.379, −0.215	3.23×10^{-11}	8.61×10^{-11}
COAD / *WASHC2A*	458/41	−0.399	−0.480, −0.311	2.77×10^{-8}	4.92×10^{-8}
COAD / *WASHC2C*	458/41	−0.785	−0.897, −0.663	8.06×10^{-17}	4.3×10^{-16}
HNSC / *WASHC2A*	520/44	0.345	0.201, 0.500	2.11×10^{-4}	2.81×10^{-4}
HNSC / *WASHC2C*	520/44	0.330	0.171, 0.491	2.72×10^{-4}	3.35×10^{-4}
LIHC / *WASHC2A*	371/50	1.499	1.304, 1.709	1.67×10^{-23}	2.67×10^{-22}
LIHC / *WASHC2C*	371/50	1.289	1.120, 1.469	4.14×10^{-21}	3.32×10^{-20}
LUAD / *WASHC2A*	517/59	−0.376	−0.486, −0.264	5.61×10^{-9}	1.28×10^{-8}
LUAD / *WASHC2C*	517/59	−0.278	−0.385, −0.165	5.97×10^{-6}	9.55×10^{-6}
LUSC / *WASHC2A*	501/51	−0.673	−0.821, −0.523	1.31×10^{-15}	5.24×10^{-15}
LUSC / *WASHC2C*	501/51	−0.443	−0.544, −0.342	8.59×10^{-12}	2.75×10^{-11}
PRAD / *WASHC2A*	497/52	0.405	0.252, 0.566	1.94×10^{-4}	2.81×10^{-4}
PRAD / *WASHC2C*	497/52	0.111	−0.042, 0.284	0.403	0.403

Effect = log2(mean tumor TPM/mean normal TPM). Intervals are descriptive separate-group bootstrap 95% intervals; *p* values are two-sided Mann–Whitney distributional tests, BH across 16 tests. The *p* test and CI address different estimands. Full-cohort tests do not model within-participant dependence; see Table S3. BH, Benjamini–Hochberg; FDR, false discovery rate; TPM, transcripts per million; CI, confidence interval; T, tumor; N, normal; log2FC, log2 fold change. BLCA, bladder urothelial carcinoma; BRCA, breast invasive carcinoma; COAD, colon adenocarcinoma; HNSC, head and neck squamous cell carcinoma; LIHC, liver hepatocellular carcinoma; LUAD, lung adenocarcinoma; LUSC, lung squamous cell carcinoma; PRAD, prostate adenocarcinoma.

Table S3. Matched tumor normal sensitivity analyses

Cancer / gene	Pairs	Median paired Δ	Nominal *p*	BH FDR
BLCA / *WASHC2A*	19	−0.261	0.169	0.18
BLCA / *WASHC2C*	19	−0.076	0.441	0.441
BRCA / *WASHC2A*	113	−0.235	1.36×10^{-4}	1.98×10^{-4}
BRCA / *WASHC2C*	113	−0.227	3.82×10^{-5}	6.79×10^{-5}
COAD / *WASHC2A*	41	−0.558	1.48×10^{-6}	4.66×10^{-6}
COAD / *WASHC2C*	41	−0.972	2.51×10^{-9}	1.34×10^{-8}
HNSC / *WASHC2A*	43	0.198	0.0178	0.0219
HNSC / *WASHC2C*	43	0.172	0.0375	0.0428
LIHC / *WASHC2A*	50	1.291	4.91×10^{-12}	3.93×10^{-11}
LIHC / *WASHC2C*	50	1.015	3.09×10^{-12}	3.93×10^{-11}
LUAD / *WASHC2A*	58	−0.392	1.21×10^{-7}	4.84×10^{-7}
LUAD / *WASHC2C*	58	−0.308	3.62×10^{-5}	6.79×10^{-5}
LUSC / *WASHC2A*	51	−0.485	1.75×10^{-6}	4.66×10^{-6}
LUSC / *WASHC2C*	51	−0.316	5.56×10^{-5}	8.9×10^{-5}
PRAD / *WASHC2A*	52	0.489	1.18×10^{-5}	2.71×10^{-5}
PRAD / *WASHC2C*	52	0.172	0.0132	0.0177

Δ = within-pair tumor minus normal log2(TPM+1). Two-sided Wilcoxon signed-rank tests; BH across 16 tests. BLCA is nonsignificant for both genes. PRAD *WASHC2C* differs from the full-cohort inference. BH, Benjamini–Hochberg; FDR, false discovery rate; TPM, transcripts per million; ρ, Spearman correlation coefficient. BLCA, bladder urothelial carcinoma; BRCA, breast invasive carcinoma; COAD, colon adenocarcinoma; HNSC, head and neck squamous cell carcinoma; LIHC, liver hepatocellular carcinoma; LUAD, lung adenocarcinoma; LUSC, lung squamous cell carcinoma; PRAD, prostate adenocarcinoma.

Table S4. Secondary paralogue correlations in normal and pooled samples

Cancer	Sample set	n	ρ	Nominal *p*	BH FDR
BLCA	Normal	19	0.888	3.97×10^{-7}	5.3×10^{-7}
BLCA	Pooled	425	0.791	2.76×10^{-92}	3.16×10^{-92}
BRCA	Normal	113	0.803	1.17×10^{-26}	9.37×10^{-26}
BRCA	Pooled	1208	0.810	1.86×10^{-281}	1.49×10^{-280}
COAD	Normal	41	0.594	4.17×10^{-5}	4.76×10^{-5}
COAD	Pooled	499	0.871	2.8×10^{-155}	7.48×10^{-155}
HNSC	Normal	44	0.881	2.92×10^{-15}	1.17×10^{-14}
HNSC	Pooled	564	0.828	2.26×10^{-143}	3.62×10^{-143}
LIHC	Normal	50	0.824	1.92×10^{-13}	3.83×10^{-13}
LIHC	Pooled	421	0.893	9.52×10^{-148}	1.9×10^{-147}
LUAD	Normal	59	0.713	2.38×10^{-10}	3.81×10^{-10}
LUAD	Pooled	576	0.852	2.45×10^{-163}	9.8×10^{-163}
LUSC	Normal	51	0.536	4.97×10^{-5}	4.97×10^{-5}
LUSC	Pooled	552	0.823	3.39×10^{-137}	4.51×10^{-137}
PRAD	Normal	52	0.830	2.78×10^{-14}	7.41×10^{-14}
PRAD	Pooled	549	0.718	3.04×10^{-88}	3.04×10^{-88}

Normal-only and pooled analyses are secondary. BH is applied separately across eight cancers within each sample-set family. Pooled estimates can reflect tumor–normal group differences. BH, Benjamini–Hochberg; FDR, false discovery rate; TPM, transcripts per million; ρ, Spearman correlation coefficient. BLCA, bladder urothelial carcinoma; BRCA, breast invasive carcinoma; COAD, colon adenocarcinoma; HNSC, head and neck squamous cell carcinoma; LIHC, liver hepatocellular carcinoma; LUAD, lung adenocarcinoma; LUSC, lung squamous cell carcinoma; PRAD, prostate adenocarcinoma.

Table S5. Relative expression of *WASHC2C* and *WASHC2A*

Cancer	Median ratio shift	Nominal *p*	BH FDR
BLCA	0.020	0.542	0.619
BRCA	−0.053	0.0174	0.0278
COAD	−0.408	4.55×10^{-13}	3.64×10^{-12}
HNSC	0.009	0.706	0.706
LIHC	−0.169	2.1×10^{-5}	8.42×10^{-5}
LUAD	0.065	0.0579	0.0772
LUSC	0.133	0.00177	0.00353
PRAD	−0.236	3.59×10^{-5}	9.57×10^{-5}

Tumor minus normal median of log2[(*WASHC2C* TPM+1)/(*WASHC2A* TPM+1)]. Two-sided Mann–Whitney tests, BH across eight cancers. Pseudocount-dependent; a relative magnitude difference is not opposite regulation. BH, Benjamini–Hochberg; FDR, false discovery rate; TPM, transcripts per million; ρ, Spearman correlation coefficient. BLCA, bladder urothelial carcinoma; BRCA, breast invasive carcinoma; COAD, colon adenocarcinoma; HNSC, head and neck squamous cell carcinoma; LIHC, liver hepatocellular carcinoma; LUAD, lung adenocarcinoma; LUSC, lung squamous cell carcinoma; PRAD, prostate adenocarcinoma.

Table S6. Theoretical exact match sequence distinguishability

Length (bp)	No *WASHC2A* match / total	Transcript (%)	One genomic hit / total	Genome (%)
75	3187/6197	51.43	1748/3554	49.18
100	3776/6347	59.49	1809/2975	60.81
150	4794/6647	72.12	1848/2222	83.17

Transcript and within-exon genomic analyses have different denominators. Genome = GRCh38 primary assembly, both strands, exact matches only. All within-exon sequences had a genomic hit. No sequencing-error, mismatch-tolerant, splice-aware or paired-end model; these are not observed read-level fractions. bp, base pairs; GENCODE, reference genome annotation consortium.

Table S7. Transcript specific pairwise sequence distinguishability

***WASHC2C* transcript**	**Length (bp)**	**Positions**	**No *WASHC2A* match**	**Percentage**
ENST00000359860.7	75	4397	2089	47.51
ENST00000374362.6	75	4549	2075	45.61
ENST00000540872.6	75	4253	1903	44.74
ENST00000537517.6	75	3966	1891	47.68
ENST00000336378.8	75	4595	1983	43.16
ENST00000623400.4	75	4568	2031	44.46
ENST00000420848.3	75	794	367	46.22
ENST00000485850.1	75	199	0	0.00
ENST00000374359.1	75	711	385	54.15
ENST00000471102.1	75	1944	1277	65.69
ENST00000359860.7	100	4372	2538	58.05
ENST00000374362.6	100	4524	2524	55.79
ENST00000540872.6	100	4228	2302	54.45
ENST00000537517.6	100	3941	2299	58.34
ENST00000336378.8	100	4570	2407	52.67
ENST00000623400.4	100	4543	2480	54.59
ENST00000420848.3	100	769	431	56.05
ENST00000485850.1	100	174	0	0.00
ENST00000374359.1	100	686	450	65.60
ENST00000471102.1	100	1919	1398	72.85
ENST00000359860.7	150	4322	3196	73.95
ENST00000374362.6	150	4474	3250	72.64
ENST00000540872.6	150	4178	2928	70.08
ENST00000537517.6	150	3891	2888	74.22
ENST00000336378.8	150	4520	3100	68.58
ENST00000623400.4	150	4493	3206	71.36
ENST00000420848.3	150	719	449	62.45
ENST00000485850.1	150	124	0	0.00
ENST00000374359.1	150	636	524	82.39
ENST00000471102.1	150	1869	1585	84.80

All ten GENCODE v36 *WASHC2C* transcripts, including retained-intron transcripts. Counts are transcript positions and can repeat identical sequence across isoforms; no isoform-expression weighting was applied. bp, base pairs; GENCODE, reference genome annotation consortium.

Table S8. HPA antibody antigen identity with *WASHC2A*

Antibody	Other gene	Antigen identity	First HPA release
HPA047844	*WASHC2A*	100%	10.0
HPA060975	*WASHC2A*	100%	14.0
HPA061022	*WASHC2A*	100%	18.0

HPA-reported antigen matches, accessed 8 September 2026. First release denotes the first HPA release containing each antibody, not the current database version. None of these reported antigen identities supports paralogue-selective staining. HPA, Human Protein Atlas.

Table S9. Availability of observed CPTAC peptide specificity evidence

Cohort	Unique peptides	Shared peptides	Assessment
BRCA	Not established	Not established	Unresolved
COAD	Not established	Not established	Unresolved
HNSC	Not established	Not established	Unresolved
LIHC	Not established	Not established	Unresolved
LUAD	Not established	Not established	Unresolved
UCEC	Not established	Not established	Unresolved

Observed cohort-specific peptide/PSM exports, protein-group assignments, identification q-values, and matching search FASTA versions were not available. Missing evidence is not a count of zero peptides. The candidates in Table S12 do not establish observed peptide detection or the identity of the UALCAN protein group. CPTAC, Clinical Proteomic Tumor Analysis Consortium; PSM, peptide-spectrum match; UALCAN, cancer data analysis portal; UCEC, uterine corpus endometrial carcinoma. BLCA, bladder urothelial carcinoma; BRCA, breast invasive carcinoma; COAD, colon adenocarcinoma; HNSC, head and neck squamous cell carcinoma; LIHC, liver hepatocellular carcinoma; LUAD, lung adenocarcinoma; LUSC, lung squamous cell carcinoma; PRAD, prostate adenocarcinoma.

Table S10. Locked signature genes overlapping chromosome 10q11

Gene	Rank	Ensembl gene ID	Chromosome / band	Source PCC
WASHC2A	1	ENSG00000099290	10 q11.23	0.80
MARCHF8	2	ENSG00000165406	10 q11.21;q11.22	0.71
PARGP1	4	ENSG00000239883	10 q11.22	0.67
ZFAND4	8	ENSG00000172671	10 q11.22	0.63
BMS1P1	21	ENSG00000204177	10 q11.22	0.61
PARG	25	ENSG00000227345	10 q11.23	0.60

Six genes within the locked 50-gene signature overlap 10q11. PCC values are those reported in the archived source table. GENCODE v36 and UCSC hg38 cytoband coordinates were used. *WASHC2A* lies at 10q11.23 and *WASHC2C* at 10q11.22, approximately 4.3 Mb apart. PCC, Pearson correlation coefficient; rank refers to the original locked signature order.

Table S11. Locked 50 gene signature and GSE31210 coverage

Gene	Rank	Ensembl gene ID	Historical labels	Chr / band	PCC	Measured
WASHC2A	1	ENSG00000099290	*FAM21A/WASHC2A*	10 q11.23	0.80	No
MARCHF8	2	ENSG00000165406	8-Mar	10 q11.21;q11.22	0.71	Yes
EPC1	3	ENSG00000120616	—	10 p11.22	0.67	Yes
PARGP1	4	ENSG00000239883	—	10 q11.22	0.67	No
OGA	5	ENSG00000198408	MGEA5	10 q24.32	0.65	Yes
BLTP1	6	ENSG00000138688	*KIAA1109*	4 q27	0.65	Yes
HEATR5B	7	ENSG00000008869	—	2 p22.2	0.64	Yes
ZFAND4	8	ENSG00000172671	—	10 q11.22	0.63	Yes
RUFY2	9	ENSG00000204130	—	10 q21.3	0.63	Yes
SIRT1	10	ENSG00000096717	—	10 q21.3	0.62	Yes
SLF2	11	ENSG00000119906	FAM178A	10 q24.31	0.62	Yes
ZBTB40	12	ENSG00000184677	—	1 p36.12	0.62	Yes
ARID4A	13	ENSG00000032219	—	14 q23.1	0.62	Yes
MARF1	14	ENSG00000166783	KIAA0430	16 p13.11	0.62	Yes
CSTF2T	15	ENSG00000177613	—	10 q21.1	0.62	Yes
SKIC3	16	ENSG00000198677	*TTC37*	5 q15	0.62	Yes
YTHDC1	17	ENSG00000083896	—	4 q13.2	0.62	Yes
KMT2A	18	ENSG00000118058	—	11 q23.3	0.61	Yes
NDST2	19	ENSG00000166507	—	10 q22.2	0.61	Yes
WAC	20	ENSG00000095787	—	10 p12.1	0.61	Yes
BMS1P1	21	ENSG00000204177	—	10 q11.22	0.61	Yes
KDM3B	22	ENSG00000120733	—	5 q31.2	0.61	Yes
KMT2E	23	ENSG00000005483	—	7 q22.3	0.61	Yes
ZBTB14	24	ENSG00000198081	—	18 p11.31	0.61	Yes
PARG	25	ENSG00000227345	—	10 q11.23	0.60	Yes
INO80D	26	ENSG00000114933	—	2 q33.3	0.60	Yes
DDX17	27	ENSG00000100201	—	22 q13.1	0.60	Yes
ZNF33A	28	ENSG00000189180	—	10 p11.1	0.60	Yes
BTAF1	29	ENSG00000095564	—	10 q23.32	0.60	Yes
IKZF5	30	ENSG00000095574	—	10 q26.13	0.60	Yes
GIGYF2	31	ENSG00000204120	—	2 q37.1	0.60	Yes
THRAP3	32	ENSG00000054118	—	1 p34.3	0.60	Yes
NHLRC2	33	ENSG00000196865	—	10 q25.3	0.60	Yes
ZZEF1	34	ENSG00000074755	—	17 p13.2	0.60	Yes
SENP7	35	ENSG00000138468	—	3 q12.3	0.60	Yes
ELF2	36	ENSG00000109381	—	4 q31.1	0.59	Yes
ZBTB1	37	ENSG00000126804	—	14 q23.3	0.59	Yes
SREK1	38	ENSG00000153914	—	5 q12.3	0.59	Yes
NBR1	39	ENSG00000188554	—	17 q21.31	0.59	Yes
SETD2	40	ENSG00000181555	—	3 p21.31	0.59	Yes

Gene	Rank	Ensembl gene ID	Historical labels	Chr / band	PCC	Measured
BICRAL	41	ENSG00000112624	GLTSCR1L	6 p21.1	0.59	Yes
BOD1L1	42	ENSG00000038219	—	4 p15.33	0.59	Yes
FAM13B	43	ENSG00000031003	—	5 q31.2	0.59	Yes
ZXDA	44	ENSG00000198205	—	X p11.21	0.59	Yes
ZNF248	45	ENSG00000198105	—	10 p11.21	0.59	Yes
ZC3H7A	46	ENSG00000122299	—	16 p13.13	0.59	Yes
CARF	47	ENSG00000138380	—	2 q33.2	0.59	Yes
ZMYND11	48	ENSG00000015171	—	10 p15.3	0.59	Yes
SP1	49	ENSG00000185591	—	12 q13.13	0.59	Yes
UBN2	50	ENSG00000157741	—	7 q34	0.59	Yes

The 50 original Ensembl identities and their order are preserved. Measured indicates availability for the GSE31210 score: 48 genes were measurable, while *WASHC2A* and *PARGP1* were unavailable. *WASHC2C* is not a signature member. The date-corrupted label 8-Mar maps to *MARCHF8*; source labels *KIAA1109* and *TTC37* correspond to the supplied current symbols *BLTP1* and *SKIC3*. *PARGP1* and *BMS1P1* are pseudogenes. Historical labels retain the original and GENCODE v36 names where they differ. Chr, chromosome; PCC, Pearson correlation coefficient from the source table; —, no differing historical label.

Table S12. Theoretical tryptic peptide candidates

302 distinct candidate sequences: 216 also occur in *WASHC2A,* and 86 do not. GENCODE v36 translations; 7–35 residues; zero to two missed cleavages; I/L equivalent. "No" does not establish uniqueness across the human proteome or detection in CPTAC. These are theoretical sequences, not observed peptides. K, lysine; R, arginine; P, proline; I/L, isoleucine/leucine; CPTAC, Clinical Proteomic Tumor Analysis Consortium. Cleavage occurred after K/R, except before P. All 302 supplied sequences are retained.

Peptide sequence	Length (residues)	Also present in *WASHC2A*
AARRLAAQESSEAEDMSVPR	20	No
AASGEDSTEEALAAAAAPWEGGPVPGVDTSPFAK	34	No
AEAEKTEQEK	10	Yes
AEAEKTEQEKTR	12	Yes
AEKKVTLSYSK	11	No
AGNSDSEEDDANGR	14	Yes
AGNSDSEEDDANGRVELILEPK	22	Yes
AKASELSK	8	Yes
AKASELSKK	9	Yes
AKIAENPANPPVGGK	15	Yes
AKIAENPANPPVGGKAK	17	Yes
AKSPMFPALGEASSDDDLFQSAKPKPAK	28	Yes
AKSPMFPALGEASSDDDLFQSAKPKPAKK	29	Yes
ASALLFSSDEEDQWNIPASQTHLASDSR	28	Yes
ASALLFSSDEEDQWNIPASQTHLASDSRSK	30	Yes
ASALLFSSDEEDQWNIPASQTHLASDSRSKGEPR	34	Yes
ASALLFSSDEEWNIPASQTHLASDSR	26	No
ASALLFSSDEEWNIPASQTHLASDSRSK	28	No
ASALLFSSDEEWNIPASQTHLASDSRSKGEPR	32	No
ASELSKK	7	Yes
ASELSKKK	8	Yes
ATDCRLHNVFNDFLMLSNTQFIENR	25	Yes
AVASPEATVSQTDENK	16	Yes
AVASPEATVSQTDENKAR	18	Yes
AVASPEATVSQTDENKARAEK	21	Yes
AVKKTSLFEEDK	12	No
DHSVNSFK	8	No
DHSVNSFKNQK	11	No
DHSVNSFKNQKHPESIQGSK	20	No
DLYIDRPLPYLIGSK	15	Yes
DNDPDVDLFAGTK	13	Yes
DNDPDVDLFAGTKK	14	Yes
DNDPDVDLFAGTKKTK	16	Yes
DSGTLQSQEAK	11	Yes
DSGTLQSQEAKAVK	14	Yes
DSGTLQSQEAKAVKK	15	Yes

**Peptide sequence**	**Length (residues)**	**Also present in *WASHC2A***
DSQKKTQR	8	Yes
EAQLGVK	7	Yes
EAQLGVKSVDK	11	Yes
EAQLGVKSVDKK	12	Yes
EDDLFAIAK	9	Yes
EDDLFAIAKDSQK	13	Yes
EDDLFAIAKDSQKK	14	Yes
EEEDIEDIEENTRPK	15	Yes
EEEDIEDIEENTRPKR	16	Yes
EEEDIEDIEENTRPKRSRPTSFADELAAR	29	Yes
EEKAKASELSK	11	Yes
EESSSSKPGK	10	Yes
EESSSSKPGKK	11	Yes
EGLLTRSAQETVK	13	Yes
EGLLTRSAQETVKHSDLFSSSSPWDK	26	Yes
EKAVASPEATVSQTDENK	18	Yes
EKAVASPEATVSQTDENKAR	20	Yes
EKEKTLESNLFDDNIDIFADLTVKPK	26	Yes
EKGIWKPETPQDSSGLAPFK	20	Yes
EKGIWKPETPQDSSGLAPFKTK	22	Yes
EKTLESNLFDDNIDIFADLTVKPK	24	Yes
EKTLESNLFDDNIDIFADLTVKPKEK	26	Yes
EPQKPEQPTPR	11	Yes
EPQKPEQPTPRK	12	Yes
EPSTRIGK	8	Yes
EQKEVDLIPK	10	Yes
EQKEVDLIPKVQEAVNYGLQVLDSAFEQLDIK	32	Yes
ERRTPSD	7	Yes
ERRTPSDDEEDNLFAPPK	18	Yes
ESLKFGR	7	Yes
ESLKFGRTDVAESEK	15	Yes
ETKATDCR	8	Yes
ETKATDCRLHNVFNDFLMLSNTQFIENR	28	Yes
ETVSEAPPLLFSDEEEK	17	Yes
ETVSEAPPLLFSDEEEKEAQLGVK	24	Yes
ETVSEAPPLLFSDEEEKEAQLGVKSVDK	28	Yes
EVDLIPK	7	Yes
EVDLIPKVQEAVNYGLQVLDSAFEQLDIK	29	Yes
EVGKGCDPDAHPK	13	No
EVGKGCDPDAHPKSTGVFQDEELLFSHK	28	No

**Peptide sequence**	**Length (residues)**	**Also present in *WASHC2A***
FEHKVSNIFDDPLNAFGGQ	19	Yes
FGRTDVAESEK	11	Yes
FGRTDVAESEKEGLLTR	17	Yes
GASLLPGK	8	Yes
GASLLPGKLPTSVSLFDDEDEEDNLFGGTAAK	32	No
GASLLPGKLPTSVSLFDDEDEEDNLFGGTAAKK	33	No
GCDPDAHPK	9	No
GCDPDAHPKSTGVFQDEELLFSHK	24	No
GCDPDAHPKSTGVFQDEELLFSHKLQK	27	No
GDAMGRVDEEPTNEEDNLFAPPK	23	No
GDAMGRVDEEPTTLPSGEAKPR	22	No
GDAMGRVDEEPTTLPSGEAKPRK	23	No
GEADLFDSGDIFSTGTGSQSVER	23	Yes
GEADLFDSGDIFSTGTGSQSVERTKPK	27	Yes
GEADLFDSGDIFSTGTGSQSVERTKPKAK	29	Yes
GEPRDSGTLQSQEAK	15	Yes
GEPRDSGTLQSQEAKAVK	18	Yes
GIWKPETPQDSSGLAPFK	18	Yes
GIWKPETPQDSSGLAPFKTK	20	Yes
GIWKPETPQDSSGLAPFKTKEPSTR	25	Yes
GKRRPQTR	8	Yes
GLFDDEDEESDLFTEASQDR	20	No
GLFDDEDEESDLFTEASQDRQAGASVK	27	No
GLFSDEEDSEDLFSSQSASNLK	22	No
GLFSDEEDSEDLFSSQSASNLKGASLLPGK	30	No
GPIAQWADGAISPNGHRPQLR	21	Yes
GSIVDTEEEK	10	Yes
GTKPRTK	7	Yes
GTKPRTKTVLSLFDEEEDK	19	Yes
HPESIQGSK	9	Yes
HPESIQGSKEK	11	Yes
HPESIQGSKEKGIWKPETPQDSSGLAPFK	29	Yes
HSDLFSSSSPWDK	13	Yes
HSDLFSSSSPWDKGTKPR	18	Yes
HSDLFSSSSPWDKGTKPRTK	20	Yes
IAENPANPPVGGK	13	Yes
IAENPANPPVGGKAK	15	Yes
IKGDAMGR	8	No
IKGDAMGRVDEEPTNEEDNLFAPPK	25	No
IKGDAMGRVDEEPTTLPSGEAKPR	24	No

**Peptide sequence**	**Length (residues)**	**Also present in *WASHC2A***
IPAGAVSVFLGDTDVFGAASVPSLK	25	No
KASALLFSSDEEDQWNIPASQTHLASDSR	29	Yes
KASALLFSSDEEDQWNIPASQTHLASDSRSK	31	Yes
KASALLFSSDEEWNIPASQTHLASDSR	27	No
KASALLFSSDEEWNIPASQTHLASDSRSK	29	No
KDHSVNSFK	9	No
KDHSVNSFKNQK	12	No
KETVSEAPPLLFSDEEEK	18	Yes
KETVSEAPPLLFSDEEEKEAQLGVK	25	Yes
KIPAGAVSVFLGDTDVFGAASVPSLK	26	No
KKASALLFSSDEEDQWNIPASQTHLASDSR	30	Yes
KKASALLFSSDEEWNIPASQTHLASDSR	28	No
KKETVSEAPPLLFSDEEEK	19	Yes
KNETKSSSQQDVILTTQDIFEDDIFATEAIKPSQK	35	No
KQTLSLQAQR	10	No
KQTLSLQAQREEK	13	No
KQVDGLIR	8	Yes
KQVDGLIRETK	11	Yes
KSPYGPPPTGLFDDDDGDDDDDFFSAPHSKPSK	33	Yes
KSPYGPPPTGLFDDDDGDDDDDFFSAPHSKPSKTR	35	No
KTKLLEPSVGSLFGDDEDDDLFSSAK	26	Yes
KTNPFPLLEDEDDLFTDQK	19	Yes
KTNPFPLLEDEDDLFTDQKVK	21	Yes
KTQRVSLLFEDDVDSGGSLFGSPPTSVPPATK	32	Yes
KTSLFEEDK	9	No
KTSLFEEDKEDDLFAIAK	18	No
KVEAKSIFDDDMDDIFSTGIQAK	23	No
KVESAKESLK	10	Yes
KVQSTADIFGDEEGDLFK	18	Yes
KVQSTADIFGDEEGDLFKEK	20	Yes
KVTLSYSK	8	No
KVTLSYSKNLKPSSETK	17	No
LAAQESSEAEDMSVPR	16	No
LFMEQEDVGLGELSSEEGSVGSDR	24	Yes
LFMEQEDVGLGELSSEEGSVGSDRGSIVDTEEEK	34	Yes
LHNVFNDFLMLSNTQFIENR	20	Yes
LHNVFNDFLMLSNTQFIENRVYDEEVEEPVLK	32	Yes
LLEPSVGSLFGDDEDDDLFSSAK	23	Yes
LLEPSVGSLFGDDEDDDLFSSAKSQPLVQEK	31	Yes
LLEPSVGSLFGDDEDDDLFSSAKSQPLVQEKK	32	Yes

**Peptide sequence**	**Length (residues)**	**Also present in *WASHC2A***
LPTSVSLFDDEDEEDNLFGGTAAK	24	No
LPTSVSLFDDEDEEDNLFGGTAAKK	25	No
LPTSVSLFDDEDEEDNLFGGTAAKKQTLSLQAQR	34	No
LQKDNDPDVDLFAGTK	16	Yes
LQKDNDPDVDLFAGTKK	17	Yes
LTDEDFSPFGSGGGLFSGGK	20	Yes
MEDQNIIQAPQK	12	Yes
MEDQNIIQAPQKEVGK	16	Yes
MEDQNIIQAPQKEVGKGCDPDAHPK	25	No
MMNRTTPDQELVPASEPVWERPWSVEEIR	29	No
MMNRTTPDQELVPASEPVWERPWSVEEIRR	30	No
MNRTTPDQELVPASEPVWERPWSVEEIR	28	No
MNRTTPDQELVPASEPVWERPWSVEEIRR	29	No
NETKSSSQQDVILTTQDIFEDDIFATEAIKPSQK	34	No
NLKPSSETK	9	Yes
NLKPSSETKTQK	12	Yes
NLKPSSETKTQKGLFSDEEDSEDLFSSQSASNLK	34	No
NQKHPESIQGSK	12	Yes
NQKHPESIQGSKEK	14	Yes
QAGASVK	7	Yes
QAGASVKEESSSSKPGK	17	Yes
QAGASVKEESSSSKPGKK	18	Yes
QTLSLQAQR	9	No
QTLSLQAQREEK	12	No
QTLSLQAQREEKAK	14	No
QVDGLIR	7	Yes
QVDGLIRETK	10	Yes
QVDGLIRETKATDCR	15	Yes
RLAAQESSEAEDMSVPR	17	No
RPQTRAAR	8	Yes
RPQTRAARR	9	Yes
RRPQTRAAR	9	Yes
RSHGLESVPVLPGSGEAGVSFDLPAQADTLHSANK	35	Yes
RSRPTSFADELAAR	14	Yes
RSRPTSFADELAARIK	16	Yes
RSSQSWSLAADAGLLQFLQEFSQQTISR	28	Yes
RSSQSWSLAADAGLLQFLQEFSQQTISRTHEIK	33	Yes
RSSQSWSLAADAGR	14	No
RSSQSWSLAADAGRVYDEEVEEPVLK	26	No
RTPSDDEEDNLFAPPK	16	Yes

**Peptide sequence**	**Length (residues)**	**Also present in *WASHC2A***
SAQAAPEPR	9	Yes
SAQAAPEPRFEHK	13	Yes
SAQAAPEPRFEHKVSNIFDDPLNAFGGQ	28	Yes
SAQETVK	7	Yes
SAQETVKHSDLFSSSSPWDK	20	Yes
SAQETVKHSDLFSSSSPWDKGTKPR	25	Yes
SHGLESVPVLPGSGEAGVSFDLPAQADTLHSANK	34	Yes
SIFDDDMDDIFSTGIQAK	18	No
SIFDDDMDDIFSTGIQAKTTKPK	23	No
SIFDDDMDDIFSTGIQAKTTKPKSR	25	No
SKGEPRDSGTLQSQEAK	17	Yes
SLGHSRGEADLFDSGDIFSTGTGSQSVER	29	Yes
SLGHSRGEADLFDSGDIFSTGTGSQSVERTKPK	33	Yes
SPMFPALGEASSDDDLFQSAKPKPAK	26	Yes
SPMFPALGEASSDDDLFQSAKPKPAKK	27	Yes
SPYGPPPTGLFDDDDGDDDDDFFSAPHSKPSK	32	Yes
SPYGPPPTGLFDDDDGDDDDDFFSAPHSKPSKTGK	35	Yes
SPYGPPPTGLFDDDDGDDDDDFFSAPHSKPSKTR	34	No
SPYGPPPTGLFDDDDGDDDDDFFSAPHSKPSKTRK	35	No
SQPLVQEK	8	Yes
SQPLVQEKK	9	Yes
SQPLVQEKKR	10	Yes
SRPTSFADELAAR	13	Yes
SRPTSFADELAARIK	15	Yes
SRPTSFADELAARIKGDAMGR	21	No
SRSAQAAPEPR	11	Yes
SRSAQAAPEPRFEHK	15	Yes
SSQSWSLAADAGLLQFLQEFSQQTISR	27	Yes
SSQSWSLAADAGLLQFLQEFSQQTISRTHEIK	32	Yes
SSQSWSLAADAGLLQFLQEFSQQTISRTHEIKK	33	Yes
SSQSWSLAADAGR	13	No
SSQSWSLAADAGRVYDEEVEEPVLK	25	No
SSQSWSLAADAGRVYDEEVEEPVLKAEAEK	30	No
SSSQQDVILTTQDIFEDDIFATEAIKPSQK	30	No
SSSQQDVILTTQDIFEDDIFATEAIKPSQKTR	32	No
SSSQQDVILTTQDIFEDDIFATEAIKPSQKTREK	34	No
STGVFQDEELLFSHK	15	Yes
STGVFQDEELLFSHKLQK	18	Yes
STGVFQDEELLFSHKLQKDNDPDVDLFAGTK	31	Yes
SVDKKVESAK	10	Yes

**Peptide sequence**	**Length (residues)**	**Also present in *WASHC2A***
TDVAESEK	8	Yes
TDVAESEKEGLLTR	14	Yes
TDVAESEKEGLLTRSAQETVK	21	Yes
TEQEKTR	7	Yes
TEQEKTREQK	10	Yes
TGKVQSTADIFGDEEGDLFK	20	Yes
TGKVQSTADIFGDEEGDLFKEK	22	Yes
THEIKKQVDGLIR	13	Yes
TKEPSTR	7	Yes
TKEPSTRIGK	10	Yes
TKLLEPSVGSLFGDDEDDDLFSSAK	25	Yes
TKLLEPSVGSLFGDDEDDDLFSSAKSQPLVQEK	33	Yes
TKPKAKIAENPANPPVGGK	19	Yes
TKTVLSLFDEEEDK	14	Yes
TKTVLSLFDEEEDKMEDQNIIQAPQK	26	Yes
TLESNLFDDNIDIFADLTVKPK	22	Yes
TLESNLFDDNIDIFADLTVKPKEK	24	Yes
TLESNLFDDNIDIFADLTVKPKEKSK	26	Yes
TNPFPLLEDEDDLFTDQK	18	Yes
TNPFPLLEDEDDLFTDQKVK	20	Yes
TNPFPLLEDEDDLFTDQKVKK	21	Yes
TPSDDEEDNLFAPPK	15	Yes
TPSDDEEDNLFAPPKLTDEDFSPFGSGGGLFSGGK	35	Yes
TQKGLFSDEEDSEDLFSSQSASNLK	25	No
TQKGLFSDEEDSEDLFSSQSASNLKGASLLPGK	33	No
TQRVSLLFEDDVDSGGSLFGSPPTSVPPATK	31	Yes
TQRVSLLFEDDVDSGGSLFGSPPTSVPPATKK	32	Yes
TREQKEVDLIPK	12	Yes
TRKVQSTADIFGDEEGDLFK	20	No
TSLFEEDK	8	No
TSLFEEDKEDDLFAIAK	17	No
TSLFEEDKEDDLFAIAKDSQK	21	No
TTKPKSR	7	Yes
TTKPKSRSAQAAPEPR	16	Yes
TTPDQELVPASEPVWERPWSVEEIR	25	No
TTPDQELVPASEPVWERPWSVEEIRR	26	No
TVLSLFDEEEDK	12	Yes
TVLSLFDEEEDKMEDQNIIQAPQK	24	Yes
TVLSLFDEEEDKMEDQNIIQAPQKEVGK	28	Yes
VDEEPTNEEDNLFAPPK	17	Yes

**Peptide sequence**	**Length (residues)**	**Also present in *WASHC2A***
VDEEPTTLPSGEAKPR	16	Yes
VDEEPTTLPSGEAKPRK	17	Yes
VDEEPTTLPSGEAKPRKTLK	20	Yes
VEAKSIFDDDMDDIFSTGIQAK	22	No
VEAKSIFDDDMDDIFSTGIQAKTTKPK	27	No
VELILEPK	8	Yes
VELILEPKDLYIDRPLPYLIGSK	23	Yes
VESAKESLK	9	Yes
VESAKESLKFGR	12	Yes
VKKNETK	7	Yes
VQEAVNYGLQVLDSAFEQLDIK	22	Yes
VQSTADIFGDEEGDLFK	17	Yes
VQSTADIFGDEEGDLFKEK	19	Yes
VQSTADIFGDEEGDLFKEKAVASPEATVSQTDENK	35	Yes
VSLLFEDDVDSGGSLFGSPPTSVPPATK	28	Yes
VSLLFEDDVDSGGSLFGSPPTSVPPATKEGLLTR	34	Yes
VSLLFEDDVDSGGSLFGSPPTSVPPATKK	29	Yes
VSLLFEDDVDSGGSLFGSPPTSVPPATKKK	30	Yes
VSNIFDDPLNAFGGQ	15	Yes
VTLSYSK	7	No
VTLSYSKNLKPSSETK	16	No
VTLSYSKNLKPSSETKTQK	19	No
VVKKDHSVNSFK	12	No
VYDEEVEEPVLK	12	Yes
VYDEEVEEPVLKAEAEK	17	Yes
VYDEEVEEPVLKAEAEKTEQEK	22	Yes

All 302 candidate sequences are retained. Length is measured in amino-acid residues. *WASHC2A* matching treats I/L as equivalent. Absence from *WASHC2A* does not imply uniqueness across the human proteome or detection by CPTAC. I/L, isoleucine/leucine; CPTAC, Clinical Proteomic Tumor Analysis Consortium.

Table S13. Adjusted associations of continuous *WASHC2C* with survival

A. Primary associations

Cancer	Endpoint	N	Events	Adjusted HR (95% CI)	Nominal *p*	BH FDR
LUAD	OS	485	177	0.779 (0.584–1.038)	0.088055	0.264164
LUAD	PFI	485	197	0.875 (0.672–1.140)	0.321689	0.482533
LUAD	DSS	450	107	0.763 (0.529–1.099)	0.145755	0.291510
LUAD	DFI	287	85	0.773 (0.509–1.175)	0.228212	0.391220
BLCA	OS	399	174	NE	NE	NE
BLCA	PFI	400	171	1.122 (0.842–1.495)	0.432950	0.577266
BLCA	DSS	385	118	NE	NE	NE
BLCA	DFI	184	31	0.993 (0.528–1.869)	0.982679	1.000000
LIHC	OS	340	115	1.612 (1.177–2.208)	0.002914	0.034968
LIHC	PFI	341	163	1.344 (1.027–1.759)	0.031033	0.124134
LIHC	DSS	332	66	1.724 (1.122–2.650)	0.012986	0.077913
LIHC	DFI	294	131	1.253 (0.927–1.692)	0.142064	0.291510

HRs are per 1-unit increase in log2(TPM + 1), adjusted for age, sex and stage. NE = not estimable: BLCA stage I contained two patients and no OS/DSS events; N/events describe the intended complete-case sets. *p* values are rounded to six decimals. LIHC OS is the sole primary FDR < 0.05 result. PH *p* values are nominal diagnostics. OS, overall survival; PFI, progression-free interval; DSS, disease-specific survival; DFI, disease-free interval; HR, hazard ratio; CI, confidence interval; BH, Benjamini–Hochberg; FDR, false discovery rate; NE, not estimable. LUAD, lung adenocarcinoma; BLCA, bladder urothelial carcinoma; LIHC, liver hepatocellular carcinoma; TPM, transcripts per million; N, patients in the analysis set.

B. Proportional hazards diagnostics

Cancer	Endpoint	Gene PH *p*	Global PH *p*
LUAD	OS	0.255933	0.153768
LUAD	PFI	0.090462	0.008596
LUAD	DSS	0.078888	0.042806
LUAD	DFI	0.141658	0.368142
BLCA	OS	NE	NE
BLCA	PFI	0.587013	0.343663
BLCA	DSS	NE	NE
BLCA	DFI	0.945142	0.018965
LIHC	OS	0.276555	0.139223
LIHC	PFI	0.650077	0.290389
LIHC	DSS	0.007165	0.004993
LIHC	DFI	0.476725	0.205171

Schoenfeld residual score tests are nominal diagnostic tests. PH, proportional hazards; NE, not estimable. LIHC DSS violated gene-specific PH; global tests were significant for LUAD PFI/DSS, BLCA DFI, and LIHC DSS.

Table S14. Full multivariable Cox coefficients A. TCGA LUAD

Predictor	OS	PFI	DSS	DFI
WASHC2C	0.78 (0.58–1.04)<br>$p = 0.088055$	0.87 (0.67–1.14)<br>$p = 0.321689$	0.76 (0.53–1.10)<br>$p = 0.145755$	0.77 (0.51–1.17)<br>$p = 0.228212$
Age per 10 years	1.11 (0.95–1.29)<br>$p = 0.180616$	1.02 (0.89–1.18)<br>$p = 0.757690$	0.94 (0.78–1.14)<br>$p = 0.546359$	1.22 (0.98–1.54)<br>$p = 0.078550$
Male vs female	0.99 (0.73–1.34)<br>$p = 0.947862$	1.03 (0.78–1.37)<br>$p = 0.817619$	0.89 (0.60–1.31)<br>$p = 0.555945$	1.19 (0.77–1.84)<br>$p = 0.425355$
II vs I	2.32 (1.61–3.36)<br>$p = 7.27 \times 10^{-6}$	2.17 (1.56–3.01)<br>$p = 4.24 \times 10^{-6}$	3.03 (1.90–4.86)<br>$p = 3.71 \times 10^{-6}$	1.66 (1.03–2.65)<br>$p = 0.035902$
III vs I	3.26 (2.22–4.79)<br>$p = 1.74 \times 10^{-9}$	1.96 (1.32–2.89)<br>$p = 7.69 \times 10^{-4}$	3.25 (1.94–5.44)<br>$p = 7.11 \times 10^{-6}$	0.79 (0.34–1.85)<br>$p = 0.588883$
IV vs I	3.82 (2.17–6.71)<br>$p = 3.22 \times 10^{-6}$	2.28 (1.28–4.08)<br>$p = 0.005287$	4.63 (2.30–9.34)<br>$p = 1.86 \times 10^{-5}$	—

Each cell reports adjusted HR (95% CI), followed by the two-sided nominal *p* value. All covariates in each endpoint column were fitted jointly; patient and event counts are in Table S13.

WASHC2C is continuous, with a 1-unit increase in log2(TPM + 1). Stage contrasts use stage I as the reference for LUAD/LIHC and stage II for BLCA. — = stage IV is absent from the DFI analysis set; no coefficient was fitted. NE = the full categorical-stage model was not estimable. HRs/CIs in this table are rounded to two decimals; *p* values to six decimals or in scientific notation. FDR correction applies to *WASHC2C* in Table S13, not to these descriptive covariate coefficients.

Stage estimates require caution: BLCA stage I included only two patients, and BLCA DFI had 31 events across five coefficients (6.2 events per coefficient). LIHC stage IV estimates also have wide confidence intervals. Table S13 reports PH diagnostics for *WASHC2C* and the full model.

OS, overall survival; PFI, progression-free interval; DSS, disease-specific survival; DFI, disease-free interval; HR, hazard ratio; CI, confidence interval; BH, Benjamini–Hochberg; FDR, false discovery rate; NE, not estimable. PH, proportional hazards; TPM, transcripts per million; LUAD, lung adenocarcinoma; BLCA, bladder urothelial carcinoma; LIHC, liver hepatocellular carcinoma.

Table S14. Full multivariable Cox coefficients B. TCGA BLCA

Predictor	OS	PFI	DSS	DFI
WASHC2C	NE	1.12 (0.84–1.50)<br>$p = 0.432950$	NE	0.99 (0.53–1.87)<br>$p = 0.982679$
Age per 10 years	NE	1.09 (0.94–1.26)<br>$p = 0.271027$	NE	0.85 (0.61–1.18)<br>$p = 0.333499$
Male vs female	NE	0.88 (0.63–1.24)<br>$p = 0.471283$	NE	0.79 (0.36–1.76)<br>$p = 0.566332$
I vs II	NE	1.97 (0.27–14.43)<br>$p = 0.504675$	NE	5.73 (0.72–45.84)<br>$p = 0.100010$
III vs II	NE	1.32 (0.86–2.02)<br>$p = 0.211357$	NE	1.58 (0.75–3.31)<br>$p = 0.225503$
IV vs II	NE	3.00 (2.03–4.44)<br>$p = 3.78 \times 10^{-8}$	NE	—

Each cell reports adjusted HR (95% CI), followed by the two-sided nominal *p* value. All covariates in each endpoint column were fitted jointly; patient and event counts are in Table S13.

WASHC2C is continuous, with a 1-unit increase in log2(TPM + 1). Stage contrasts use stage I as the reference for LUAD/LIHC and stage II for BLCA. — = stage IV is absent from the DFI analysis set; no coefficient was fitted. NE = the full categorical-stage model was not estimable. HRs/CIs in this table are rounded to two decimals; *p* values to six decimals or in scientific notation. FDR correction applies to *WASHC2C* in Table S13, not to these descriptive covariate coefficients.

Stage estimates require caution: BLCA stage I included only two patients, and BLCA DFI had 31 events across five coefficients (6.2 events per coefficient). LIHC stage IV estimates also have wide confidence intervals. Table S13 reports PH diagnostics for *WASHC2C* and the full model.

OS, overall survival; PFI, progression-free interval; DSS, disease-specific survival; DFI, disease-free interval; HR, hazard ratio; CI, confidence interval; BH, Benjamini–Hochberg; FDR, false discovery rate; NE, not estimable. PH, proportional hazards; TPM, transcripts per million; LUAD, lung adenocarcinoma; BLCA, bladder urothelial carcinoma; LIHC, liver hepatocellular carcinoma.

Table S14. Full multivariable Cox coefficients. C. TCGA LIHC

Predictor	OS	PFI	DSS	DFI
WASHC2C	1.61 (1.18–2.21)<br>$p = 0.002914$	1.34 (1.03–1.76)<br>$p = 0.031033$	1.72 (1.12–2.65)<br>$p = 0.012986$	1.25 (0.93–1.69)<br>$p = 0.142064$
Age per 10 years	1.16 (1.00–1.35)<br>$p = 0.050007$	0.98 (0.87–1.10)<br>$p = 0.700790$	1.02 (0.85–1.24)<br>$p = 0.810670$	1.00 (0.87–1.16)<br>$p = 0.967514$
Male vs female	0.93 (0.63–1.37)<br>$p = 0.709447$	1.03 (0.74–1.43)<br>$p = 0.879086$	0.94 (0.56–1.60)<br>$p = 0.823095$	1.22 (0.84–1.78)<br>$p = 0.292545$
II vs I	1.23 (0.75–2.02)<br>$p = 0.420009$	1.78 (1.21–2.64)<br>$p = 0.003707$	1.40 (0.69–2.84)<br>$p = 0.352807$	1.62 (1.05–2.51)<br>$p = 0.029292$
III vs I	2.47 (1.60–3.80)<br>$p = 4.05 \times 10^{-5}$	2.54 (1.74–3.70)<br>$p = 1.40 \times 10^{-6}$	4.00 (2.26–7.08)<br>$p = 1.97 \times 10^{-6}$	2.73 (1.80–4.16)<br>$p = 2.70 \times 10^{-6}$
IV vs I	5.89 (1.77–19.65)<br>$p = 0.003878$	6.15 (1.87–20.22)<br>$p = 0.002799$	10.13 (2.24–45.78)<br>$p = 0.002622$	—

Each cell reports adjusted HR (95% CI), followed by the two-sided nominal *p* value. All covariates in each endpoint column were fitted jointly; patient and event counts are in Table S13.

WASHC2C is continuous, with a 1-unit increase in log2(TPM + 1). Stage contrasts use stage I as the reference for LUAD/LIHC and stage II for BLCA. — = stage IV is absent from the DFI analysis set; no coefficient was fitted. NE = the full categorical-stage model was not estimable. HRs/CIs in this table are rounded to two decimals; *p* values to six decimals or in scientific notation. FDR correction applies to *WASHC2C* in Table S13, not to these descriptive covariate coefficients.

Stage estimates require caution: BLCA stage I included only two patients, and BLCA DFI had 31 events across five coefficients (6.2 events per coefficient). LIHC stage IV estimates also have wide confidence intervals. Table S13 reports PH diagnostics for *WASHC2C* and the full model.

OS, overall survival; PFI, progression-free interval; DSS, disease-specific survival; DFI, disease-free interval; HR, hazard ratio; CI, confidence interval; BH, Benjamini–Hochberg; FDR, false discovery rate; NE, not estimable. PH, proportional hazards; TPM, transcripts per million; LUAD, lung adenocarcinoma; BLCA, bladder urothelial carcinoma; LIHC, liver hepatocellular carcinoma.

Table S15. Median split *WASHC2C* survival sensitivity analysis

High expression was defined as above the cohort median; low expression as at or below the median. Thresholds were fixed before covariate/endpoint exclusions: LUAD 4.1625, BLCA 4.0240, and LIHC 3.6015 on the log2(TPM + 1) scale. Models retained age, sex, and categorical stage; FDR was adjusted across the 12 median-split tests.

Cancer	Endpoint	N / events	High vs low HR (95% CI)	Nominal *p*	BH FDR
LUAD	OS	485/177	0.749 (0.554–1.012)	0.059691	0.238764
LUAD	PFI	485/197	0.814 (0.614–1.079)	0.152138	0.354174
LUAD	DSS	450/107	0.761 (0.517–1.120)	0.166500	0.354174
LUAD	DFI	287/85	0.758 (0.492–1.166)	0.206601	0.354174
BLCA	OS	399/174	NE	NE	NE
BLCA	PFI	400/171	1.218 (0.899–1.651)	0.203193	0.354174
BLCA	DSS	385/118	NE	NE	NE
BLCA	DFI	184/31	1.400 (0.684–2.864)	0.356726	0.475634
LIHC	OS	340/115	1.495 (1.020–2.192)	0.039470	0.238764
LIHC	PFI	341/163	1.184 (0.861–1.629)	0.299648	0.449471
LIHC	DSS	332/66	1.684 (1.011–2.802)	0.045087	0.238764
LIHC	DFI	294/131	1.126 (0.791–1.604)	0.509444	0.611333

No median-split association met FDR < 0.05. NE has the same definition as in Table S13. OS, overall survival; PFI, progression-free interval; DSS, disease-specific survival; DFI, disease-free interval; HR, hazard ratio; CI, confidence interval; BH, Benjamini–Hochberg; FDR, false discovery rate; NE, not estimable. LUAD, lung adenocarcinoma; BLCA, bladder urothelial carcinoma; LIHC, liver hepatocellular carcinoma; TPM, transcripts per million; N, patients.

Table S16. GSE31210 inclusion characteristics and endpoint counts

Characteristic	Reported value
Deposited profiles	246
Tumors scored	226
Non-tumor profiles omitted	20
Source exclusions from survival	22
Survival eligible patients	204
Deaths	30
Source-defined relapse events	54
Age median (IQR), years	61 (55–65)
Female / male	109 / 95
Stage I / II	162 / 42
Never / ever smokers	105 / 99
Measurable signature genes	48 / 50

The 22 source-defined exclusions concerned incomplete resection or adjuvant therapy. Age, sex, stage, and smoking were complete in the 204 eligible patients. IQR, interquartile range. The source-defined relapse endpoint does not add death as an event.

Table S17. Continuous GSVA signature scores and survival in GSE31210

Endpoint	Model	N / events	HR (95% CI)	Nominal *p*
OS	Univariate	204 / 30	0.424 (0.283–0.636)	3.4283084×10^{-5}
OS	Adjusted	204 / 30	0.461 (0.303–0.703)	3.1518803×10^{-4}
Relapse	Univariate	204 / 54	0.483 (0.361–0.645)	8.3446471×10^{-7}
Relapse	Adjusted	204 / 54	0.516 (0.380–0.701)	2.3129707×10^{-5}

HRs are per one SD increase in score. Adjusted OS: stage; adjusted relapse: age, sex, stage and smoking. CIs and *p* values use Wald statistics. Relapse follows the source definition and is not assumed equivalent to TCGA PFS, DFI or DSS. GSVA, gene set variation analysis; SD, standard deviation; OS, overall survival; HR, hazard ratio; CI, confidence interval; PFS, progression-free survival; DFI, disease-free interval; DSS, disease-specific survival. FDR, false discovery rate; TCGA, The Cancer Genome Atlas. No revised FDR values were calculated for the retained set.

S4. Supplementary Figures

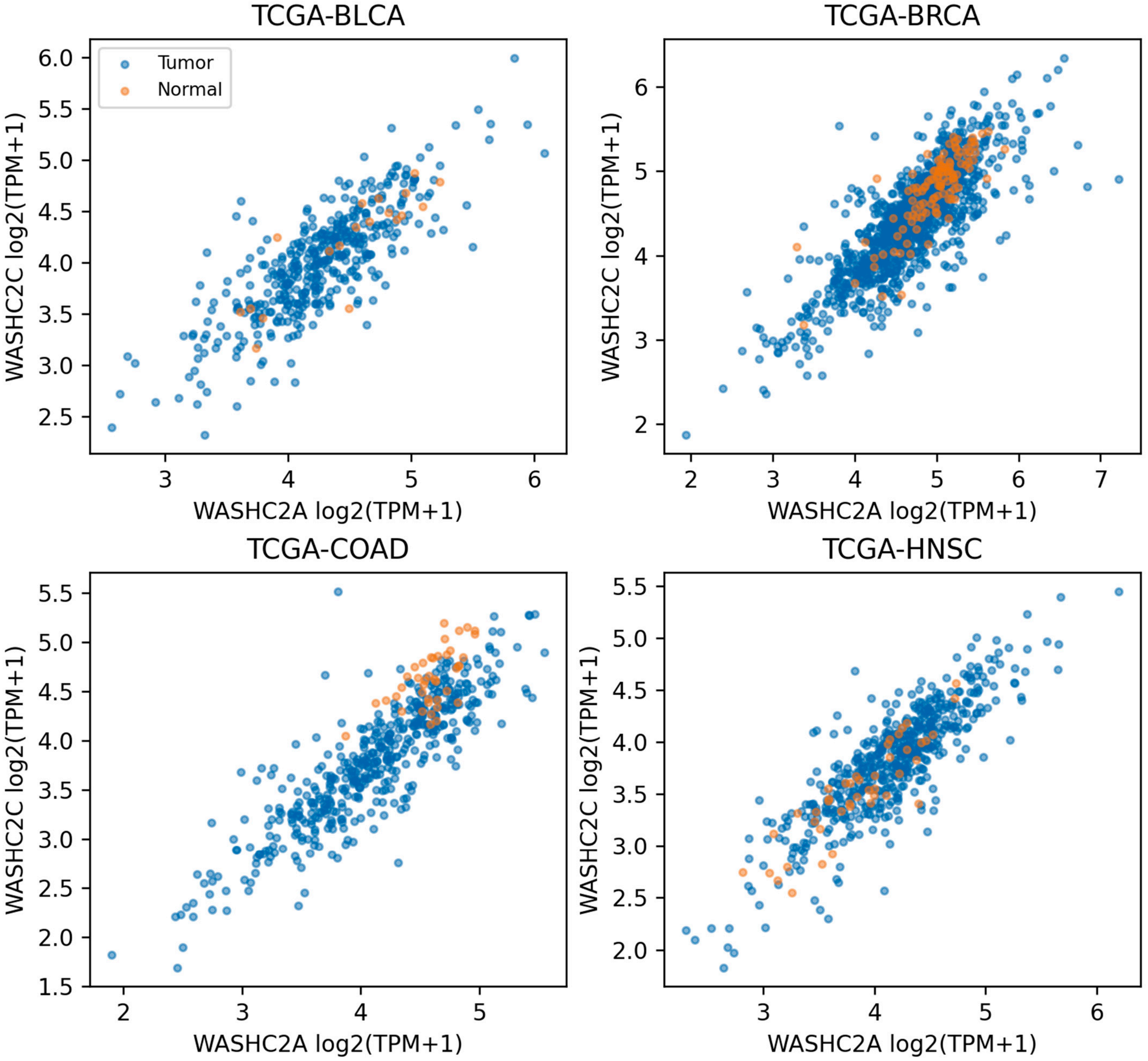

Figure S1. Harmonized *WASHC2A–WASHC2C* co-expression. (A) BLCA, BRCA, COAD, and HNSC. Each point represents a primary tumor (blue) or normal specimen (orange), with both genes extracted from the same selected GDC STAR file. Both axes use log2(TPM + 1). The primary tests are tumor-only Spearman correlations, with BH correction across eight cancers (Table S1); the pooled visual patterns do not constitute the primary test. BLCA, bladder urothelial carcinoma; BRCA, breast invasive carcinoma; COAD, colon adenocarcinoma; HNSC, head and neck squamous cell carcinoma; GDC, Genomic Data Commons; TPM, transcripts per million; BH, Benjamini–Hochberg. Panel B continues on the next page.

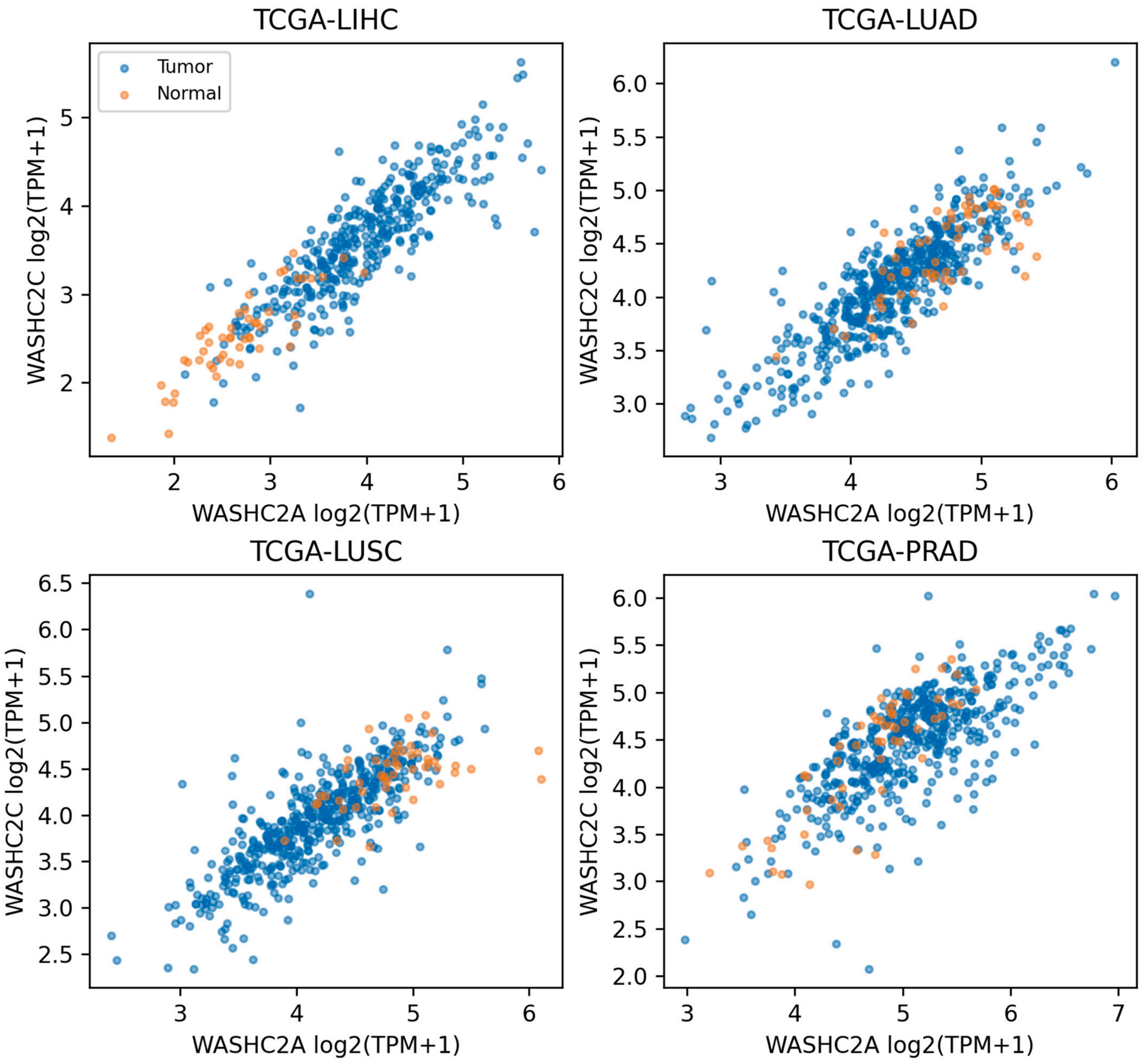

Figure S1 continued. (B) LIHC, LUAD, LUSC, and PRAD. Blue points show tumors and orange points show normal specimens. Horizontal axes denote *WASHC2A* log2(TPM + 1); vertical axes denote *WASHC2C* log2(TPM + 1). Tumor-only Spearman tests and BH-adjusted FDR values are reported in Table S1. LIHC, liver hepatocellular carcinoma; LUAD, lung adenocarcinoma; LUSC, lung squamous cell carcinoma; PRAD, prostate adenocarcinoma; TPM, transcripts per million; BH, Benjamini–Hochberg; FDR, false discovery rate.

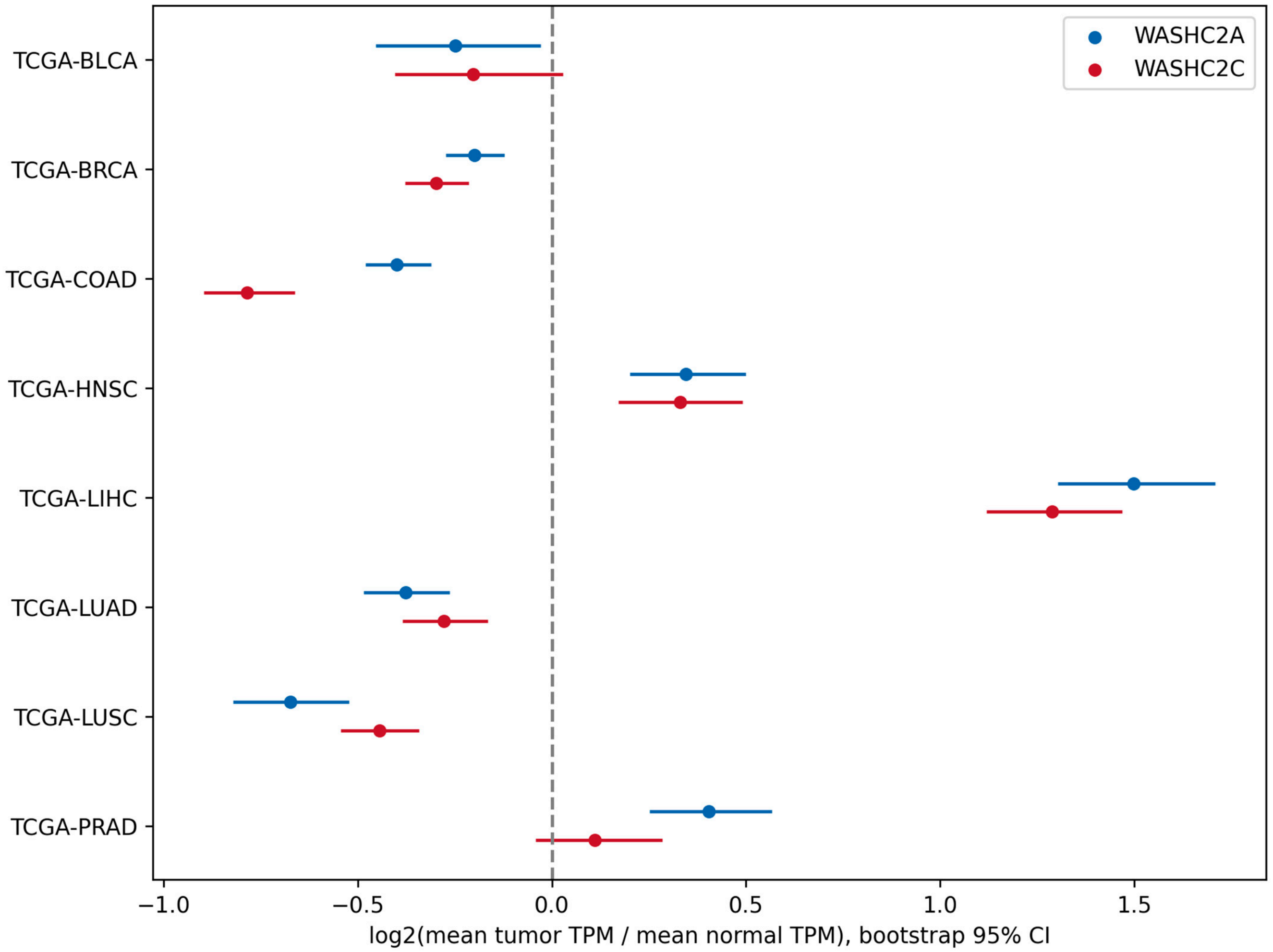

Figure S2. Tumor–normal effects. Points are log2(mean tumor TPM/mean normal TPM); intervals are descriptive 95% percentile confidence intervals from 2,000 separate-group bootstrap replicates. Blue: *WASHC2A*; red: *WASHC2C*. The dashed line denotes zero. BH-adjusted Mann–Whitney tests assess distributions and are reported in Table S2; these mean-ratio intervals do not imply statistical significance. Matched-pair sensitivity results are reported in Table S3. TPM, transcripts per million; CI, confidence interval; BH, Benjamini–Hochberg. BLCA, bladder urothelial carcinoma; BRCA, breast invasive carcinoma; COAD, colon adenocarcinoma; HNSC, head and neck squamous cell carcinoma; LIHC, liver hepatocellular carcinoma; LUAD, lung adenocarcinoma; LUSC, lung squamous cell carcinoma; PRAD, prostate adenocarcinoma.

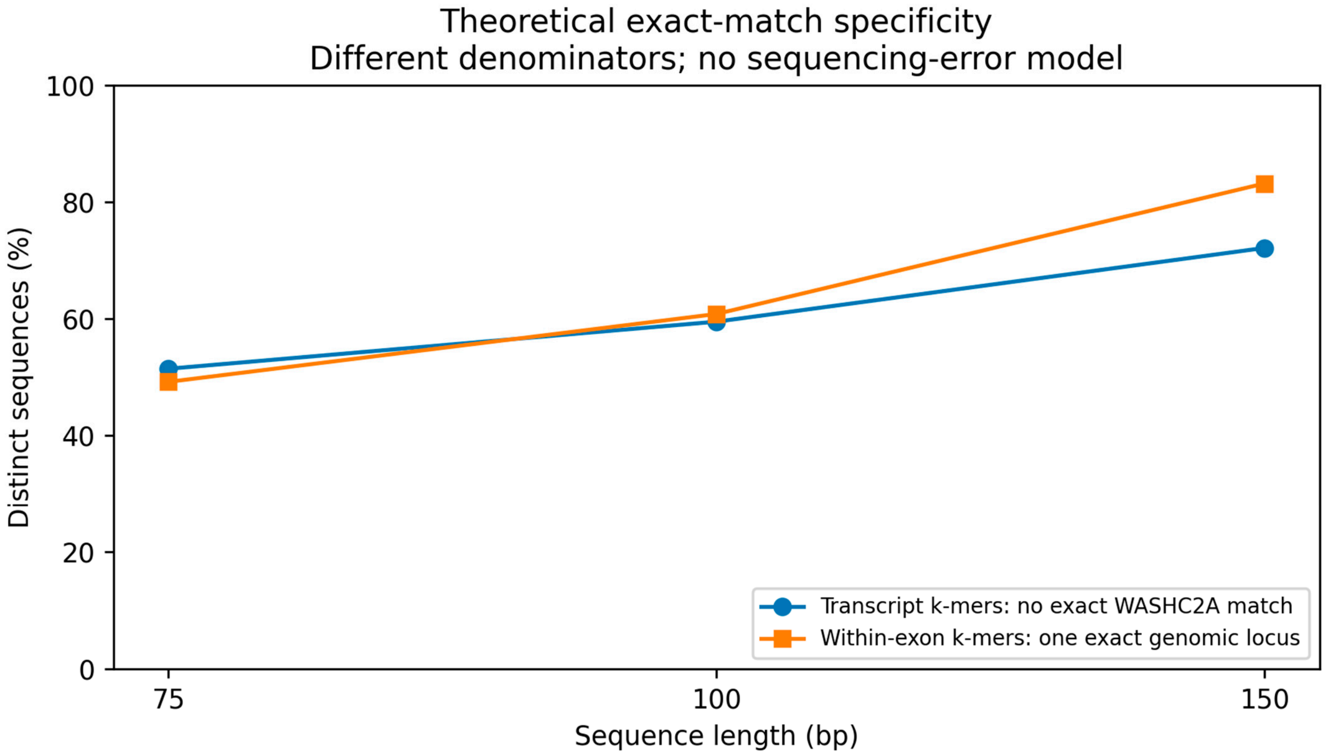

Figure S3. Theoretical sequence specificity. Distinct *WASHC2C* transcript sequences without an exact *WASHC2A* transcript match (blue) and distinct within-exon sequences with one exact genomic match (orange). GRCh38 primary assembly, both strands, zero mismatches. Each series uses its own denominator (Table S6). These are sequence-level proportions, not observed patient-read mapping rates. Blue circles denote transcript sequences; orange squares denote within-exon genomic sequences. Lines connect the three evaluated lengths and do not represent an inferential model. bp, base pairs.

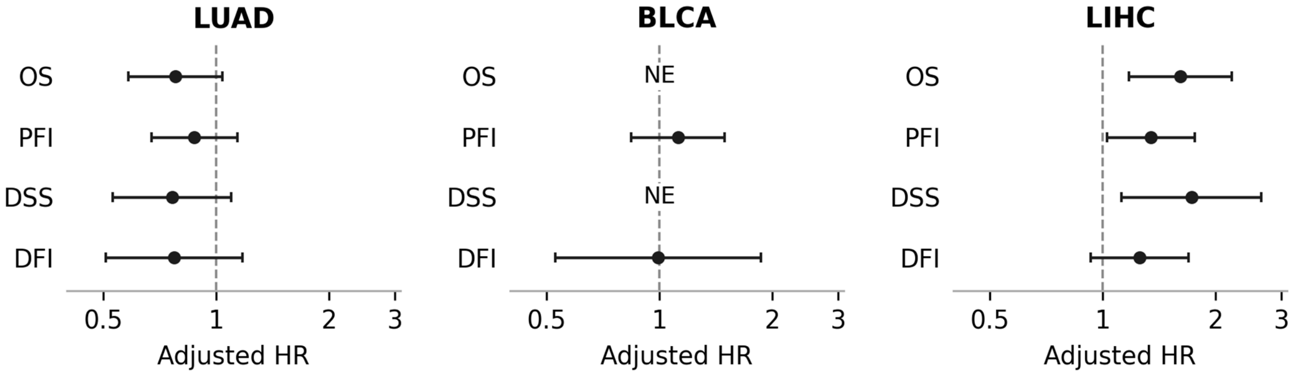

Figure S4. Adjusted *WASHC2C* hazard ratios in primary TCGA models. Left to right: LUAD, BLCA, and LIHC. Points and horizontal lines show adjusted HRs and Wald 95% CIs per one-unit increase in log2(TPM + 1), controlling for age, sex, and categorical stage. Dashed lines indicate HR = 1; axes are logarithmic. Primary BH correction covered 12 planned gene tests (Table S13). NE models have no estimate. LIHC DSS requires caution because of a gene-specific PH violation. LUAD, lung adenocarcinoma; BLCA, bladder urothelial carcinoma; LIHC, liver hepatocellular carcinoma; OS, overall survival; PFI, progression-free interval; DSS, disease-specific survival; DFI, disease-free interval; HR, hazard ratio; CI, confidence interval; NE, not estimable; PH, proportional hazards; TPM, transcripts per million.

A. Overall survival

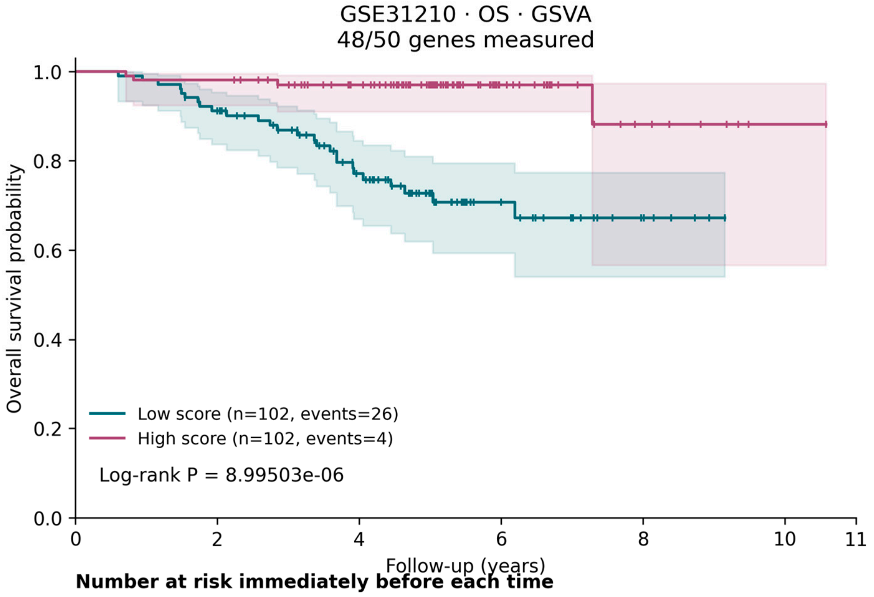

Number at risk immediately before each time

	0	2	4	6	8	10	11
Low	102	90	59	20	7	0	0
High	102	100	82	23	7	1	0

B. Source defined relapse endpoint

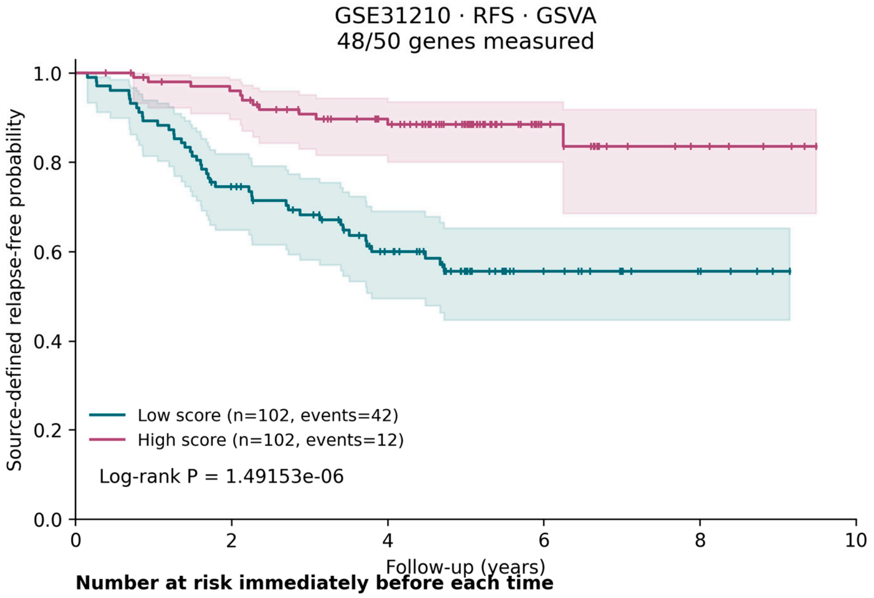

Number at risk immediately before each time

	0	2	4	6	8	10
Low	102	74	47	15	6	0
High	102	94	75	19	6	0

Figure S5. Kaplan–Meier analyses of the GSVA signature in GSE31210. (A) Overall survival; (B) source-defined relapse. High scores (magenta) were above the eligible-cohort median (0.01935893); low scores (teal) were at or below it. Each group contained 102 patients. High/low groups had 4/26 deaths and 12/42 relapse events. Shading shows 95% CIs; ticks indicate censoring; risk tables report patients at risk immediately before each time point. Two-sided nominal log-rank *p* values were 8.99503×10^{-6} for OS and 1.49153×10^{-6} for relapse. Scoring used 48/50 predefined genes. GSVA, gene set variation analysis; OS, overall survival; RFS, the source-defined relapse endpoint; CI, confidence interval.

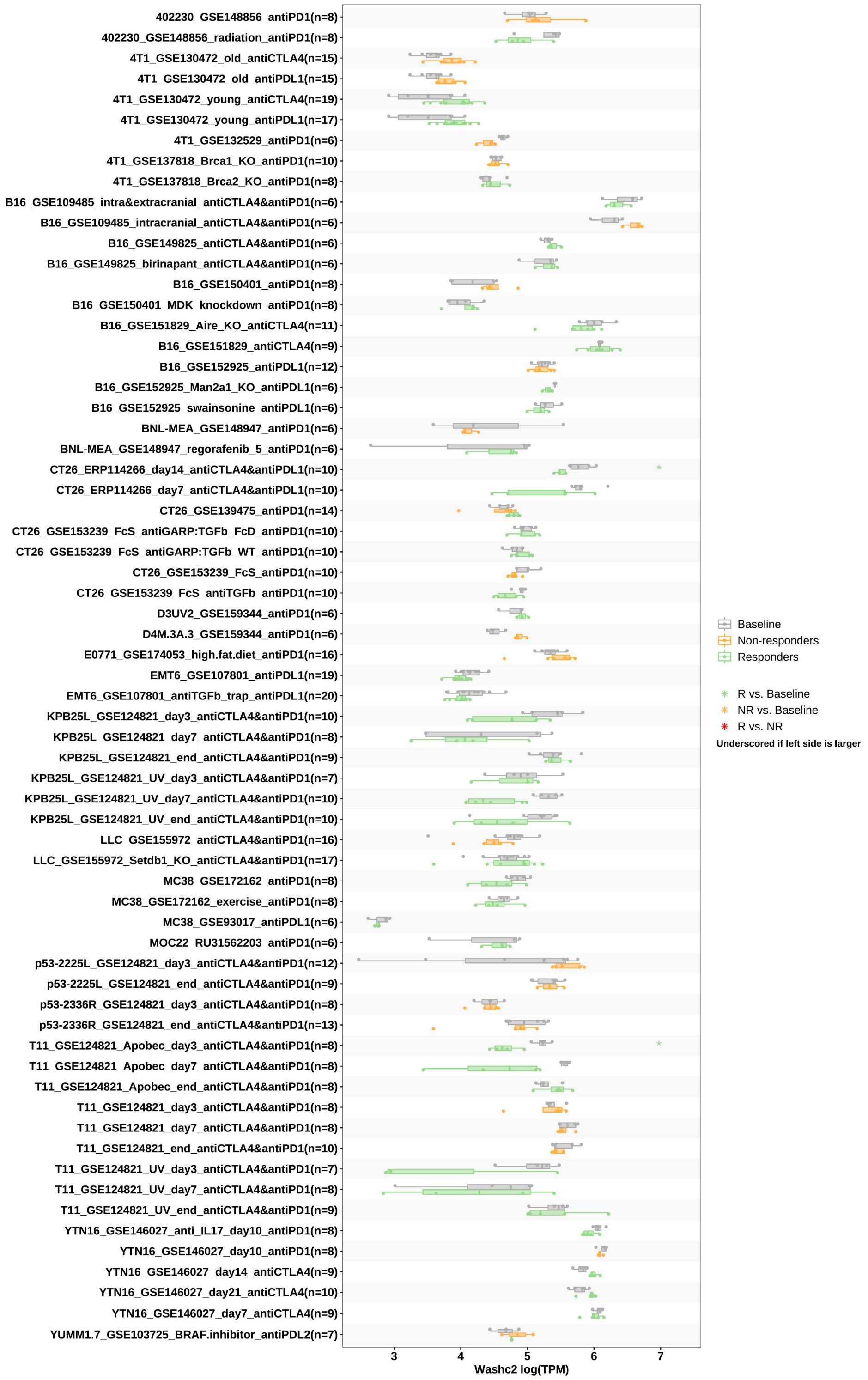

Figure S6. Exploratory murine *Washc2* response groups in TISMO. The complete supplied source graphic is shown. Gray, orange, and green denote baseline, non-responders (NR), and responders (R). The horizontal axis is *Washc2* log(TPM), as labeled in the source. Rows identify the model, dataset, treatment, and source sample count. DESeq2 pairwise contrasts with BH correction form the source framework. Green, orange, and red asterisks denote R versus baseline, NR versus baseline, and R versus NR, respectively; underlining indicates that the left-side group is larger. Exact significance thresholds and response-group counts were not supplied. These findings provide exploratory mouse context only. TISMO, Tumor Immune Syngeneic MOuse; TPM, transcripts per million; BH, Benjamini–Hochberg. Enlarged consecutive views follow.

S5. Reproducibility and Source Availability

These Supplementary Materials document the reported analysis specifications, retained numerical results, locked gene identities, and available figures. Reproducibility limitations differ by analytical module and are summarized below. No new statistical analyses were performed when organizing these materials.

The original eight-cancer expression matrix, file-level download manifest, mapping logs, sequence FASTA/GTF files and checksums, and sequence-analysis scripts were not supplied. The retained expression and sequence tables document reported results rather than an independently repeated analysis. Observed cohort-specific CPTAC peptide exports, protein-group assignments, identification q values, and matching search FASTA versions were also unavailable. Missing observed evidence does not imply zero peptides.

No missing code, assay identifier, statistical threshold, or historical setting has been reconstructed. UCEC reconciliation methods and results are specified in Sections S1.6 and S2.5.

The supplied materials report Python 3.12.14, NumPy 2.3.5, pandas 2.2.3, SciPy 1.17.0, matplotlib 3.10.8, openpyxl 3.1.5, and python-docx 1.2.0 for the TCGA Cox workflow, and GSEApy 1.3.1 and lifelines 0.30.3 for the external scoring workflow. No public repository DOI is asserted.
